# An iterative synthesis of poly-substituted indole oligomers reveals a short effective conjugation length in eumelanin model compounds†

**DOI:** 10.1039/d4sc08610d

**Published:** 2025-02-05

**Authors:** Haiyan Huang, Lilia Kinziabulatova, Anju Manickoth, Yiming Zhang, Marisa A. Barilla, Lluís Blancafort, Bern Kohler, Jean-Philip Lumb

**Affiliations:** a Department of Chemistry, McGill University 801 Sherbrooke Street West Montreal QC H3A 0B8 Canada jean-philip.lumb@mcgill.ca; b Department of Chemistry and Biochemistry, The Ohio State University 100W. 18^th^ Avenue Columbus OH 43210 USA kohler.40@osu.edu; c Institut de Química Computacional, Departament de Química, Universitat de Girona Girona 17003 Spain lluis.blancafort@udg.edu

## Abstract

Eumelanin is a multifunctional biomaterial that colors the skin, hair and eyes of mammals. Despite years of effort, its molecular structure remains unknown, limiting our understanding of its biological function and the design of synthetic mimics. In an effort to address this challenge, we report an Iterative Chain Growth (ICG) of well-defined 5,6-dihydroxyindole (DHI) model compounds that provides direct, experimental evidence of a short effective conjugation length in the resulting oligomers. Our ICG highlights the C2-selective borylation of N–H indoles in complex settings, and the utility of Suzuki–Miyaura Coupling (SMC) to grow the chain. The resulting C2–C7′ linkage is installed selectively with good yields, affording products with up to 5-indole units. Access to these oligomers allows us to probe how DHI chain extension contributes to the emergence of sun screening in eumelanin. Our oligomers guarantee the absence of oxidized by-products that may otherwise complicate analysis, without substantially altering the photophysics of the indolic-backbone. Steady-state absorption and emission spectroscopy coupled with excited-state calculations reveal pronounced vibronic structure and excited state planarization, but only a moderate red shift with increasing chain length because of poor orbital coupling between adjoined π-systems. We conclude that eumelanin's characteristic ability to absorb visible light does not derive from long chains of fully reduced DHI sub-units. Our work takes an important step towards a more systematic exploration of eumelanin's structure through iterative synthesis, with the long-term goal of explaining the molecular origins of its properties.

## Abstract 2

An iterative synthesis of poly-substituted indoles reveals a short effective conjugation length in the corresponding oligomers. Combined with DFT calculations, these results provide insights into the emergence of melanin's photophysical properties.

## Graphical abstract

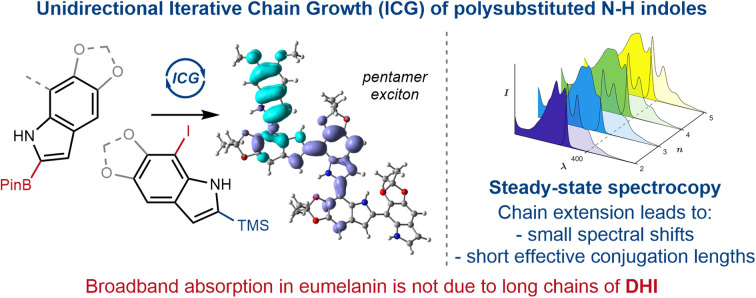

## Introduction

**Fig. 1:**
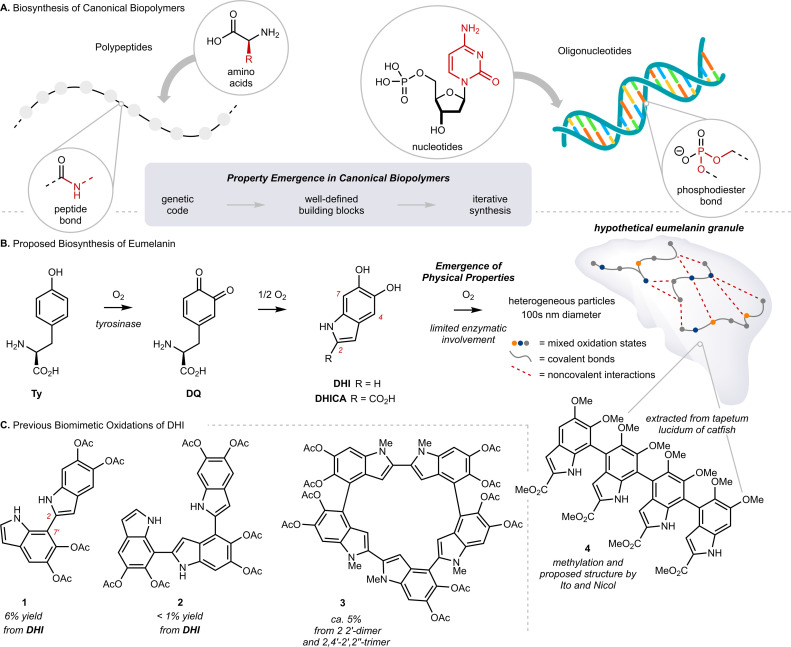
(A) Biosynthesis of canonical biopolymers *via* enzymatically controlled iterative synthesis. (B) Proposed biosynthesis of eumelanin; Ty: l-tyrosine, DQ: l-dopaquinone, DHI: 5,6-dihydroxyindole, DHICA: 5,6-dihydroxyindole-2-carboxylic acid. (C) Characterization of products produced by biomimetic oxidations of 5,6-dihydroxyindole (DHI) and related compounds.

Eumelanin is the black or brown pigment derived from l-tyrosine (Ty) that colors the skin, hair and eyes of mammals. It is known primarily as a sunscreen that protects DNA from light-induced damage, but the full complement of its physiological roles remains unclear.^1^ In addition to sun screening, eumelanin possesses a number of interesting properties, including paramagnetism, redox activity, metal chelation, and conductivity.^2^ The molecular origin of these properties, namely how they emerge from Ty, remains largely unknown.^3^ This is in stark contrast to our understanding of property emergence in canonical biopolymers, such as polypeptides or oligonucleotides, where the origin of function can be traced to a genetic code through a tightly regulated, iterative assembly of building blocks (Fig. 1A). By comparison, eumelanin is produced with little enzymatic control, and the structure of Ty changes markedly over the course of the process.^4^ The biosynthesis of eumelanin is triggered by *ortho*-oxygenation of Ty to l-dopaquinone (DQ) (Fig. 1B and Scheme S1†). While this first and rate-limiting step is catalyzed by the enzyme tyrosinase, the subsequent steps can occur spontaneously once DQ has exited the active site.^4*c*^ These steps include the oxidative cyclization of DQ to either 5,6-dihydroxyindole (DHI) or 5,6-dihydroxyindole-2-carboxylic acid (DHICA), followed by their continued oxidative coupling to nanometer sized granules. The details of this oxidation remain unclear, but biaryl bonds between sp^2^-hybridized carbons are believed to extend conjugation across multiple indole units, resulting in profound consequences for their optoelectronic properties.^5^ Efforts to identify discrete molecules from within eumelanin granules have faced many analytical challenges. The granules are heterogeneous, their components are poorly soluble, and there is extensive cross-linking to other biomolecules, such as lipids and proteins.^6^ Nevertheless, in the 1970s, Ito and Nicol isolated a tetramer of DHICA from the tapetum lucidum of catfish that they assigned as structure 4, strengthening the preeminent structural model of a poly-substituted indole oligomer, linked through biaryl bonds (Fig. 1B).^7^

Efforts to synthesize eumelanin *via* biomimetic oxidations of DHI, DHICA and related monomers have produced a range of materials. Many have similar physical properties to natural eumelanin and have generated considerable interest.^1*d*,8^ An illustrative example comes from synthetic eumelanin granules recently prepared by Gianneschi and co-workers, who have demonstrated their promising effects in healing skin-wounds.^9^ In the 1980s, Prota and d'Ischia investigated the products of biomimetic oxidations of DHI and DHICA,^10^ characterizing a number of covalently linked oligomers, with bonding primarily at C2, C4 and C7 (indole numbering) up to 5-units in length (Fig. 1C, 1–3).^11^ These studies provided important insights into the mechanism of oxidative polymerization, but the relatively poor selectivity, low yields and challenging purifications restricted their preparative utility. Correspondingly, there have been efforts to synthesize similar molecules by non-biomimetic routes,^8*d*,12^ culminating in 2010 with Manini's synthesis of a C2–C7′ linked trimer by consecutive use of Sonogashira coupling and Cu-catalyzed cyclization.^13*a*^ Since then, however, the *de novo* synthesis of higher-order oligomers has not been reported,^13*b*^ and more generally, there have only been limited studies exploring property emergence using these previously prepared materials.^14^

**Fig. 2:**
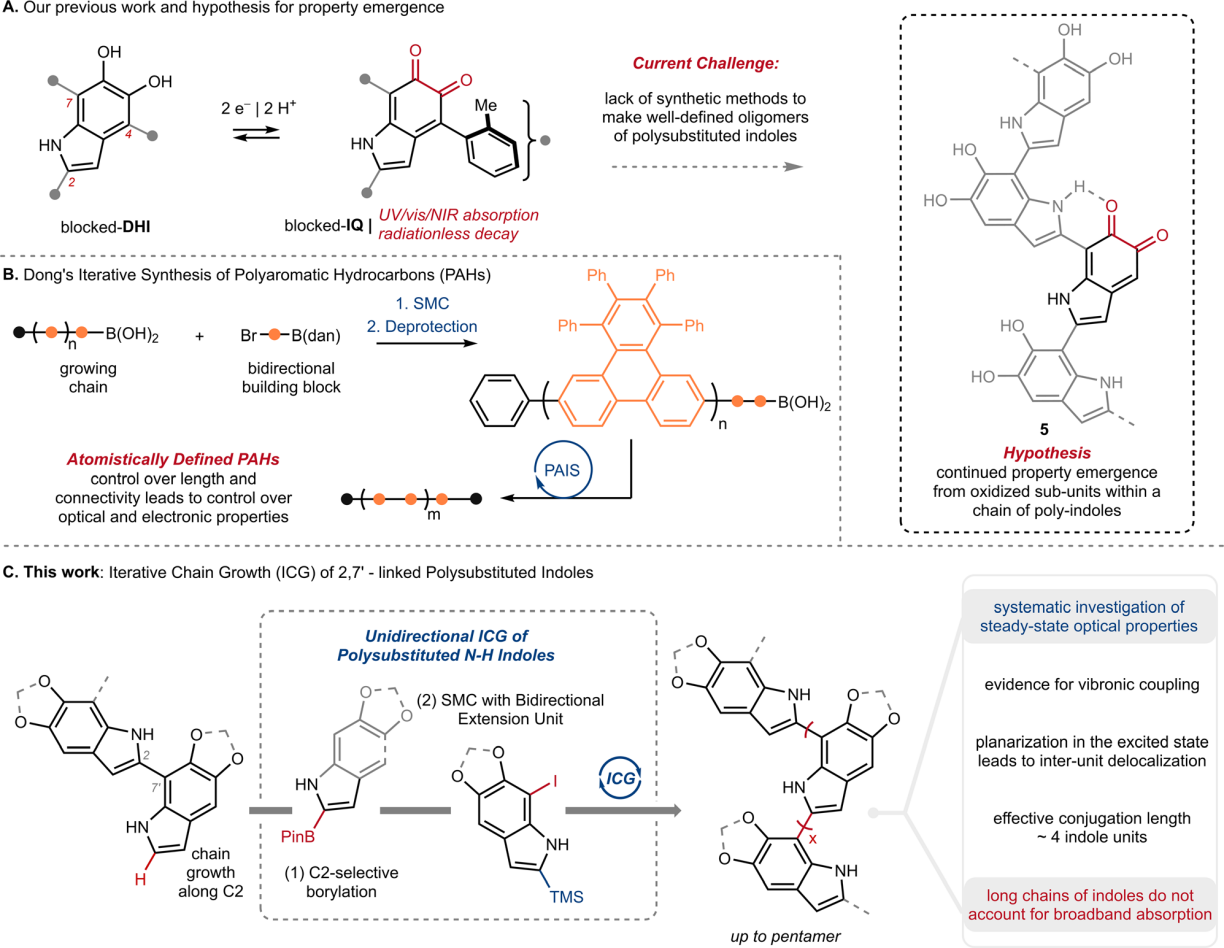
(A) Our previous work on the first isolable 5,6-indolequinone using a blocking group strategy, and our hypothesis that eumelanin's properties might emerge when an indolequinone is embedded within a chain of poly-substituted indoles; IQ: 5,6-indolequinone, DHI: 5,6-dihydroxyindole. (B) Dong and co-workers'^17^ iterative synthesis of polyaromatic hydrocarbons (PAHs); PAIS: protecting-group-aided iterative synthesis, SMC: Suzuki–Miyaura Coupling, dan: 1,8-diaminonapththalene. (C) This work: Iterative Chain Growth (ICG) of 2,7′-linked poly-substituted indoles and a systematic investigation of steady-state optical properties.

The knowledge gap surrounding eumelanin's structure has motivated our investigation of well-defined model compounds, with the long-term goal of identifying discrete structural units or chromophores that explain eumelanin's emergent properties. To this end, we recently reported the synthesis and characterization of the first isolable 5,6-indolequinone (blocked-IQ), demonstrating that it exhibits key properties of eumelanin, such as visible light absorption and ultrafast nonradiative decay (Fig. 2A).^15^ While these findings highlight the importance of oxidized subunits within eumelanin granules, our study also revealed that an isolated IQ unit does not fully replicate all of eumelanin's properties, such as monotonic decay. We hypothesized that a more accurate model might emerge if IQ were embedded within a chain of poly-substituted indoles (see hypothetical structure 5 in Fig. 2A), where its properties would be affected by neighboring groups.^16^ Testing this hypothesis required a robust synthetic strategy that could provide covalently linked indoles with precision over chain length and regiochemistry. We were inspired by recent work of Dong and co-workers (Fig. 2B),^17^ whose iterative synthesis of polyaromatic hydrocarbons (PAHs) has provided new insights into the emergence of properties in these carbon-rich materials.^18^ We believe that a similar approach to understanding eumelanin could be equally rewarding, provided we address the synthetic challenges of working with highly-substituted and electron rich N–H indoles.

In the present study, we take an important step towards this goal by describing an iterative chain growth (ICG) of DHI derivatives that provides eumelanin model compounds up to 5 units in length (Fig. 2C). The process is regioselective for the C2–C7′ linkage, and showcases the iridium (Ir) catalyzed C2–H borylation of indoles as a particularly effective tool for unidirectional chain-growth.^19^ Our work complements previous examples of iterative synthesis from Aggarwal,^20,21^ Crudden,^22^ Burke^23^ and others,^24^ which have traditionally used pre-existing functional groups to direct iterative synthesis.

Our selection of the C2–C7′ linkage reflects the preference of DHI to couple through C2 upon oxidation, and thus, the likely prevalence of this linkage in natural materials.^10*d*,*e*,25,26^ We show that the resulting oligomers have a propensity for planarization in the excited state, and that they exhibit pronounced vibronic structure that is particularly prominent in emission. Their spectra further show trends with size that parallel ones seen for conjugated oligomers, such as *para*-phenylene vinylene (PPV),^27^ although with considerably shorter effective conjugation lengths of just four DHI units. Poor orbital coupling between the π-systems of adjoined DHI units disfavors conjugation, helping to explain why visible absorption is unlikely to emerge from even very long chains of DHI subunits.

## Results and discussion

**Scheme 1:**
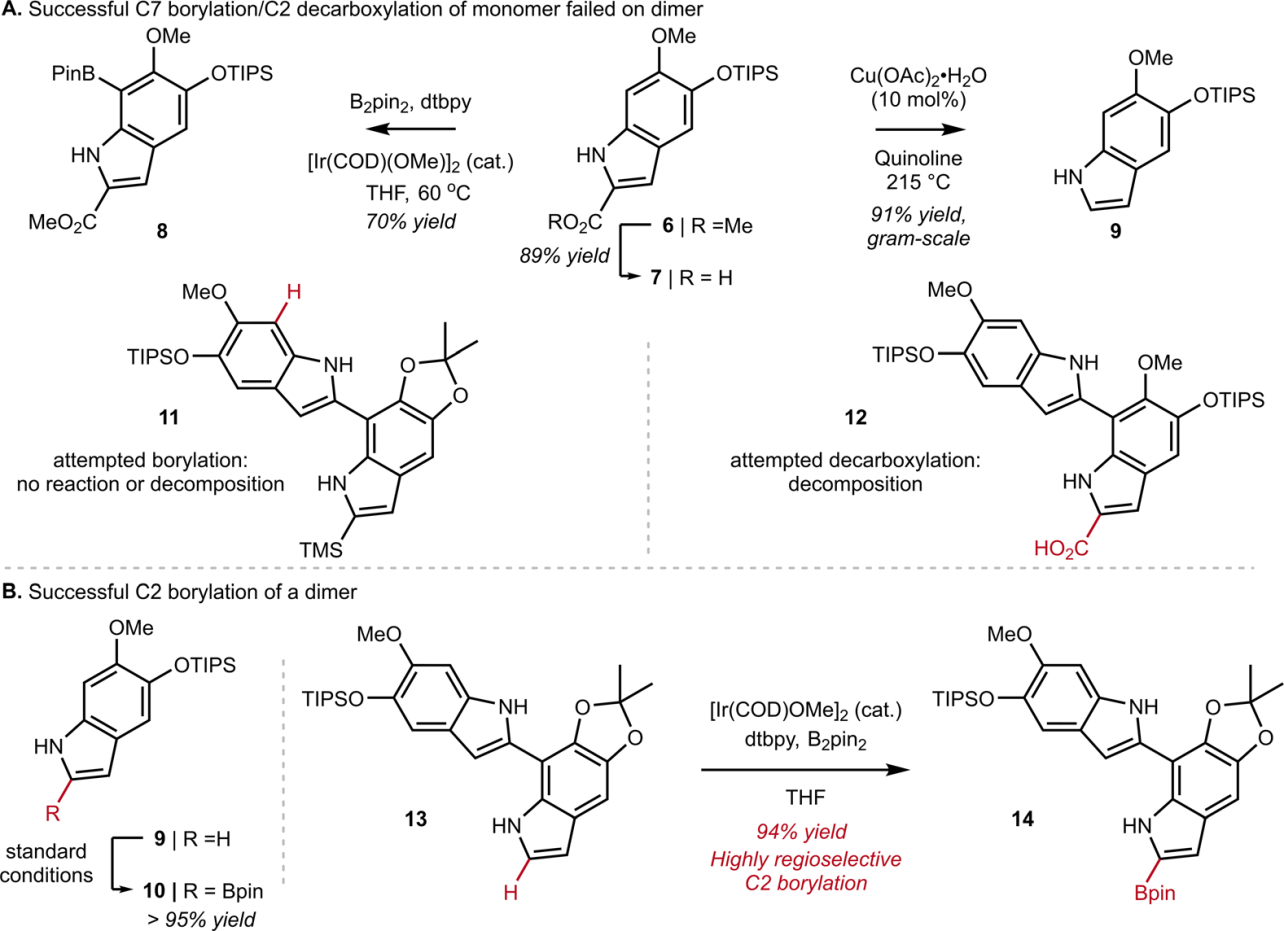
(A) Successful C7 borylation on monomer 6 or decarboxylation on monomer 7 failed on dimer 11 or 12. (B) Successful C2 borylation of monomer 9 can be extended to dimer 13*via* Ir-catalyzed C2-borylation.

At the outset of our work, we investigated the chain-extension of a C2, C7′-dimer of DHI (Scheme 1A), but discovered several challenges that were not evident at the monomer stage. While we had success in using N–H directed Ir-catalyzed C–H borylation to functionalize C7 of monomer 6 to provide 8, we observed little reactivity and no evidence of borylation at C7′ when applying these conditions to dimer 11. Likewise, attempts to decarboxylate at C2 worked well on monomer 7 to provide 9, but once again failed on dimer 12. Ultimately, we discovered that the Ir-catalyzed borylation at C2 performed equally well on dimer 13 as it did on monomer 9 (see 9 to 10 in Scheme 1B inset), provided that we pre-activate the catalyst prior to the addition of substrate. Thus, pre-mixing [Ir(OMe)(COD)]_2_ (3.0 mol%), 4,4-di-*tert*-butyl-2,2′-dipyridyl (dtbpy) (6.0 mol%) and B_2_pin_2_ (1.0 equiv.) in dry THF for 30 min at ambient temperature under an inert atmosphere afforded a catalyst mixture to which we added 13 as a solution in THF. Heating to 60 °C for 1 h then led to C2-Bpin dimer 14 in 94% yield. To achieve high yields, it was important to carefully control reaction time and the number of equivalents of B_2_pin_2_, as extended times or excess B_2_pin_2_ resulted in over-borylation. These optimized conditions were adopted for all subsequent C–H borylation reactions.

**Scheme 2:**
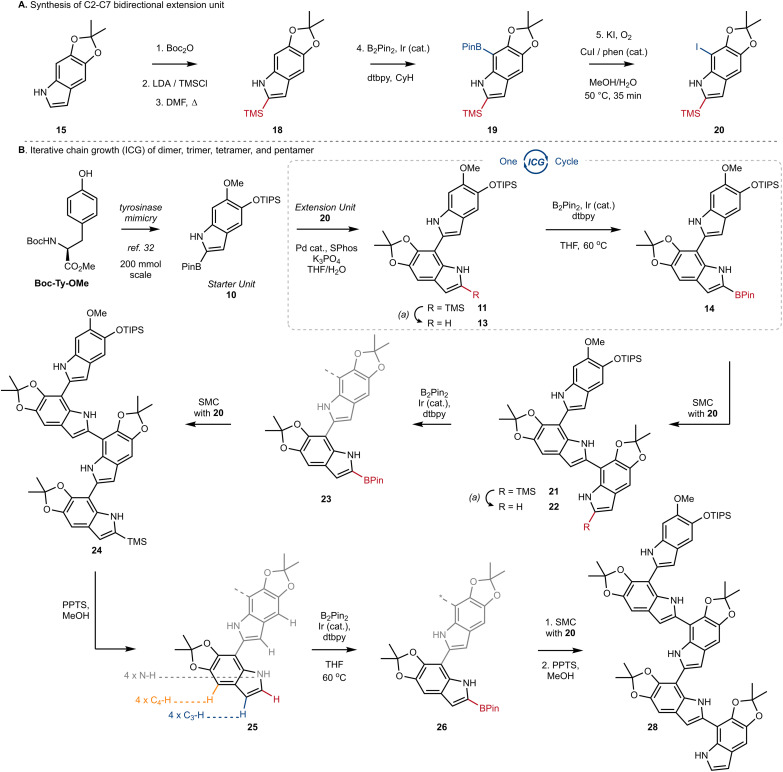
(A) Synthesis of C2–C7 bidirectional extension unit; Boc_2_O: di-*tert*-butyl dicarbonate, LDA: lithium diisopropylamide, TMSCl: trimethylsilyl chloride, DMF: dimethylformamide, dtbpy: 4,4-di-*tert*-butyl-2,2-dipyridyl, CyH: cyclohexane, Phen: 1,10-phenanthroline. (B) Iterative chain growth (ICG) of dimer, trimer, tetramer, and pentamer; Ir (cat.): [Ir(OMe)(COD)]_2_, Pd (cat.): Pd(OAc)_2_, PPTS: pyridinium *p*-toluenesulfonate, B_2_pin_2_: bis(pinacolato)diboron, SMC: Suzuki–Miyaura coupling.

Next, we turned our attention to the design and synthesis of a 2,7-functionalized DHI-extension unit, which could extend a chain by Suzuki–Miyaura Coupling (SMC) (Scheme 2A). We had two criteria for this partner that led us to prepare tetra-substituted DHI (20), possessing an iodide at C7 suitable for oxidative addition, and a trimethylsilyl (TMS) group at C2, suitable for blocking unintended reactivity at C2 or C3 (Scheme 2A). To prepare 20, we turned to the conditions of Smith^28*a*^ and Snieckus^28*b*^ to install a TMS group *via* directed *ortho* metalation of Boc-protected monomer 15, before removal of the Boc-group and N–H directed chemoselective borylation at C7. This multi-step protocol proved to be necessary, as efforts to install the TMS group by a direct C–H silylation of 15 using the conditions of Falck^29^ and Takai^30^ were less effective and provided 18 in only 10% yield. Finally, we could convert 19 into 20 using Hartwig's conditions for catalytic aerobic iodination, consisting of CuI, 1,10-phenanthroline (phen), and potassium iodide,^31^ which demonstrated good levels of chemoselectivity for oxidation of the Bpin in the presence of the electron rich N–H indole. Using this sequence of reactions, we could reliably prepare multiple grams of 20 starting from 15.

With a strategy for chain elongation and an extension unit in hand, we were now positioned to investigate an ICG that could provide 2,7′-linked indoles (Scheme 2B). We have previously reported a gram-scale synthesis of starter unit 10 beginning from Boc-Ty-OMe.^32^ Incorporation of the TIPS ether in 10 was strategic, as it helped with solubility and blocked any inadvertent functionalization at C4 in downstream reactions. Thus, SMC of starter unit 10 with extension unit 20 proceeded smoothly to afford dimer 11 in 82% yield, using our optimized conditions consisting of Pd(OAc)_2_ (5 mol%), SPhos (5.5 mol%), K_3_PO_4_ (2.1 equiv.) and a mixture of THF and H_2_O (3 : 1, 0.1 M). We highlight the effectiveness of these conditions, given the well-known challenges of SMC reactions involving free N–H groups and 2-heteroaryl boronic esters.^33^

The key sequence for chain extension began by chemoselective removal of the C2′ TMS group in the presence of the TIPS-ether and the acetonide using pyridinium *p*-toluenesulfonate (PPTS) in MeOH, to provide 13 in 97% yield. With the C2′ position liberated, we employed our optimized conditions for Ir-catalyzed borylation to produce 14 in almost quantitative yield, setting the stage for another cycle of ICG. We were pleased to see the effectiveness of SMC between extension unit 20 and dimer 14, which afforded trimer 21 in 86% yield under our standard conditions. Iteration could then once again be initiated by desilylation of 21 using PPTS/MeOH, setting the stage for borylation at C2′′. The selectivity of this process was impressive, as it afforded borylated trimer 23 as a single regioisomer in a 70% yield over two steps. Two additional rounds of ICG were then conducted under our standard protocol, affording tetramer 25 and finally pentamer 28 in good yields over the standard sequence (Scheme 2B). We did notice that borylation of tetramer 25 was slower than the borylation of dimer 13 or trimer 22. And likewise, SMC using trimer 23 or tetramer 26 required elevated temperatures, under which we began to observe competitive protodeborylation. Nevertheless, we highlight the selectivity in the borylation of tetramer 25, which must retain preference for C2–H in the presence of 9 other sp^2^-hybridized C–H bonds and 4 N–H bonds. In each case of iteration, our process affords tens to hundreds of milligrams of sample with high purity, setting the stage for the detailed analytical studies described below.

With monomers 9 and 15 as well as the oligomers 13, 22, 25, and 28 in hand, we studied how their optical properties evolve with size by steady-state absorption and emission spectroscopy. Because the precise microenvironment of melanin granules remains unknown, we selected acetonitrile and cyclohexane in order to gauge the effects of solvent polarity. The resulting homogeneous solutions were robust and no changes in spectra were noted for samples left standing in air for several days.

**Fig. 3:**
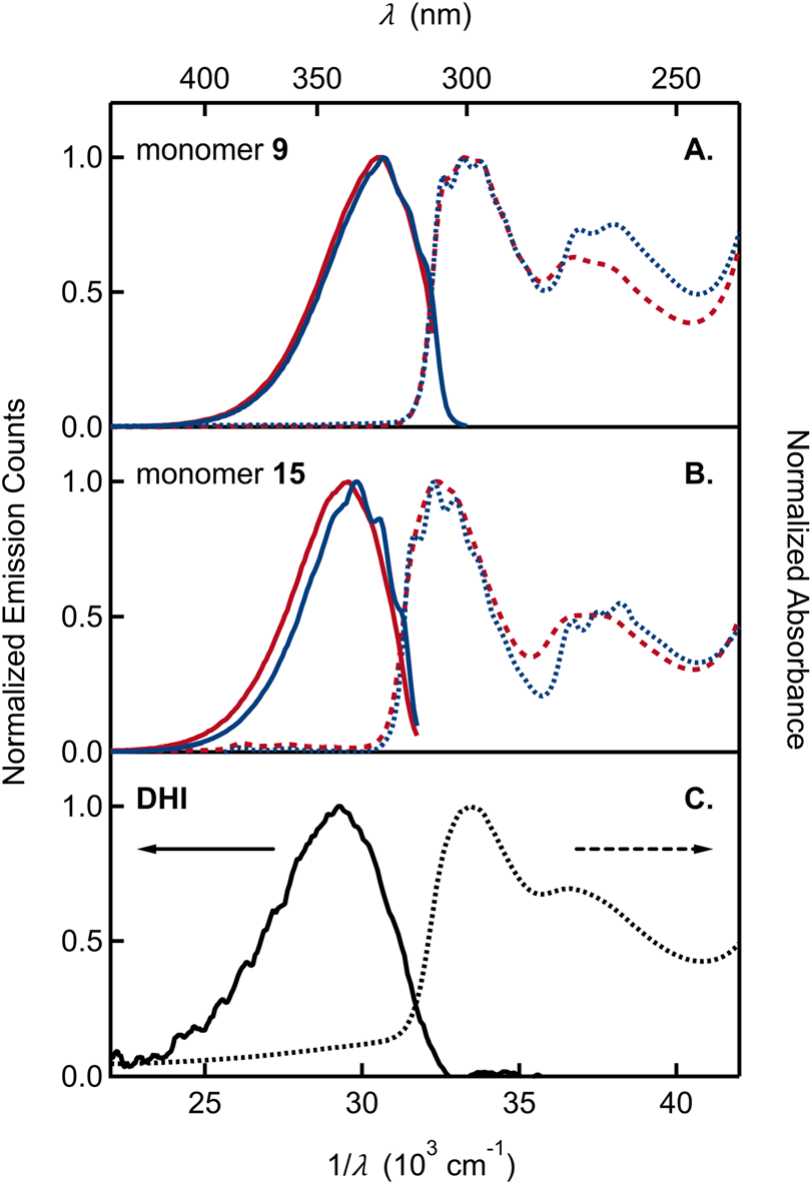
Absorption and emission spectra plotted *vs.* wavelength (*λ*) and wavenumber (1/*λ*) of (A) monomer 9 and (B) monomer 15 in acetonitrile (red curves) and cyclohexane (blue curves). (C) Absorption and emission spectra of DHI in aqueous solution (black curves). Absorption and emission spectra are shown by dashed and solid curves, respectively.

The UV-vis absorption spectra of monomer 9, bearing the OMe/OTIPS substitution, and 15, bearing the acetonide (Fig. 3A and B), feature two broad bands separated by ∼4000 cm^−1^. A longest wavelength band near 300 nm is accompanied by a second band near 270 nm that is approximately half as intense as the first. The position of each band is nearly unchanged in cyclohexane *vs.* acetonitrile solutions. The longest wavelength band of monomer 9 is shifted to a slightly longer wavelength compared to monomer 15, although the higher energy band occurs in the same spectral region for both compounds. Peak positions in the emission and absorption spectra are tabulated in Tables S1 and S2,† respectively.

By way of comparison, the UV-vis spectrum of a commercial sample of DHI measured in aqueous solution (Fig. 3C) shows a similar two-band pattern, which is also seen in other DHI derivatives such as 5,6-dihydroxytryptamine (DHT)^34^ and *N*-methyl-5-hydroxy-6-methoxyindole (MHMI).^35^ However, unprotected DHI is difficult to study because of its susceptibility to oxidation.^36^ The DHI sample we obtained from a commercial supplier arrived as an almost black powder, suggesting the presence of eumelanin-like contaminants that give rise to the broad ‘tail’ that begins at 320 nm and extends through the visible spectrum (Fig. 3C). Previously published absorption spectra of DHI show similar red tailing,^37^ which increases over time when DHI is left standing in aqueous buffer solution and accelerates with UV irradiation (see Fig. S2† in ref. 37a). In contrast, protected monomers 9 and 15 do not absorb at visible wavelengths and have a steeply rising edge or onset to the longest wavelength absorption band in the UV. Spectra like those in Fig. 3 were measured on fresh and aged solutions (Fig. S1†). Their constancy indicates that melanin forming reactions are completely inhibited when the OH groups of DHI are protected, even though the carbon atoms where C–C coupling has been shown to occur (*i.e.*, C2, C3, C4, and C7) are left unprotected.^15^ This supports the prevailing hypothesis that H atom loss from the catechol is an important mode of reactivity for producing the semiquinone and quinone forms of DHI needed for chain growth under biosynthetic or biomimetic conditions.

The absorption and emission spectra of the monomers show vibronic structure, which is better resolved in cyclohexane, but is still seen clearly in acetonitrile solution. The vibronic structure is slightly more resolved in absorption than in emission. The longest wavelength absorption band of each monomer in cyclohexane has three peaks or subbands and two shoulders at higher frequencies. These vibronic features define a progression with a spacing of approximately 700 cm^−1^ (Table S1†). In contrast, no vibronic structure is seen in the absorption spectrum of commercial samples of DHI in water (Fig. 3C).

Monomers 9 and 15 have strong photoluminescence. We measured quantum yields of ∼35% in acetonitrile (Table 1), which are similar to the reported quantum yield of 33% for MHMI in the same solvent.^35^ The emission quantum yield of DHI is likely lower, although a value has never been reported. Notably, Sundström and co-workers attributed the considerable fluorescence of DHI-samples to contaminating dimers formed through oxidation reactions,^37*a*^ but the true source of fluorescence has not been confirmed.

**Table 1:** Fluorescence quantum yields, *Φ*_f_ (%), of the monomers and oligomers in acetonitrile. Experimental details are in ESI

	9	15	13	22	25	28	DHI^a^
*Φ*_f_	35 ± 3	33 ± 3	49 ± 1	46 ± 2	42 ± 2	37 ± 2	<1

a Solvent: water.

**Fig. 4:**
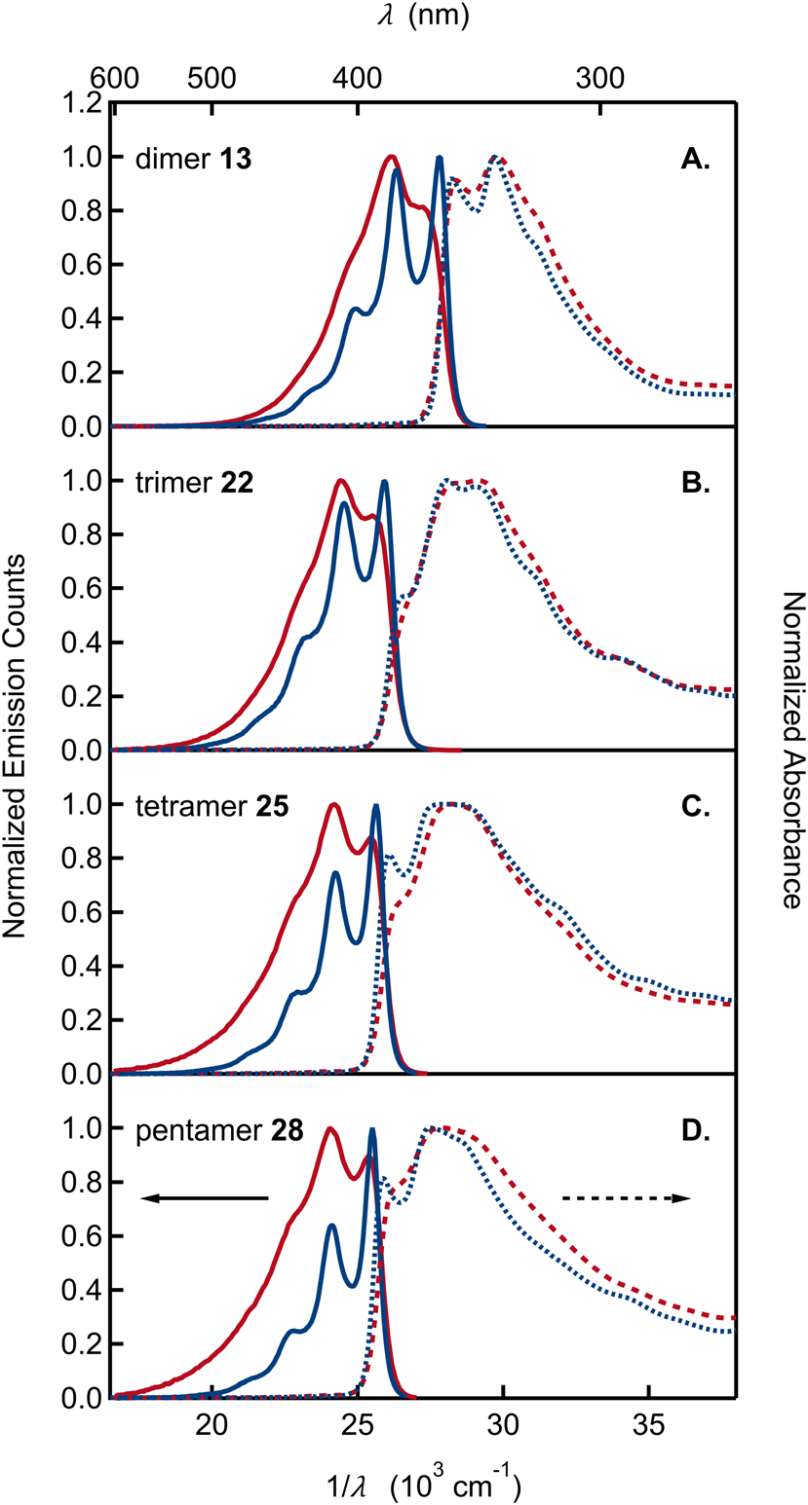
Absorption and emission spectra, shown by dashed and solid curves, respectively, plotted *vs.* wavelength (*λ*) and wavenumber (1/*λ*) in acetonitrile (red curves) and cyclohexane (blue curves) of (A) dimer 13, (B) trimer 22, (C) tetramer 25, and (D) pentamer 28.

Absorption and emission spectra of the remaining dimer to pentamer model compounds (13, 22, 25, and 28) are compared in Fig. 4. Like the monomers, the oligomers have sharp absorption onsets, contrasting with previously published absorption spectra of various DHI oligomers that show extensive red tailing.^14,37*a*,38^ As reviewed by d'Ischia and co-workers, oligomers of DHI are typically prepared by adding an oxidant to DHI, stopping the reaction at an early stage with a reductant, and, finally, isolating the products as O-acetylated compounds.^8*d*^ It is important to note that these reaction conditions also give rise to synthetic melanins, creating a complex mixture of products from which it is difficult to isolate a dimer of interest in pure form. In our opinion, the red tailing that extends to visible wavelengths in the absorption spectra of these previous samples is a reliable sign that contaminating chromophores are present. The oligomers synthesized here using ICG are stable, allowing precise spectroscopic measurements for the first time. Their absorption spectra neither change with time nor are they altered significantly after extended UV irradiation (Fig. S2†).

**Fig. 5:**
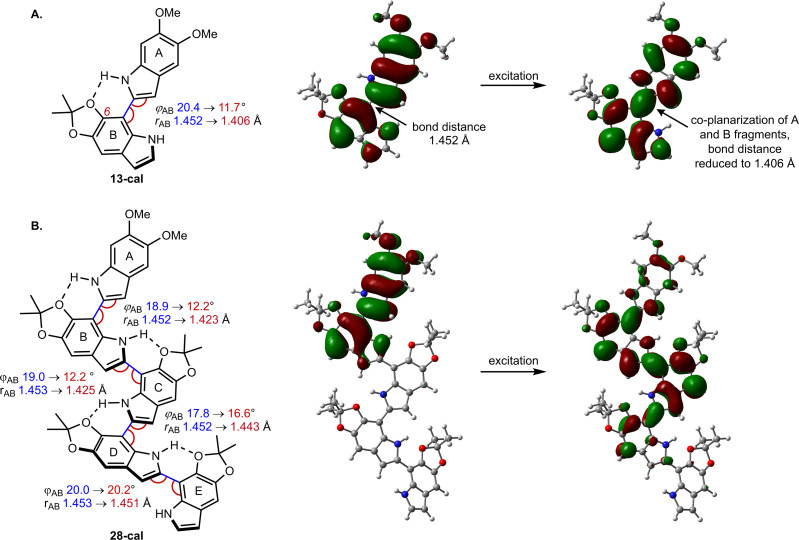
Geometric changes induced by excitation and natural transition orbitals in (A) dimer 13-cal and (B) pentamer 28-cal. The drawings display the labelling scheme for the oligomer fragments and the distance and rotational (dihedral) angle between fragments; parameters at the ground and excited state are shown in blue and red, respectively. The orbitals were obtained at the S_1_ minimum geometry and describe the S_1_ excitation in terms of an electron being excited from a hole to a particle orbital (isodensity value 0.02).

The spectra were modeled computationally with time-dependent density functional theory (TD-DFT), using the PBE0 functional and the 6-311g(d,p) basis set. To save computational effort, the TIPSO group in the calculations was replaced by a MeO group, which should not lead to large changes in the photophysics. We use the conformations depicted in Fig. 5, with intramolecular hydrogen bonds between the N–H group of one DHI unit and the C6–O of its neighbor. According to our calculations for dimer 13-cal, the computational model of 13, this structure is favored by 1.5 kcal mol^−1^ with respect to the rotational isomer that lacks the H bond (not shown). The result is a population of the two rotational isomers in a ratio of approximately 9 : 1. Calculated absorption and emission energies reproduce the experimental trends from dimer to pentamer with generally good accuracy (Table S5†). The differences between calculated 0-0 energies of the oligomers and the highest-energy subband in the emission spectra (Table S1†) in cyclohexane are a few nm, while the calculated emission wavelength is slightly overestimated by 8–16 nm in acetonitrile.

The absorption spectra of oligomers 13, 22, 25, and 28 cannot be modeled by linear combinations of the absorption spectra of the constituent monomers. For dimer 13 and trimer 22, the long wavelength absorption band is shifted to longer wavelengths and is roughly twice as intense as would be predicted by summing the molar absorption coefficients of the constituent monomers (Fig. S3†). These differences indicate electronic coupling among the DHI subunits. Additional experimental evidence for electronic coupling comes from the pronounced vibronic structure seen in the absorption and, especially, emission spectra. In both solvents, the emission spectra of the dimer through pentamer are more highly resolved than the monomer spectra. The spacing between emission subbands is ∼1400 cm^−1^ (Table S1†). The spacing is extremely similar for dimer to pentamer as can be seen when the spectra are shifted in frequency (Fig. S4†).

To the best of our knowledge, vibronic structure in the spectra of DHI- or DHI-like oligomers has not been discussed previously. Nogueira *et al.* briefly mentioned vibronic structure in their absorption spectrum of the DHI derivative 5-methoxy-6-hydroxyindole in water.^37*a*^ However, they did not discuss vibronic structure in their DHI dimers, even though vibronic features are evident in the emission spectrum of the 2,2′ dimer of DHI (see Fig. S4† in ref. 37a; similar vibronic structure is evident in an earlier absorption spectrum of the same compound^38*a*^). There is little evidence of vibronic structure in published spectra of 2,7′ DHI dimers in aqueous solution.^37*a*,38*b*^ In our view, this reflects the greater inhomogeneous broadening in water *vs.* acetonitrile as well as the possible presence of impurities, which broaden the spectra.

We highlight several additional trends in our spectra: (1) unlike the monomers, the envelope of the lowest energy band is narrower in emission than in absorption (the reverse trend is seen in the monomers); (2) relative to the highest energy subband of the emission spectrum (the 0-0 band), the amplitudes of later subbands in the series decay more rapidly with increasing oligomer size (Table S3†). This trend causes the emission spectrum to narrow for the larger oligomers; (3) the fluorescence quantum yields for dimer through pentamer vary from 40% to 50% and are slightly higher than for the monomers (Table 1).

These features, along with the strong vibronic progression seen in the emission spectra (Fig. 4), closely parallel the behavior of other well-studied conjugated materials, such as PPV. Notably, a spacing of 1400 cm^−1^ to 1500 cm^−1^ between the 0-0 and 0-1 transitions seen for the DHI-oligomers in Fig. 4 is similar to the spacing observed for PPV, and more generally, is a common feature of conjugated materials in which π-systems are linked by C–C single bonds with relatively free rotation.^27^ Likewise, the absence of mirror symmetry seen in our emission and absorption spectra is also observed in conjugated hydrocarbons, which have low torsional barriers to rotation about adjoining C–C single bonds.^39^ Calculations on dimer 13-cal show two ground-state vibrational modes involving the C2–C7′ inter-unit bond with other C–C and C–N bonds in the rings, with scaled frequencies of 1529 and 1607 cm^−1^. These modes and their analogues in the larger oligomers are likely responsible for the observed vibrational spacing seen in our emission spectra.

The different vibronic structure of the absorption and emission spectra can be related to differences in the ground and excited state potential energy profiles for rotation about the C2–C7′ bond (see the calculated profiles for dimer 13-cal in Fig. S5†). For the hydrogen-bonded conformations, there are two atropisomers in the ground state with dihedral angles of 20° and −20° around the bond. These isomers can interconvert by a small rotation and are separated by a barrier of 0.9 kcal mol^−1^, giving rise to a double-well potential. Planarization in the excited state decreases these angles to 10° and −10°, and the barrier is reduced to 0.2 kcal mol^−1^, giving an almost flat potential (Fig. 5). While a more extensive study is necessary to thoroughly elucidate this phenomenon, we hypothesize that the different potential shape along the rotational coordinate for the two states may cause the emission spectrum to be more highly resolved than the absorption spectrum. A similar argument was given to explain the absence of symmetry between absorption and emission in *para*-terphenyl.^40^

The ratio of the 0-1 to 0-0 emission subband intensity decreases with increasing chain length (Table S3†), matching the trend in PPV oligomers. In the latter systems, the Huang-Rhys factor, which is approximately equal to this ratio, decreases with increasing chain length.^27,39,41^ This behavior is seen because the geometry change upon electronic excitation decreases for longer chains due to their increased excited state delocalization.^41*b*^ The emission spectra become narrower with *n*, but the absorption spectra become broader with increasing *n* due to increased conformational disorder. Previously, d'Ischia and co-workers reported broadening with size in the long wavelength absorption band of O-acetylated DHI oligomers.^38*a*^

A further trend shared with conjugated oligomers is the systematic shift of transition energies to longer wavelengths with increasing size.^27,39,41*b*^ Our solution concentrations (∼10 μM) are too low to explain this shift by aggregation. Aggregates of PPV oligomers display a blue shift in the absorption maximum and red shift in the absorption onset consistent with H-aggregate formation.^27^ In addition, their emission is quenched by at least one order of magnitude. The absence of strong fluorescence quenching (Table 1) and our dilute solution conditions allow us to rule out aggregate formation. Instead, we assign the progressive red shift of the 0-0 energy to extended conjugation that increases with chain length. Transition energies of conjugated oligomers often increase linearly with the inverse of the number of residues, *n*.^42^ This scaling, which can be anticipated from the inverse length dependence of the energy levels of a particle in a one-dimensional box, does not continue indefinitely due to saturation.

**Fig. 6:**
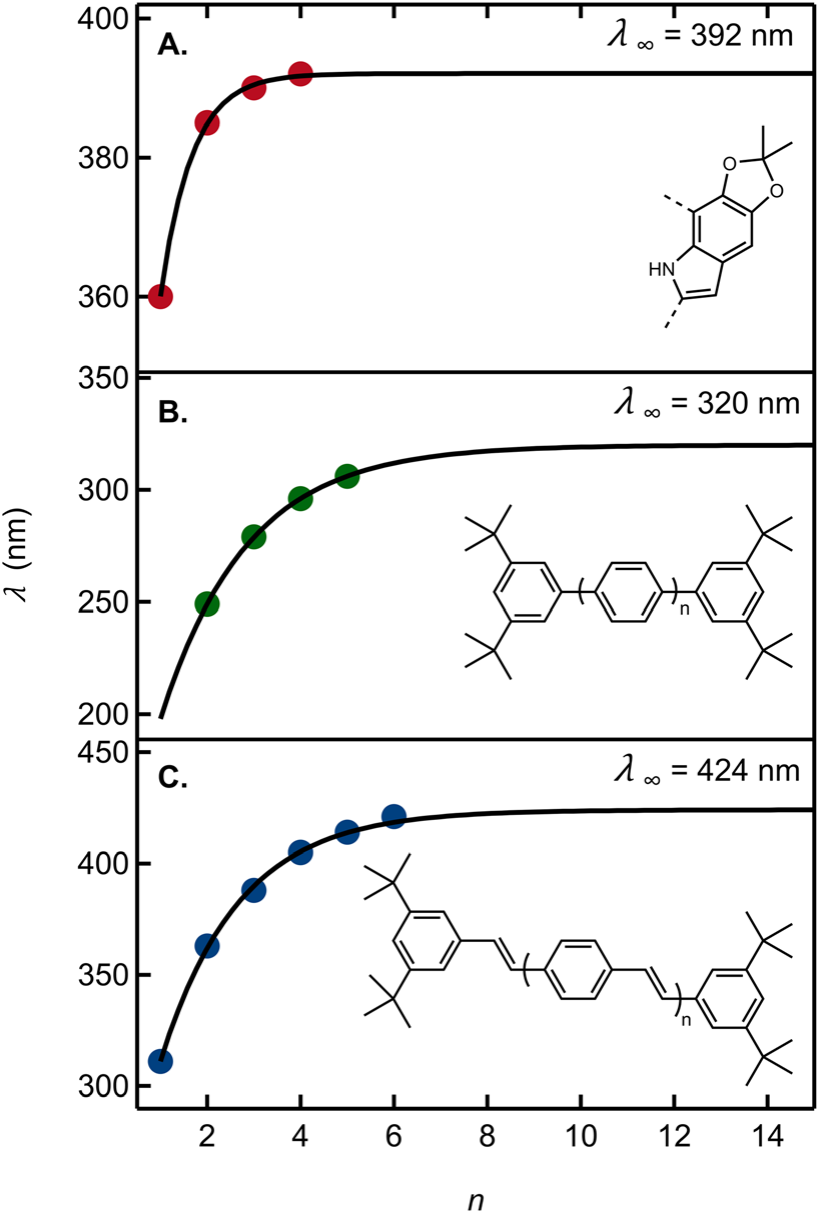
(A) Wavelength of the 0-0 vibronic emission peak maximum as an estimate of the electronic origin (red circles) *vs.* the number of acetonide-protected monomers, *n* (Table S3†). The solid curve is a fit to the model in ref. 42. Absorption peak maxima *vs.* the number of repeating units for (B) oligo(1,4-phenylene) in THF and (C) oligo(aryleneethenylene) in THF. Data in panels B and C are from ref. 42. For each graph, the asymptotic estimate of the absorption onset for the infinitely long polymer (*λ*_∞_) is shown.

To this end, we fit a model due to Meier *et al.*^42^ to a graph of the 0-0 emission band maxima of our oligomers in cyclohexane *vs. n* (Fig. 6A). Here, *n* stands for the number of acetonide-protected DHI units (*i.e.*, *n* = 1 for 13, 2 for 22, *etc.*). The fit parameters predict an effective conjugation length of *n* = 3, *i.e.* a total of four DHI units (see ESI Section 5†). Results from ref. 42 showing the effective conjugation length of an oligophenylene and an oligophenylvinylene are provided in Fig. 6B and C for comparison.

Using the criterion proposed by Meier *et al.*,^42^ the fit predicts that an infinitely long acetonide-protected DHI polymer with 2,7′-connectivity would have an asymptotic 0-0 transition energy of 392 nm. Absorption by a long polymer would thus not extend to visible wavelengths and would moreover hardly differ from an oligomer containing approximately three acetonide-protected DHI units. This is consistent with a computational result showing no visible absorption by DHI dimers.^14^ Tuna *et al.*^43^ computed the absorption onset of DHI oligomers for dimers through pentamers averaged over all possible isomers. Their prediction that absorption by reduced oligomers might plateau just below the visible spectral region is fully validated by our experimental findings.

Computationally, the extent of excitation delocalization along the oligomer chain can be tracked by analyzing the geometric changes induced by excitation and the natural transition orbitals (NTO), which give a compact description of the excitation in terms of a hole orbital and a particle orbital (see Fig. 5).^44^ We focus on characterizing the excited states of dimer 13-cal and pentamer 28-cal as the limiting cases of our set. Each indolic subunit within the oligomer is labelled with a letter A to E, where the first fragment A is the lone, non-acetonide protected subunit. In the dimer (13-cal, Fig. 5A), the excitation is delocalized over the two fragments. This induces a co-planarization of the fragments, where the rotational angle *φ* is reduced from 20.4° to 11.7° and the interfragment distance is reduced from 1.45 Å to 1.41 Å. This is consistent with the nature of the NTO along the C2–C7′ bond, which is highlighted in Fig. 5A. It is antibonding in the hole orbital and bonding in the particle orbital. Therefore, excitation of an electron from the hole to the particle has the effect of increasing the double bond character of the C2–C7′ bond, which results in co-planarization.

A similar analysis for the pentamer 28-cal shows that excitation is not delocalized along the whole molecule, but only along the first four fragments (Fig. 5B), which is consistent with the fit to the Meier model. We call this configuration Ex1, which stands for exciton 1. In the NTO, the hole orbital is localized on fragments A and B, and the particle orbital on fragments A–D, with no significant participation of E. The Ex1 configuration has a significant charge transfer character (see the dipole moments of the ground and excited states in Tables S7 and S8†). Consistent with the orbital extension, the excitation induces significant co-planarization of rings A, B and C, as shown by the changes in the interfragment distances and rotational angles. However, the changes in co-planarity of rings C and D are much less pronounced, and the distance and rotational angles between D and E stay almost unchanged, confirming the limited character of the excitation.

The picture of an exciton constrained to just four fragments is further supported by the fact that it is possible to locate minima where the excitation is localized on different fragments. They correspond to different electronic states on the S_1_ potential energy surface of the pentamer. The S_1_ minimum with the Ex1 configuration has the hole orbital centered on rings A and B, but we have located two further S_1_ minima where the hole orbital is localized on rings B and C, and C and D (see Fig. S7†). They are called Ex2 and Ex3, respectively. The corresponding minima have different geometries, with the highest degree of co-planarity found between rings B and C, and C and D, respectively. From the energy point of view, they lie 0.10 and 0.14 eV higher in energy than Ex1, so they may not be relevant to the photophysics because configuration Ex1 is more stable, and emission will take place preferentially from the most stable minimum. However, the existence of different minima on the S_1_ surface is a direct consequence of the limited extension of the excitation, which is not spread along the whole pentamer chain and allows the excitation to adopt different electronic configurations.

Analysis of the excitation in trimer 22-cal and tetramer 25-cal confirms the trends described for 13-cal and 28-cal (see Tables S7 and S8†). In the trimer, an increase in co-planarity upon excitation is found between fragments A and B, and between B and C. In the tetramer, the increase in co-planarity is also seen between fragments A, B and C, but is less pronounced between C and D due to the limited delocalization of the exciton. The existence of different minima corresponding to different exciton configurations is also found for the tetramer.

**Fig. 7:**
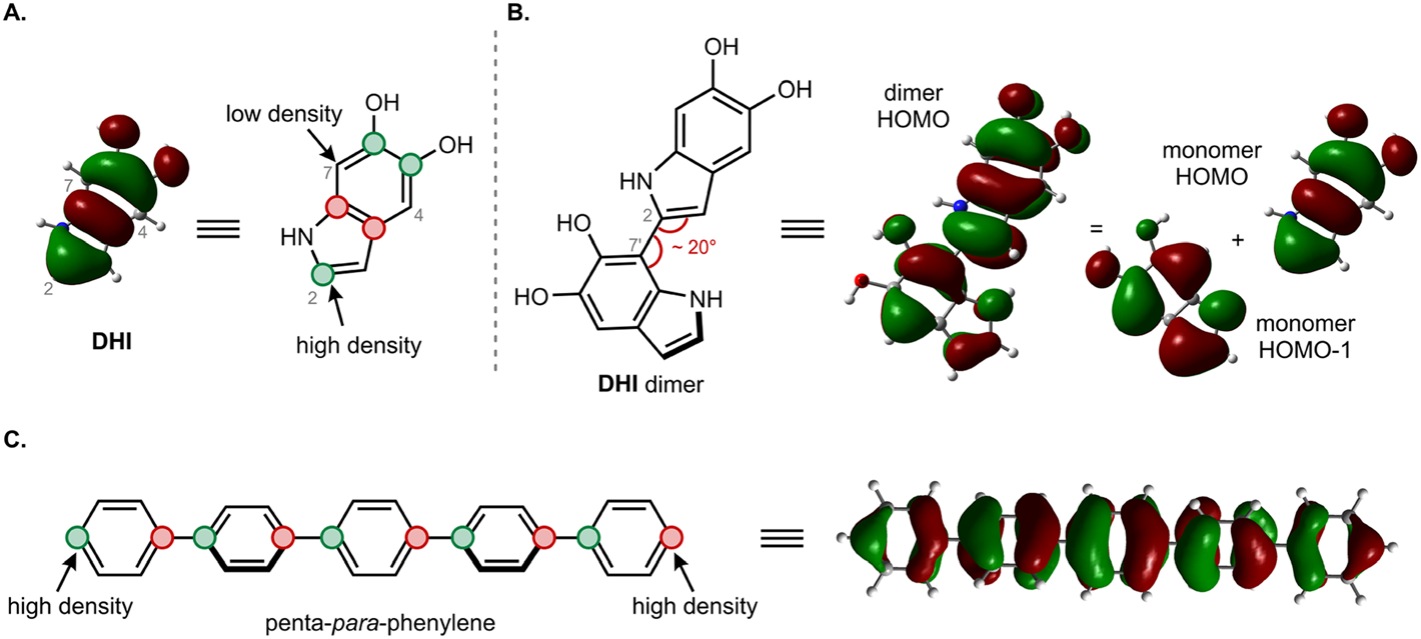
Comparison of orbital coupling in DHI and *para*-phenylene oligomers. (A) HOMO of DHI monomer. (B) HOMO of DHI dimer resulting from combination of the HOMO and the HOMO−1 of the two fragments. (C) HOMO of penta-*para*-phenylene resulting from combination of one of the benzene HOMOs.

The moderate degree of delocalization found in the DHI oligomers can be explained on the basis of the orbital coupling between the indolic fragments. Excitation delocalization results from efficient electronic coupling between the fragments, which is favored by the co-planarity between them, and by a favorable orbital nodal structure. Interestingly, delocalization in DHI oligomers is less favorable than in oligo(1,4-phenylene), even though the DHI rings are more co-planar than the phenyl rings in oligo(1,4-phenylene), with rotational angles lying around 20° and 40°, respectively. Hence, the poor electronic coupling in the DHI oligomers is the result of the nodal structure of the fragment orbitals, as shown in Fig. 7. The HOMO of DHI (Fig. 7A) has high density at C2 but low density, almost a node, at C4 and C7. The poor electronic coupling along the C2–C7′ bond therefore arises from the low orbital density of the HOMO at C7. In fact, inspection of the hole orbitals of the oligomers in Fig. 7 shows that they are the result of coupling the HOMO of fragment A with the HOMO−1 of fragment B. The coupling of orbitals of different energy is unfavorable for delocalization, and as a result the hole orbitals of the oligomers are restricted to two rings, as shown for the pentamer in Fig. 5 and S7.† In contrast, in *para*-phenylene the fragment orbital that originates from the HOMO of the oligomer is one of the HOMOs of benzene with high density on the connecting carbon atoms, which allows for efficient delocalization along the chain, as shown for the HOMO of penta-*para*-phenylene in Fig. 7C. Generalizing our findings for the C2–C7′ connected oligomers and considering that the HOMO of DHI has low density at C4 and C7, we hypothesize that poor electronic coupling will be common for DHI oligomers involving connectivity at positions 4 and 7. Orbital nodal structure favors electronic coupling in oligomers with a 2,3′ bonding pattern, but coupling between these positions is reduced by negative steric interactions and loss of co-planarity. These aspects lead us to speculate that low effective exciton length is a common feature of reduced DHI oligomers, in line with the conclusions from d'Ischia.^38*a*^

Finally, the experimental evidence presented here shows that excited states of DHI oligomers exhibit many of the spectroscopic characteristics of excited states in conjugated hydrocarbon oligomers and polymers. Although the excitations in the DHI oligomers are significantly more localized, they still extend over several residues. Nevertheless, our results provide direct spectroscopic evidence that fully reduced oligomers of DHI, regardless of length, cannot absorb visible or NIR light.

## Conclusions

In summary, we have described a method to prepare oligomers of DHI derivatives by an ICG that integrates SMC with site-selective C–H borylation. The high performance of these metal-catalyzed steps on such heteroatom-rich, polysubstituted indoles is noteworthy, and provides a blueprint for synthesizing increasingly complex eumelanin model compounds. To this end, we see opportunities to merge the ICG reported here with our previous studies on blocked subunits^15^ in order to investigate the effects of oxidation on property emergence. From the present work, we see similarities between the electronic properties of the DHI backbone and other conjugated oligomers, such as PPV. However, the shape of the HOMO orbital in DHI is unfavorable for efficient orbital coupling between the C2–C7′ connected fragments. As a result, the effective conjugation length lies between 3 and 4 indolic subunits, which results in only moderate red shifts to absorption and emission with increasing chain length, and DHI subunits alone cannot account for eumelanin's optical properties. Moving forward, we see a clear need to investigate increasingly complex model compounds with mixed oxidation states and see an important role for site-selective C–H functionalization to enable their synthesis. Indeed, C–H functionalization could broaden the scope of currently employed ICGs, and even provide complementary substitution patterns to what is currently accessible from bidirectional building blocks.

## Data availability

The data supporting this article have been included as part of the ESI.†

## Author contributions

Conceptualization: HH, JPL, BK and LB; funding acquisition: JPL, BK and LB; investigation: HH, LK, AM, YZ, MAB; supervision: JPL, BK, LB; writing: HH, LK, JPL, BK, LB.

## Conflicts of interest

There are no conflicts to declare.

## Supplementary Material

SC-016-D4SC08610D-s001
