# Supplementary material for: An iterative synthesis of poly-substituted indole oligomers reveals a short effective conjugation length in eumelanin model compounds
Source: Chem Sci. 2025 Feb 5;16(10):4537–48. doi: 10.1039/d4sc08610d (PMC11836627; doi:10.1039/d4sc08610d)
Supplement: SC-016-D4SC08610D-s001 [file SC-016-D4SC08610D-s001.pdf]

Supplementary Information (SI) for Chemical Science.
This journal is © The Royal Society of Chemistry 2025

# An Iterative Synthesis of Poly-Substituted Indole Oligomers Reveals a Short Effective Conjugation Length in Eumelanin Model Compounds

Haiyan Huang, [a] Lilia Kinziabulatova, [b] Anju Manickoth, [c] Yiming Zhang, [a] Marisa A. Barilla, [b] Lluís Blancafort,*[c] Bern Kohler,*[b] and Jean-Philip Lumb*[a]

a. Department of Chemistry, McGill University, 801 Sherbrooke Street West, Montreal, QC H3A 0B8, Canada

b. Department of Chemistry and Biochemistry, The Ohio State University, 100 W. 18th Avenue, Columbus, OH 43210 USA

c. Institut de Química Computacional and Departament de Química, Universitat de Girona, Girona 17003, Spain

# Supporting Information

## Table of Contents

1. Scheme S1: The Biosynthesis of Eumelanin ........ 2
2. Materials and Methodologies for Iterative Synthesis ........ 2
3. General Procedures ........ 3
   a) General Procedure A: Suzuki-Miyaura Coupling of Indoles ........ 3
   b) General Procedure B: Ir-catalyzed Borylation of Indoles ........ 3
   c) General Procedure C: Desilylation of Indoles ........ 4
4. Synthesis and Characterization of Compounds ........ 4
5. Spectroscopy ........ 19
6. Computational Details ........ 25
7. Reference: ........ 31
8. $^{1}H$ and $^{13}C$ NMR Spectra ........ 31

## 1. Scheme S1: The Biosynthesis of Eumelanin

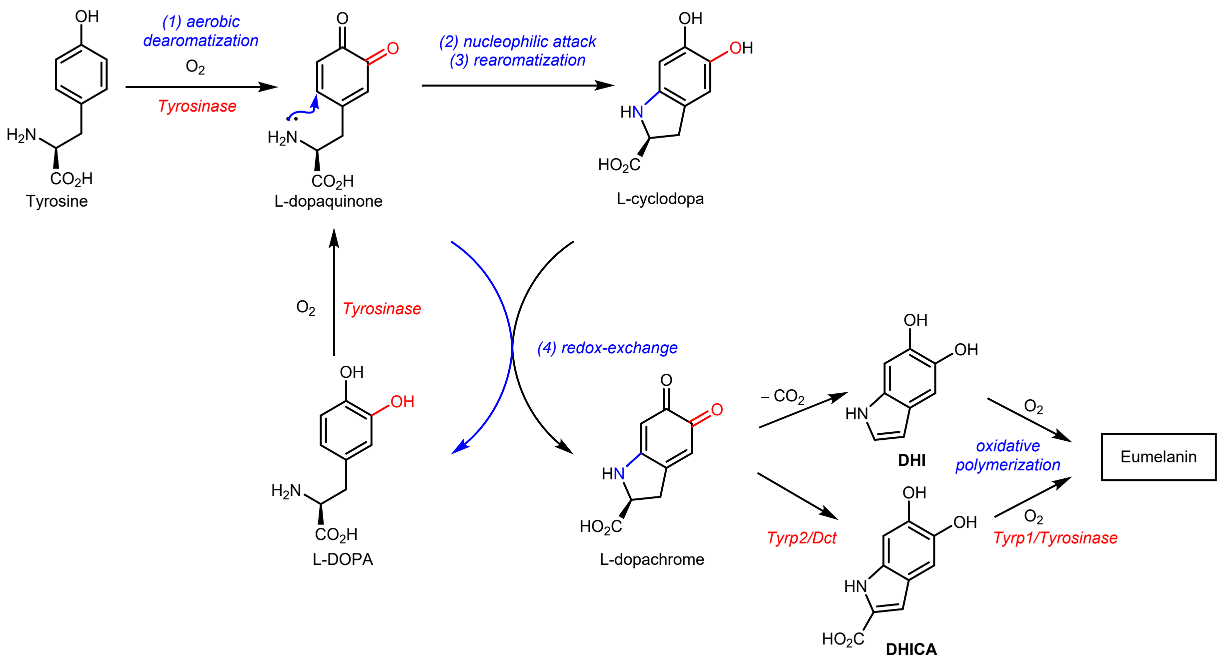


## 2. Materials and Methodologies for Iterative Synthesis

Chemicals and solvents were purchased from Sigma Aldrich, Alfa Aesar, Strem Chemicals, TCI or Oakwood Chemicals. Chemicals were used as received without further purification. THF was dried and purified using a PureSolv MD 7 (from Innovative Technology) or MB SPS 800 (from MBraun). Cyclohexane and quinoline were distilled over $CaH_2$ under $N_2$. $[Ir(COD)(OMe)]_2$ was purchased from Sigma Aldrich, and stored inside of a MBraun Labmaster glove box (<1 ppm $O_2$ and $H_2O$) filled with a dry $N_2$ atmosphere at –20 °C. Unless otherwise noted, reactions were performed in flame-dried glassware under a positive pressure of $N_2$ using standard synthetic organic, inert atmosphere techniques.

Proton nuclear magnetic resonance ($^1$H NMR) spectra were acquired using Varian Mercury 400 MHz, Varian Inova QANUC 500 MHz, Varian VNMRS 500 MHz, Bruker AVIIIHD 500 MHz, or Bruker AVIIIHD 400 MHz spectrometers. Chemical shifts (δ) are reported in parts per million (ppm) and are calibrated to the residual solvent peak. Coupling constants (*J*) are reported in Hz. Multiplicities are reported using the following abbreviations: *s* = singlet; *brs* = broad singlet; *d* = doublet; *t* = triplet; *q* = quartet; *m* = multiplet (range of multiplet is given). Carbon nuclear magnetic resonance ($^{13}$C NMR) spectra were acquired using Varian VNMRS 125 MHz, Bruker AVIIIHD 125 MHz, or Bruker

AVIIIHD 101 MHz spectrometers. Chemical shifts (δ) are reported in parts per million (ppm) and are calibrated to the residual solvent peak. $^{13}C$ chemical shifts are reported to one decimal place, unless two peaks are distinguishable in their second decimal place. In these cases, $^{13}C$ chemical shifts are reported to two decimal places. High resolution mass spectra (HRMS) were recorded using a Bruker maXis Impact TOF mass spectrometer. Fourier-transform infrared (FT-IR) spectra were recorded on an alpha Bruker FT-IR spectrometer. Analytical thin-layer chromatography was performed on pre-coated 250 mm layer thickness silica gel 60 $F_{254}$ plates (EMD Chemicals Inc.). Visualization was performed by ultraviolet light and/or by staining with potassium permanganate or cerium molybdate. Purifications by column chromatography were performed using either a Biotage Isolera™ One or standard column chromatography using silica gel (40-63 µm, 230-400 mesh).

## 3. General Procedures

### a) General Procedure A: Suzuki-Miyaura Coupling of Indoles

A flame-dried Schlenk tube equipped with a Teflon-coated stir bar and a glass stopper was charged with iodoindole (1.0 equiv), indole boronic acid pinacol ester (1.0-1.2 equiv), Pd-catalyst (5 mol%), ligand (5.5 mol%) and base (2.1 equiv). The Schlenk tube was evacuated and backfilled with $N_2$ (this process was repeated three times) prior to the addition of degassed dry solvent under a positive $N_2$ pressure, followed by addition of degassed distilled $H_2O$. The tube was sealed and heated to the indicated temperature in a pre-heated oil bath. After the indicated time, the reaction was cooled to ambient temperature atmosphere and quenched by the addition of saturated aqueous $NH_4Cl$ solution to pH=7. The phases were then separated, and the aqueous phase was extracted with EtOAc (3 × 20 mL). The combined organic fractions were then washed with brine then dried over $MgSO_4$, filtered, and concentrated *in vacuo*. The resulting residue was purified by flash chromatography on silica gel using the indicated solvent system as eluent to afford the desired product.

### b) General Procedure B: Ir-catalyzed Borylation of Indoles

In an inert atmosphere glove box, a flamed-dried vial equipped with a Teflon-coated stir bar was charged with indole (1.0 equiv) and dry, degassed solvent (THF, cyclohexane). In three separate flamed-dried vials bispinacolatodiboron ($B_2pin_2$, 1.0 – 2.0 equiv), 4,4'-

di-*tert*-butyl-2,2'-bipyridyl (dtbpy, 3.0 – 14.0 mol%), and $[Ir(COD)(OMe)]_2$ (1.5 – 7.0 mol%) were dissolved in dry, degassed solvent (THF, cyclohexanes). The solution of $B_2pin_2$ was added to the solution of $[Ir(COD)OMe]_2$ followed by the addition of dtbpy. The mixture was stirred in the glovebox for 0.5 h before the addition of the indole solution. The vial was sealed and heated to the indicated temperature in a pre-heated oil bath outside of the glove box. After the indicated time, the reaction was cooled to r.t., and transferred to a round-bottom flask using $CH_2Cl_2$ to wash the tube. The reaction was concentrated *in vacuo* and purified via column chromatography to yield the corresponding borylated indole using the indicated solvent system.

### c) General Procedure C: Desilylation of Indoles

A flame-dried round bottom flask, equipped with a Teflon coated stir bar, was charged with starting indole (1.0 equiv) followed by pyridinium *p*-toluenesulfonate (PPTS) (1.2 equiv). The flask was evacuated and backfilled with $N_2$ (this process was repeated three times), then dry, degassed MeOH solvent was added under a positive $N_2$ pressure. The resulting reaction mixture was then stirred at ambient temperature for indicated time, as monitored by thin-layer chromatography (TLC). After the addition of water (30 mL) and EtOAc (30 mL), the aqueous layer was extracted with EtOAc (3 x 20 mL). The combined organic layers were washed with brine (20 mL), dried over $MgSO_4$, and concentrated *in vacuo*. The resulting residue was purified by flash chromatography on silica gel using the indicated solvent system as eluent to afford the desired product.

## 4. Synthesis and Characterization of Compounds

**<u>Compound 16</u>:**

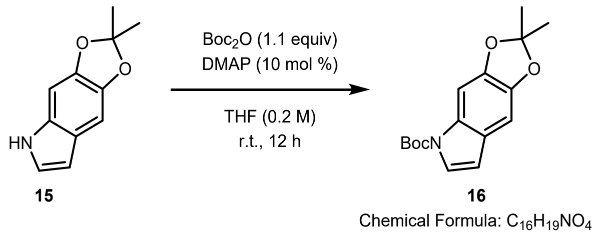


**15** **16**

Chemical Formula: $C_{16}H_{19}NO_4$
Molecular Weight: 289.33

**<u>Procedure</u>:** A 500 mL flame-dried, round-bottom flask, equipped with a Teflon-coated stir bar and rubber septum, was charged with **15** (9.5 g, 50.0 mmol, 1 equiv), DMAP (610.9 mg, 5.0 mmol, 0.1 equiv) and $Boc_2O$ (12.0 g, 55.0 mmol, 1.1 equiv) followed by THF (250 mL, 0.20 M). The resulting homogeneous reaction mixture was then stirred at ambient temperature for 12 h before concentrated *in vacuo*. The resulting crude

reaction mixture was purified by column chromatography (5% EtOAc in hexanes) to afford **16** as white solid (13.02 g, 45.0 mmol) in 90% yield.

**Characterization**:

**R*f*** = (EtOAc/hexanes, 1:9) 0.65; **IR** (neat) v = 3154, 3120, 2982, 2935, 1729, 1471, 1392, 1292, 1137, 998, 867, 835, 769, 752 $cm^{-1}$; **$^{1}$H NMR** (400 MHz, $CDCl_3$) δ 7.57 (brs, 1H), 7.44 (d, *J* = 3.7 Hz, 1H), 6.83 (s, 1H), 6.42 (dd, *J* = 3.7, 0.7 Hz, 1H), 1.69 (s, 6H), 1.67 (s, 9H); **$^{13}$C NMR** (126 MHz, $CDCl_3$) δ 149.9, 146.2, 144.5, 129.9, 124.3, 117.9, 107.5, 99.6, 96.9, 83.6, 28.3, 25.9.; **HRMS**: Calcd. For $C_{16}H_{19}O_4NNa$ $[M+Na]^+$ =312.12063 m/z, found =312.12031 m/z.

**Compound 17:**

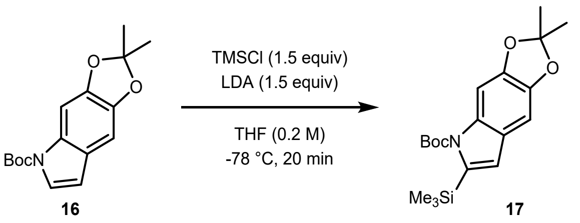


Chemical Formula: $C_{19}H_{27}NO_4Si$
Molecular Weight: 361.51

**Procedure**: A 500 mL flame-dried, round-bottom flask, equipped with a Teflon-coated stir bar and rubber septum, was charged with **16** (10.7 g, 37.0 mmol, 1 equiv). The flask was then evacuated and backfilled with $N_2$ for three times before addition of dry, degassed THF (185 mL, 0.2 M). To the resulting homogeneous reaction solution was added TMSCl (7.0 mL, 55.5 mmol, 1.5 equiv), and LDA (27.8 mL, 55.5 mmol, 1.5 equiv, 2.0 M in THF) dropwise at –78 °C in a dry ice/acetone dewer bath under a $N_2$ atmosphere. The reaction mixture was stirred at –78 °C for 20 min prior to quenching by saturated aqueous $NH_4Cl$ solution. The resulting mixture was then extracted three times with EtOAc (50 mL x 3). The combined organic fractions were washed with brine, dried over $MgSO_4$, filtered, and concentrated *in vacuo*. The resulting residue was purified using silica gel column chromatography (5% EtOAc in hexanes) to afford **17** as a white solid (12.5 g, 34.8 mmol) in 94% yield.

**Characterization**:

**R*f*** = (EtOAc/hexanes, 1:9) 0.69; **IR** (neat) v = 2979, 2903, 1726, 1472, 1382, 1326, 1203, 1159,1150, 1129, 1027, 904, 842, 768, 632 $cm^{-1}$; **$^{1}$H NMR** (500 MHz, $CDCl_3$) δ 7.41 (s, 1H), 6.80 (s, 1H), 6.68 (s, 1H), 1.69 (s, 6H), 1.69 (s, 9H), 0.31 (s, 9H). **$^{13}$C NMR** (126 MHz,

CDCl$_3$) 151.1, 146.5, 144.3, 140.5, 132.5, 124.8, 119.4, 117.9, 99.1, 97.1, 83.9, 28.4, 25.9, 0.2.; **HRMS**: Calcd. for $C_{19}H_{27}O_4NNaSi$ $[M+Na]^+$ =384.16016 m/z, found =384.15958 m/z.

**Compound 18:**

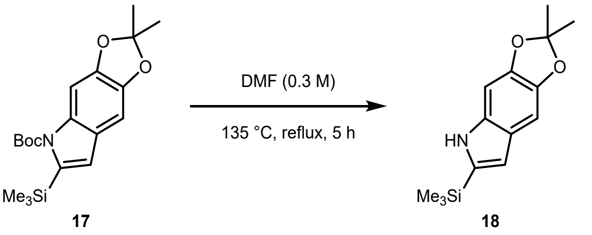


Chemical Formula: $C_{14}H_{19}NO_2Si$
Molecular Weight: 261.40

**Procedure**: A flame-dried, 250 mL round-bottom flask equipped with a Teflon-coated stir bar and rubber septum was charged with **17** (3.9 g, 10.0 mmol, 1 equiv) and DMF (36 mL, 0.3 M). The resulting homogenous solution was heated at reflux (135 °C) under $N_2$ and allowed to cool to ambient temperature after 5 h. The resulting mixture was then extracted three times with EtOAc (50 mL x 3). The combined organic fractions were washed with $H_2O$ and brine, dried over $MgSO_4$, filtered, and concentrated *in vacuo*. The resulting residue was recrystallized with toluene (13 mL) at 65 °C then cooled down to –20 °C. Filtration afforded **18** as a white crystal (2.4 g, 9.2 mmol) in 84% yield.

**Characterization**: $\mathbf{R_f}$ = (hexanes/EtOAc, 9:1) 0.53; **IR** (neat) v = 3409, 2985, 2957, 1504, 1451, 1384, 1230, 1153, 1103, 982, 948, 872, 835, 780, 752, 659, 627 cm$^{-1}$; **$^1$H NMR** (500 MHz, CDCl$_3$) δ 7.93 (brs, 1H), 6.88 (t, *J* = 0.7 Hz, 1H), 6.76 (t, *J* = 0.7 Hz, 1H), 6.58 (dd, *J* = 2.2, 1.0 Hz, 1H), 1.68 (s, 6H), 0.31 (s, 9H).; **$^{13}$C NMR** (126 MHz, CDCl$_3$) δ 145.6, 143.4, 136.3, 133.7, 122.3, 117.2, 111.5, 98.6, 91.4, 25.9, -0.8.; **HRMS**: Calcd. for $C_{14}H_{19}NNaO_2Si$ $[M+Na]^+$ = 284.1077 m/z, found = 284.1081 m/z.

**Compound 19**:

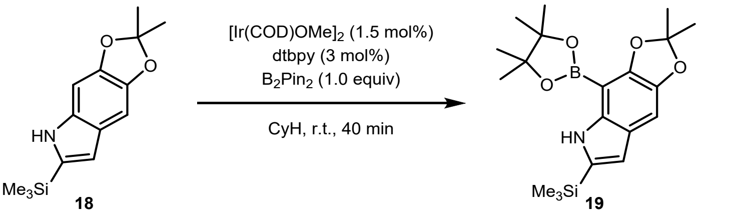


Chemical Formula: $C_{20}H_{30}BNO_4Si$
Molecular Weight: 387.36

**Procedure:** The reaction was carried out according to the General Procedure B and was performed in cyclohexanes at room temperature for 40 min.

**Amounts of Reagents**:

**18** (2.6 g, 10 mmol, 1.0 equiv)

$[Ir(OMe)(COD)]_2$ (99.43 mg, 0.15 mmol, 1.5 mol%)

dtbpy (80.52 mg, 0.30 mmol, 3.0 mol%)

$B_2pin_2$ (2.5 g, 10 mmol, 1.0 equiv)

Cyclohexanes (25 mL, 0.4M),

**Purification**: 10% EtOAc in hexanes.

**Yield of Product**:

**19**: 3.2 g, 8.3 mmol, 83%, white solid.

**Characterization**:

**R*f*** = (EtOAc/hexanes, 1:9) 0.50; **IR** (neat) v = 3441, 2982, 2954, 1613, 1452, 1385, 1289, 1194, 1137, 1104, 1061, 991, 836, 754, 667, 628 $cm^{-1}$; **$^1$H NMR** (500 MHz, $CDCl_3$) δ 9.24 (brs, 1H), 6.98 (s, 1H), 6.56 (d, *J* = 2.3 Hz, 1H), 1.70 (s, 6H), 1.40 (s, 12H), 0.32 (s, 9H).; **$^{13}$C NMR** (126 MHz, $CDCl_3$) δ 151.8, 142.9, 138.3, 135.3, 121.1, 117.3, 110.5, 102.0, 83.7, 26.0, 25.1, -0.9.; **HRMS**: Calcd. for $C_{20}H_{31}O_4NBSi$ $[M+H]^+$ =388.21099 m/z, found =388.21015 m/z.

**Compound 20**:

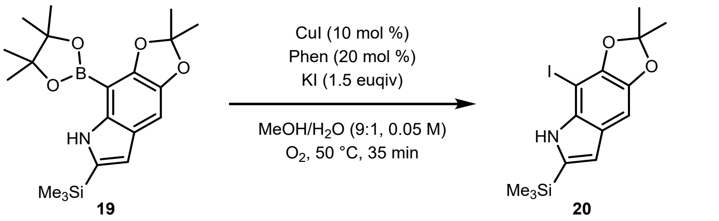


Chemical Formula: $C_{14}H_{18}INO_2Si$
Molecular Weight: 387.29

**Procedure**: A 50 mL round-bottom flask equipped with a Teflon-coated stir bar and rubber septum was charged with **19** (774.72 mg, 2 mmol, 1 equiv), CuI (38.09 mg, 0.2 mmol, 10 mol%), phenanthroline (72.08 mg, 0.4 mmol, 20 mol%), KI (498.0 mg, 3 mmol, 1.5 equiv), and $MeOH/H_2O$ (36 mL/4 mL, 0.05 M, 9:1). The rubber septum was then connected to a tank of $O_2$, pressurized to 50 kpa, and was vented 3 times for 10 s each time. Under a constant pressure of $O_2$ (50 kpa), the reaction mixture was then stirred at 50 $^oC$ for 35 min before depressurizing by opening to the atmosphere and concentrated *in vacuo* to afford a wine-red residue. The resulting mixture was then extracted three times with EtOAc (50 mL x 3). The phases were separated, and the organic factions were washed with brine, dried over $MgSO_4$, filtered, and concentrated *in vacuo*. The resulting

residue was purified using silica gel column chromatography (5% EtOAc in hexanes) to afford **20** as a white solid (549.9 mg, 1.42 mmol) in 71% yield.

**Characterization**: **R$_f$** = (EtOAc/hexanes, 1:9) 0.64; **IR** (neat) ν = 3367, 2954, 1460, 1310, 1247, 1210, 1190, 1153, 1103, 985, 945, 923, 836, 731 cm$^{-1}$; **$^1$H NMR** (500 MHz, $CDCl_3$) δ 7.90 (brs, 1H), 6.82 (d, *J* = 0.7 Hz, 1H), 6.73 (d, *J* = 2.3 Hz, 1H), 1.73 (s, 6H), 0.34 (s, 9H).; **$^{13}$C NMR** (126 MHz, cdcl$_3$) δ 147.1, 142.5, 136.6, 135.3, 121.9, 117.8, 113.1, 98.8, 53.9, 26.0, -0.9.; **HRMS**: Calcd. for $C_{14}H_{19}O_2NISi$ $[M+H]^+$ = 388.02242 m/z, found = 388.02305 m/z.

**Compound 11:**

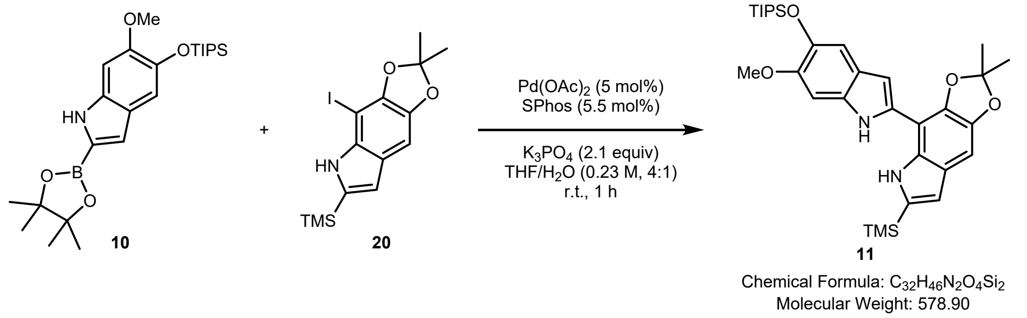


**Procedure:** The reaction was carried out according to the General Procedure A, the reaction was performed in THF/$H_2O$ at ambient temperature for 1 h.

**Amounts of Reagents**:

**10** (446.10 mg, 1.0 mmol, 1.1 equiv)

**20** (348.56 mg, 0.9 mmol, 1.0 equiv)

$Pd(OAc)_2$ (10.10 mg, 0.045 mmol, 5 mol%)

SPhos (22.58 mg, 0.049 mmol, 5.5 mol%)

$K_3PO_4$ (401.19 mg, 1.89 mmol, 2.1 equiv)

THF (4.0 mL), $H_2O$ (1.0 mL)

**Purification**: 5% to 10% EtOAc in hexanes.

**Yield of Product**:

**11:** 428.38 mg, 0.74 mmol, 82%, light yellow solid.

**Characterization**:

**R$_f$** = (EtOAc/hexane, 1:9): 0.46; **IR** (neat) ν = 3450, 2950, 2870, 1480, 1300, 1160, 835, 677, 440 cm$^{-1}$; **$^1$H NMR** (500 MHz, $CDCl_3$) δ 9.03 (brs, 1H), 8.51 (s, 1H), 7.17 (s, 1H), 6.96 (s, 1H), 6.89 (s, 1H), 6.71 (dd, *J* = 2.2, 0.8 Hz, 1H), 6.66 (d, *J* = 2.1 Hz, 1H), 3.89 (s, 3H), 1.78 (s, 6H), 1.40 – 1.27 (m, 3H), 1.15 (d, *J* = 7.4 Hz, 18H), 0.38 (s, 9H); **$^{13}$C NMR** (101

MHz, $CDCl_3$) δ 149.5, 143.2, 141.5, 141.1, 137.0, 131.1, 130.69, 130.64, 123.2, 121.8, 118.0, 112.2, 110.1, 99.7, 99.7, 98.0, 94.5, 56.0, 26.2, 18.2, 13.0, -0.8.; **HRMS**: Calcd. for $C_{32}H_{46}N_2O_4Si_2$ $[M+H]^+$ = 579.3069 m/z, found = 579.3064 m/z.

**Compound 13:**

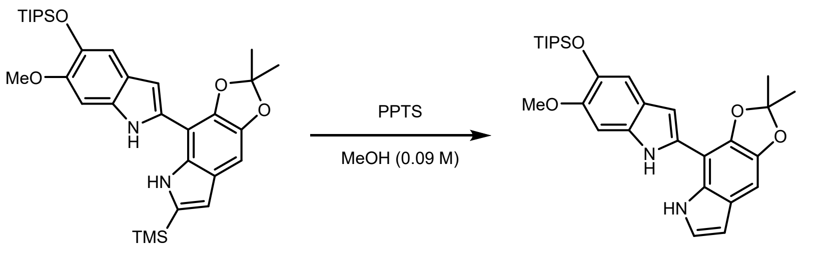


**Procedure**: The reaction was carried out according to the General Procedure C, the reaction was performed in MeOH at ambient temperature for 3 h.

**Amounts of Reagents**:

**11** (528.5 mg, 0.91 mmol, 1.0 equiv)

PPTS (228.7 mg, 0.91 mmol, 1.0 equiv)

MeOH (10.0 mL)

**Purification**: 10% EtOAc in hexanes.

**Yield of Product**:

**13:** 456.05 mg, 0.90 mmol, 97%, off-white solid.

**Characterization**:

$\mathbf{R}_f$ = (EtOAc/hexanes, 1:9) 0.34; **IR** (neat) v = 3444, 2939, 2863, 1479, 1299, 1186, 987, 852, 676, 414 $cm^{-1}$; **$^1$H NMR** (500 MHz, $CDCl_3$) δ 8.99 (s, 1H), 8.60 (s, 1H), 7.16 (t, *J* = 2.8 Hz, 1H), 7.13 (s, 1H), 6.95 (s, 1H), 6.92 (s, 1H), 6.74 (d, *J* = 2.1 Hz, 1H), 6.49 (dd, *J* = 3.2, 2.1 Hz, 1H), 3.88 (s, 3H), 1.79 (s, 6H), 1.32 (sep, *J* = 6.9 Hz, 3H), 1.14 (d, *J* = 7.5 Hz, 18H). **$^{13}$C NMR** (126 MHz, $CDCl_3$) δ 149.5, 143.2, 141.5, 140.7, 131.1, 130.4, 127.5, 123.0, 122.2, 121.8, 118.0, 110.1, 103.5, 100.0, 98.4, 94.6, 56.0, 26.2, 18.2, 18.2, 13.0.; **HRMS**: Calcd. for $C_{29}H_{38}N_2O_4Si$ $[M-H]^+$ = 505.2528 m/z, found = 505.2536 m/z.

**Compound 14:**

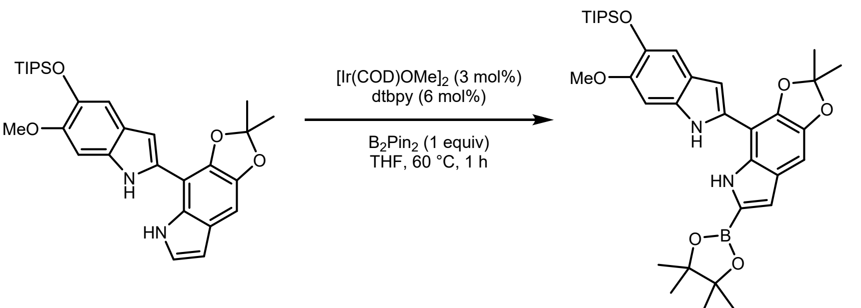


13

14

Chemical Formula: $C_{35}H_{49}BN_2O_6Si$

Molecular Weight: 632.68

**Procedure:** The reaction was carried out according to the General Procedure B, the reaction was performed in THF at 60 $^oC$ for 1 h.

**Amounts of Reagents**:

**13** (456.05 mg, 0.9 mmol, 1.0 equiv)

$[Ir(OMe)(COD)]_2$ (17.89 mg, 0.027 mmol, 3.0 mol%)

dtbpy (14.49 mg, 0.054 mmol, 6.0 mol%)

$B_2pin_2$ (228.54 mg, 0.9 mmol, 1.0 equiv)

THF (18 mL, 0.05 M)

**Purification**: 10% EtOAc in hexanes.

**Yield of Product**:

**14**: 540.9 mg, 0.85 mmol, 94%, white solid.

**Characterization**:

**$R_f$** = (EtOAc/hexanes, 8:2) 0.54; **IR** (neat) v = 3446, 2940, 2863, 1525, 1479, 1259, 1218, 1135, 852, 680 $cm^{-1}$; **$^1$H NMR** (500 MHz, $CDCl_3$) δ 8.98 (s, 1H), 8.90 (s, 1H), 7.18 (s, 1H), 7.04 (d, *J* = 2.1 Hz, 1H), 6.94 (s, 1H), 6.89 (s, 1H), 6.82 (d, *J* = 2.2Hz, 1H), 3.88 (s, 4H), 1.78 (s, 6H), 1.38 (s, 12H), 1.36 – 1.28 (m, 3H), 1.14 (d, *J* = 7.4 Hz, 18H); **$^{13}$C NMR** (126 MHz, $CDCl_3$) δ 149.4, 143.5, 142.2, 141.4, 131.2, 130.8, 130.1, 123.0, 121.8, 118.2, 115.0, 110.2, 100.57, 100.56, 99.8, 98.4, 94.4, 84.1, 55.9, 26.2, 24.9, 18.2, 13.0.; **HRMS**: Calcd. for $C_{35}H_{48}N_2O_6BSi$ $[M-H]^+$ = 631.3380 m/z, found = 631.3393 m/z.

**Compound 21:**

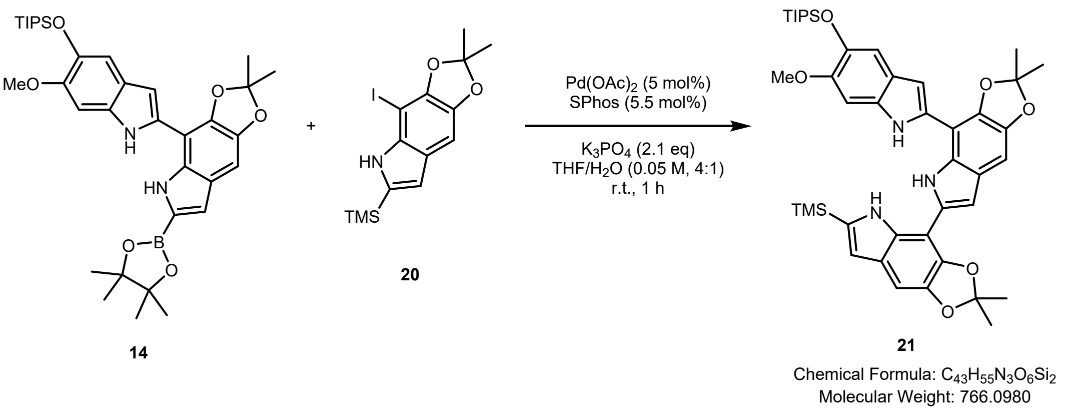

**Procedure:** The reaction was carried out according to the General Procedure A, the reaction was performed in THF/$H_2O$ at ambient temperature for 1 h.

**Amounts of Reagents**:

**14** (537.78 mg, 0.85 mmol, 1.1 equiv)

**20** (298.21 mg, 0.77 mmol, 1.0 equiv)

$Pd(OAc)_2$ (8.53 mg, 0.038 mmol, 5 mol%)

SPhos (17.24 mg, 0.042 mmol, 5.5 mol%)

$K_3PO_4$ (343.88 mg, 1.62 mmol, 2.1 equiv)

THF (15.0 mL), $H_2O$ (3.7 mL)

**Purification**: 5 % to 10 % EtOAc in hexanes.

**Yield of Product**:

**21:** 509.5 mg, 0.66 mmol, 86 %, light yellow solid.

**Characterization**:

$\mathbf{R}_f$ = (EtOAc/hexanes, 1:9) 0.33; **IR** (neat) ν = 3452, 2940, 2863, 1479, 1446, 1303, 1197, 985, 835, 754, 659, 406 $cm^{-1}$; **$^1$H NMR** (400 MHz, $CDCl_3$) δ 10.00 (s, 1H), 9.06 (s, 1H), 8.48 (s, 1H), 7.12 (s, 1H), 7.00 (s, 1H), 6.98 (s, 1H), 6.92 (s, 1H), 6.85 (d, *J* = 2.1 Hz, 1H), 6.78 (d, *J* = 2.3 Hz, 1H), 6.69 (d, *J* = 2.1 Hz, 1H), 3.90 (s, 3H), 1.85 (s, 6H), 1.83 (s, 6H), 1.36 – 1.27 (m, 3H), 1.15 (dd, *J* = 7.2 Hz, 18H), 0.40 (s, 9H); **$^{13}$C NMR** (101 MHz, $CDCl_3$) δ 149.6, 143.5, 143.3, 141.5, 141.1, 140.7, 137.2, 131.1, 130.7, 130.5, 130.4, 127.5, 123.4, 122.9, 121.9, 118.2, 118.1, 112.3, 110.0, 100.3, 99.8, 99.7, 99.6, 98.2, 97.9, 94.7, 56.1, 26.2, 26.1, 18.1, 13.0, -0.8.; **HRMS**: Calcd. for $C_{43}H_{55}N_3NaO_6Si_2$ $[M+Na]^+$ = 788.3527 m/z, found = 788.3510 m/z.

**Compound 22:**

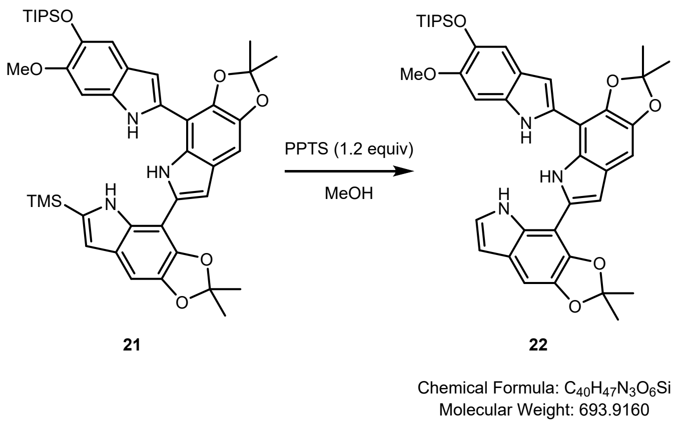


**Procedure**: The reaction was carried out according to the General Procedure C, the reaction was performed in MeOH at ambient temperature for 3 h.

**Amounts of Reagents**:

**21** (509.5 mg, 0.66 mmol, 1.0 equiv)

PPTS (199.03 mg, 0.79 mmol, 1.2 equiv)

MeOH (13.0 mL, 0.05 M)

**Purification**: 10 % EtOAc in hexanes.

**Yield of Product**:

**22:** 384.71 mg, 0.55 mmol, 84 %, off-white solid.

**Characterization**:

**R*f*** = (EtOAc/hexanes, 8:2) 0.2; **IR** (neat) v = 3450, 2939, 2863, 1446, 1301, 1197, 985, 883, 852, 661, 435 $cm^{-1}$; **$^1$H NMR** (500 MHz, $CDCl_3$) δ 10.05 (s, 1H), 9.04 (s, 1H), 8.57 (s, 1H), 7.20 (dd, *J* = 3.1, 2.4 Hz, 1H), 7.12 (s, 1H), 6.98 (s, 1H), 6.95 (s, 1H), 6.94 (s, 1H), 6.84 (dd, *J* = 3.1, 2.3f Hz, 1H), 6.80 (d, *J* = 2.3 Hz, 1H), 6.52 (dd, *J* = 3.1, 2.0 Hz, 1H), 3.89 f(s, 3H), 1.85 (s, 6H), 1.82 (s, 6H), 1.28-1.35 (m, 3H), 1.15 (d, *J* = 7.4 Hz, 18H); **$^{13}$C NMR** (126 MHz, $CDCl_3$) δ 149.6, 143.5, 143.3, 141.5, 140.73, 140.71, 131.1, 130.55, 130.53, 127.5, 127.2, 123.1, 122.8, 122.4, 122.0, 118.2, 118.1, 110.0, 103.6, 100.3, 99.9, 99.8, 99.6, 98.6, 97.9, 94.7, 56.1, 26.2, 26.1, 18.1, 13.0.; **HRMS**: Calcd. for $C_{40}H_{48}N_3O_6Si$ $[M+H]^+$ = 694.3312 m/z, found = 694.3306 m/z.

**Compound 23:**

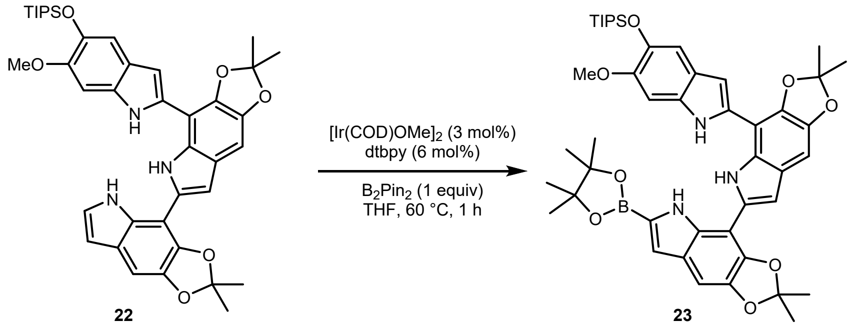


Chemical Formula: $C_{46}H_{58}BN_3O_8Si$
Molecular Weight: 819.88

**<u>Procedure</u>:** The reaction was carried out according to the General Procedure B, the reaction was performed in THF at 60 °C for 1 h.

**<u>Amounts of Reagents</u>**:

**22** (353.8 mg, 0.5 mmol, 1.0 equiv)

[Ir(OMe)(COD)]$_2$ (10.14 mg, 0.015 mmol, 3.0 mol%)

dtbpy (8.21 mg, 0.03 mmol, 6.0 mol%)

B$_2$pin$_2$ (129.5 mg, 0.5 mmol, 1.0 equiv)

THF (10 mL, 0.05 M)

**<u>Purification</u>**: 20% EtOAc in hexanes.

**<u>Yield of Product</u>**:

**23**: 350.2 mg, 0.43 mmol, 83 %, off-white solid.

**<u>Characterization</u>**:

**$R_f$** = (EtOAc/hexanes, 2:8) 0.39; **IR** (neat) ν = 3453, 2935, 2863, 1299, 1261, 1216, 1197, 1135, 852, 661, 464 cm$^{-1}$; **$^1$H NMR**(400 MHz, CDCl$_3$) δ 10.03 (s, 1H), 9.04 (s, 1H), 8.87 (s, 1H), 7.11 (s, 1H), 7.07 (d, *J* = 2.1 Hz, 1H), 7.00 (s, 1H), 6.98 (s, 1H), 6.93 (s, 1H), 6.90 (d, *J* = 2.2 Hz, 1H), 6.84 (d, 1H), 3.89 (s, 3H), 1.85 (s, 6H), 1.83 (s, 6H), 1.39 (s, 12H), 1.36 – 1.27 (m, 3H), 1.14 (d, *J* = 7.3 Hz, 18H); **$^{13}$C NMR** (101 MHz, CDCl$_3$) δ 149.5, 143.6, 143.4, 142.1, 141.5, 140.7, 131.1, 130.6, 130.4, 130.3, 127.5, 123.2, 122.9, 122.0, 118.3, 118.2, 115.0, 110.0, 100.7, 99.75, 99.69, 99.57, 98.6, 98.0, 94.7, 84.2, 56.1, 26.22, 26.16, 25.0, 18.1, 13.0.; **HRMS**: Calcd. for $C_{46}H_{57}N_3O_8BSi$ [M-H]$^+$ = 818.40135 m/z, found = 818.40014 m/z.

**<u>Compound 24:</u>**

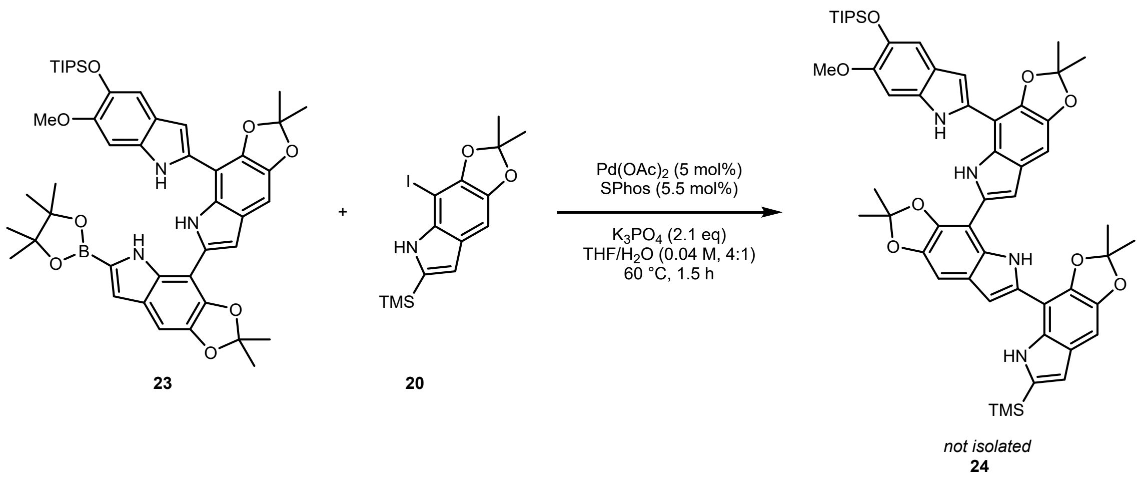


**Procedure:** The reaction was carried out according to the General Procedure A, the reaction was performed in THF/$H_2O$ at 60 °C for 1.5 h.

**Amounts of Reagents**:

**23** (352.5 mg, 0.43 mmol, 1.1 equiv)

**20** (151.0 mg, 0.39 mmol, 1.0 equiv)

$Pd(OAc)_2$ (4.38 mg, 0.019 mmol, 5 mol %)

SPhos (8.81 mg, 0.042 mmol, 5.5 mol %)

$K_3PO_4$ (173.9 mg, 0.82 mmol, 2.1 equiv)

THF (8.0 mL), $H_2O$ (2.0 mL)

**Purification**: 10 % EtOAc in hexanes.

**Yield of Product**:

**24**: 69 % (NMR yield calculated using hexamethylbenzene as an internal standard), light yellow solid.

**Characterization**:

$\mathbf{R_f}$ = (EtOAc/hexane, 1:9) 0.30; **$^1$H NMR** (500 MHz, $CDCl_3$) δ 10.12 (s, 1H), 9.95 (s, 1H), 9.06 (s, 1H), 8.50 (s, 1H), 7.14 (s, 1H), 7.03 (s, 1H), 6.99 (s, 1H), 6.93 (s, 1H), 6.93 (s, 1H), 6.87-6.88 (m, 2H), 6.81 (d, *J* = 2.1 Hz, 1H), 6.70 (d, *J* = 2.1 Hz, 1H), 3.91 (s, 3H), 1.90 (s, 6H), 1.87 (s, 6H), 1.84 (s, 6H), 1.37 – 1.29 (m, 3H), 1.16 (d, *J* = 7.4 Hz, 18H), 0.40 (s, 9H).

**Compound 25:**

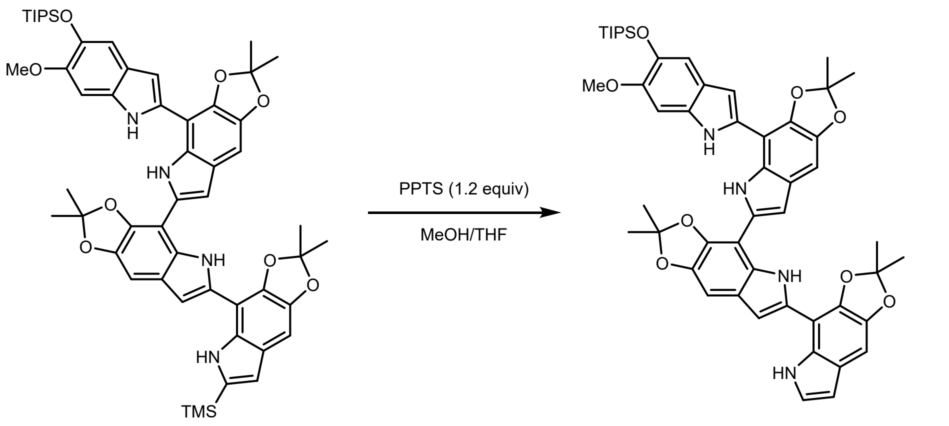

**24**

**25**

Chemical Formula: $C_{51}H_{56}N_4O_8Si$

Molecular Weight: 881.11

**Procedure**: The reaction was carried out according to the General Procedure C, the reaction was performed in MeOH and THF at ambient temperature for 6 h.

**Amounts of Reagents**:

**24** (390.0 mg, 0.40 mmol, 1.0 equiv)

PPTS (120.6 mg, 0.48 mmol, 1.2 equiv)

MeOH (10 mL), THF (2 mL)

**Purification**: 10 % EtOAc in hexanes.

**Yield of Product**:

**25:** 206.18 mg, 0.23 mmol, 60 % over two steps, off-white solid.

**Characterization**:

**R*f*** = (EtOAc/hexanes, 1:9) 0.23; **IR** (neat) ν = 34550, 2937, 2863, 1446, 1303, 1199, 1170, 983, 850, 754, 661, 435 $cm^{-1}$; **$^1$H NMR** (500 MHz, $CDCl_3$) δ 10.11 (s, 1H), 10.00 (s, 1H), 9.05 (s, 1H), 8.59 (s, 1H), 7.20 (dd, 3.2, 2.2 Hz 1H), 7.13 (s, 1H), 6.99 (m, 2H), 6.96 (s, 1H), 6.92 (s, 1H), 6.87-6.88 (m, 2H), 6.83 (d, *J* = 2.2 Hz, 1H), 6.52 (dd, *J* = 3.2, 2.1 Hz, 1H), 3.90 (s, 3H), 1.89 (s, 6H), 1.87 (s, 6H), 1.83 (s, 6H), 1.36 – 1.29 (m, 3H), 1.15 (d, *J* = 7.4 Hz, 18H); **$^{13}$C NMR** (126 MHz, $CDCl_3$) δ 149.6, 143.5, 143.3, 141.6, 140.78, 140.74, 140.72, 131.1, 130.8, 130.6, 130.5, 127.5, 127.2, 127.1, 123.15, 123.10, 123.0, 122.5, 122.0, 118.3, 118.2, 110.0, 103.6, 100.4, 100.0, 99.8, 99.7, 99.6, 98.7, 98.1, 97.7, 94.7, 56.1, 26.2, 26.1, 18.1, 13.0.; **HRMS**: Calcd. for $C_{51}H_{55}N_4O_8Si$ $[M-H]^+$ = 879.37946 m/z, found = 879.37924 m/z.

**Compound 26:**

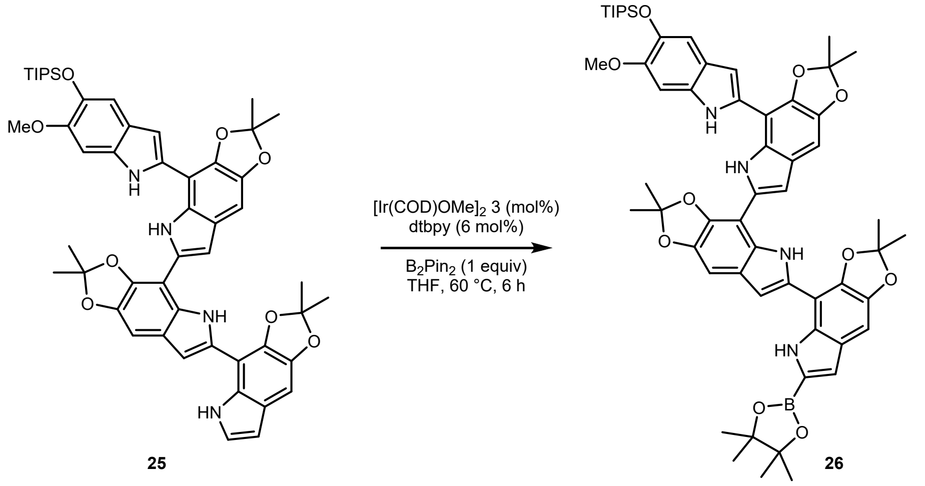


Chemical Formula: $C_{57}H_{67}BN_4O_{10}Si$
Molecular Weight: 1007.08

**Procedure:** The reaction was carried out according to the General Procedure B, the reaction was performed in THF at 60 °C for 6 h.

**Amounts of Reagents**:

**25** (72.2 mg, 0.08 mmol, 1.0 equiv)

[Ir(OMe)(COD)]$_2$ (1.59 mg, 0.0024 mmol, 3.0 mol%)

dtbpy (1.29 mg, 0.0048 mmol, 6.0 mol%)

B$_2$pin$_2$ (20.32 mg, 0.08 mmol, 1.0 equiv)

THF (1.6 mL, 0.05 M)

**Purification**: 20% EtOAc in hexanes.

**Yield of Product**:

**26**: 55.8 mg, 0.055 mmol, 70 %, white solid.

**Characterization**:

**R*f*** = (EtOAc/hexanes, 2:8) 0.43; **IR** (neat) v = 3446, 2962, 2861, 1303, 1259, 1014, 854, 796, 663, 435 cm$^{-1}$; **$^1$H NMR** (500 MHz, CDCl$_3$) δ 10.12 (brs, 1H), 9.98 (brs, 1H), 9.06 (brs, 1H), 8.91 (brs, 1H), 7.13 (s, 1H), 7.08 (d, *J* = 2.0 Hz, 1H), 7.05 (s, 1H), 6.99 (s, 1H), 6.95 – 6.93 (m, 2H), 6.92 (s, 1H), 6.89 – 6.87 (m, 2H), 3.90 (s, 3H), 1.90 (s, 6H), 1.88 (s, 6H), 1.84 (s, 6H), 1.40 (s, 12H), 1.36 – 1.29 (m, 3H), 1.16 (d, *J* = 7.4 Hz, 18H); **$^{13}$C NMR** (126 MHz, CDCl$_3$) δ 149.6, 143.6, 143.51, 143.49, 142.1, 141.6, 140.8, 140.7, 131.1, 130.7, 130.6, 130.5, 130.4, 127.5, 127.2, 123.2, 123.1, 123.0, 122.0, 118.4, 118.27, 118.25, 115.0, 110.0, 100.9, 100.0, 99.9, 99.71, 99.67, 99.5, 98.7, 98.2, 97.6, 94.7, 84.2, 56.1, 26.3, 26.2, 26.1, 25.0, 18.1, 13.0, 1.2; **HRMS**: Calcd. for $C_{57}H_{68}N_4O_{10}BSi$ $[M+H]^+$ = 1007.47923 m/z, found = 1007.47881 m/z.

## Compound 27:

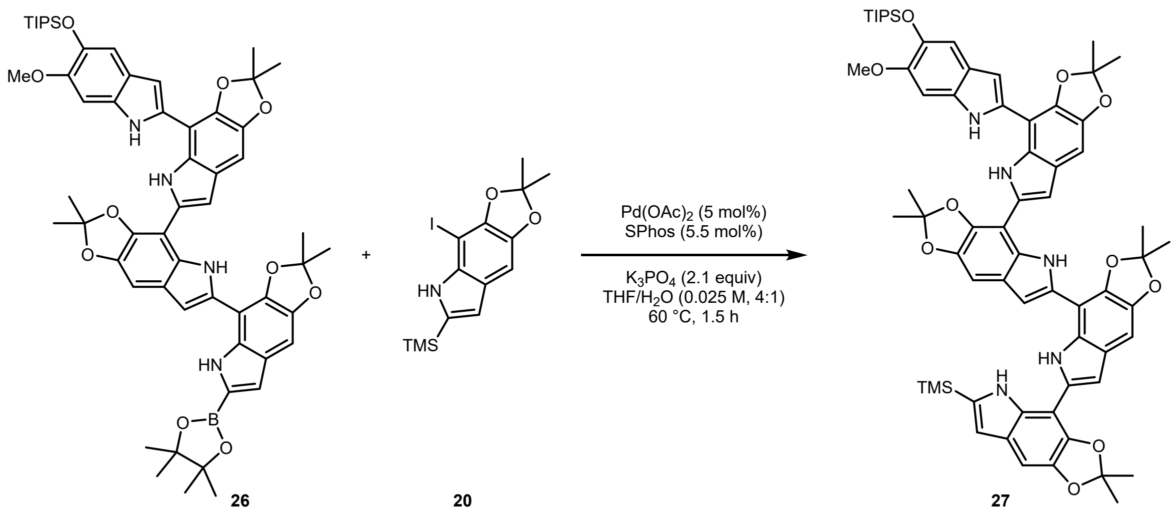


Chemical Formula: $C_{65}H_{73}N_5O_{10}Si_2$
Molecular Weight: 1140.49

**Procedure:** The reaction was carried out according to the General Procedure A, the reaction was performed in THF/$H_2O$ at 60 °C for 1.5 h.

**Amounts of Reagents**:

**26** (55.8 mg, 0.055 mmol, 1.1 equiv)

**20** (19.4 mg, 0.05 mmol, 1.0 equiv)

$Pd(OAc)_2$ (0.56 mg, 0.0025 mmol, 5 mol %)

SPhos (1.13 mg, 0.00275 mmol, 5.5 mol %)

$K_3PO_4$ (22.3 mg, 0.11 mmol, 2.1 equiv)

THF (2.0 mL), $H_2O$ (0.5 mL)

**Purification**: 10 % to 20 % EtOAc in hexanes.

**Yield of Product**:

**27:** 39.2 mg, 0.034 mmol, 68 %, light yellow solid.

**Characterization**:

**$R_f$** = (EtOAc/hexanes, 2:8) 0.47; **$^1$H NMR** (500 MHz, $CDCl_3$) δ 10.13 (d, *J* = 2.2 Hz, 1H), 10.07 (d, *J* = 2.3 Hz, 1H), 9.97 (d, *J* = 2.2 Hz, 1H), 9.06 (d, *J* = 2.2 Hz, 1H), 8.50 (brs, 1H), 7.14 (s, 1H), 7.04 (s, 1H), 7.00 (s, 1H), 6.96 (s, 1H), 6.94 (m, 2H), 6.92 (d, *J* = 2.2 Hz, 2H), 6.89 (d, *J* = 2.1 Hz, 1H), 6.82 (d, *J* = 2.2 Hz, 1H), 6.70 (d, *J* = 2.1 Hz, 1H), 3.91 (s, 3H), 1.91 (s, 6H), 1.90 (s, 6H), 1.87 (s, 6H), 1.84 (s, 6H), 1.37 – 1.29 (m, 3H), 1.16 (d, *J* = 7.5 Hz, 18H), 0.40 (s, 9H).; **$^{13}$C NMR** (126 MHz, $CDCl_3$) δ 149.6, 143.6, 143.51, 143.49, 142.1,

141.6, 140.8, 140.7, 131.1, 130.7, 130.6, 130.5, 130.4, 127.5, 127.2, 123.2, 123.1, 123.0, 122.0, 118.4, 118.27, 118.25, 115.0, 110.0, 100.9, 100.0, 99.9, 99.71, 99.67, 99.5, 98.7, 98.2, 97.6, 94.7, 84.2, 56.1, 26.3, 26.2, 26.1, 25.0, 18.1, 13.0, 1.2.; **HRMS**: Calcd. for $C_{65}H_{73}N_5NaO_{10}Si_2$ $[M+Na]^+$ = 1162.4788 m/z, found = 1162.4802 m/z.

**Compound 28:**

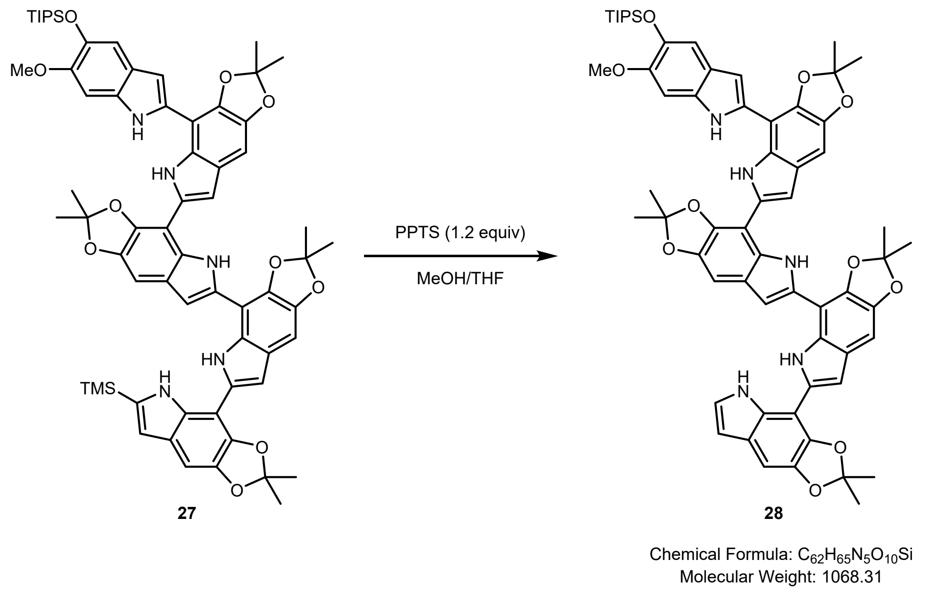


**Procedure**: The reaction was carried out according to the General Procedure C, the reaction was performed in MeOH and THF at ambient temperature for 6 h.

**Amounts of Reagents**:

**27** (39.2 mg, 0.034 mmol, 1.0 equiv)

PPTS (10.3 mg, 0.041mmol, 1.2 equiv)

MeOH (2.0 mL), THF (0.2 mL)

**Purification**: 20 % EtOAc in hexanes.

**Yield of Product**:

**28:** 206.18 mg, 0.03 mmol, 89 %, off-white solid.

**Characterization**:

**R*f*** = (EtOAc/hexanes, 2:8) 0.14; **IR** (neat) ν = 3444, 2935, 2863, 1446, 1303, 1199, 1170, 983, 852, 755, 663 $cm^{-1}$; **$^1$H NMR** (500 MHz, $CDCl_3$) δ 10.12 (d, *J* = 2.2 Hz, 1H), 10.08 (d, *J* = 2.2 Hz, z1H), 10.01 (d, *J* = 2.0 Hz, 1H), 9.06 (d, *J* = 2.1 Hz, 1H), 8.59 (brs, 1H), 7.21 (dd, *J* = 3.1, 2.4 Hz, 1H), 7.14 (s, 1H), 6.99 (s, 2H), 6.96 (s, 2H), 6.94 (s, 1H), 6.91 (m, 2H), 6.88 (d, *J* = 2.1 Hz, 1H), 6.84 (d, *J* = 2.2 Hz, 1H), 6.53 (dd, *J* = 3.1, 2.1 Hz, 1H), 3.90 (s, 3H), 1.91

(s, 6H), 1.90 (s, 6H), 1.88 (s, 6H), 1.84 (s, 6H), 1.37 – 1.28 (m, 3H), 1.16 (d, *J* = 7.4 Hz, 18H).; **$^{13}$C NMR** (201 MHz, $CDCl_3$) δ 149.6, 143.59, 143.55, 143.51, 143.3, 141.6, 140.81, 140.77, 140.75, 131.1, 130.9, 130.8, 130.6, 130.5, 127.5, 127.2, 127.15, 127.12, 123.22, 123.15, 123.1, 123.0, 122.5, 122.0, 118.38, 118.35, 118.31, 118.2, 110.0, 103.6, 100.4, 100.14, 100.08, 99.88, 99.86, 99.73, 99.69, 99.55, 98.7, 98.1, 97.8, 97.7, 94.7, 56.1, 26.27, 26.26, 26.24, 26.17, 18.1, 13.0.; **HRMS**: Calcd. for $C_{62}H_{65}N_5NaO_{10}Si$ $[M+Na]^+$ = 1090.4393 m/z, found = 1090.4346 m/z.

## 5. Spectroscopy

**Materials**

Acetonitrile (≥ 99%) and cyclohexane (99.8%) were purchased from Thermo Scientific (USA). Sulfuric acid (95.0-98.0%) and quinine hemisulfate salt monohydrate (QS) were purchased from Sigma-Aldrich (USA). 5,6-Dihydroxyindole (DHI) was purchased from Chem-Impex International (USA). Polyethersulfone (PES) syringe filters were manufactured by Sartorius (Germany) and purchased from Fisher Scientific.

**Sample preparation**

All samples were prepared by dissolving solutes in air-equilibrated solvents at room temperature. The commercial DHI sample was dissolved in ultrapure water and then filtered through a 0.22 µm PES filter. Absorption spectra were recorded with a Cary 5000 (UV/Vis/NIR) spectrometer (Agilent, USA) in the dual beam mode using solutions held in a 1 cm path length fused silica cuvette. Emission and excitation spectra were recorded from solutions held in a 1 cm path length fused silica cuvette using a QuantaMaster 8000 Fluorimeter (Horiba, USA). Luminescence spectra were collected in the standard 90° geometry and emitted photons were detected with a photomultiplier tube. Spectra were acquired using a step size of 1 nm and a detector integration time of 0.2 s. The emission and excitation slits were 1 nm. The excitation wavelength was 285 nm for the monomers **9** and **15** and 295 nm for the oligomers. The excitation wavelength for 5,6-DHI was 275 nm. Fluorescence quantum yields were estimated as described elsewhere,[1] using quinine sulfate (QS) in 0.5 M sulfuric acid as a standard ($\Phi_f$ = 0.546).

**Determination of Effective Conjugation Length**

The solid black curve in Figure 6a shows a fit to the wavelength of the 0-0 emission peak of compounds 13 ($n = 1$), 22 ($n = 2$), 25 ($n = 3$), and 28 ($n = 4$) using an equation due to Meier et al.,[2]

$$\lambda_n = \lambda_\infty - (\lambda_\infty - \lambda_1)\exp\left[-\frac{(n-1)}{\tau}\right]$$

$$= \lambda_\infty - \Delta\lambda\exp\left[-\frac{(n-1)}{\tau}\right]$$

where $n$ is the number of acetonide-protected units, $\lambda_n$ is the 0-0 emission wavelength of the compound with *n* units, $\Delta\lambda$ is the total emission shift between the monomer with wavelength $\lambda_1$ and the compound with the longest wavelength emission, $\lambda_\infty$, and $\tau$ describes the rate of variation with $n$. Following the criterion suggested in Meier et al.,[2] we define the effective conjugation length, $n_{ECL}$, by the relation $\lambda_\infty - \lambda_{n_{ECL}} = 1\,n$m. This is an appropriate measure of conjugation length because all peaks with $n > n_{ECL}$ will not differ from $\lambda_\infty$ by more than the approximate resolution of the spectrometer (1 nm).

The best -fit parameters for the curve in Figure 6a are $\tau = 0.60 \pm 0.07$, $\Delta\lambda = 31.7 \pm 1.1\,nm$, and $\lambda_\infty = 371.8 \pm 0.8$ nm. All uncertainties are one standard deviation. The effective conjugation length was calculated with the above criterion using the equation,

$$n_{ECL} = 1 + \tau\ln\left(\frac{\Delta\lambda}{1\,nm}\right)$$

yielding a value 3.1 ± 0.2.

**Table S1**. Emission peak positions of the monomers and oligomers.

| | **Peak positions in $I_{em}(\tilde{\nu})$ (cm$^{-1}$)[a]** | | | | **Peak positions in $I_{em}(\lambda)$ (nm)** | | | |
|---|---|---|---|---|---|---|---|---|
| | | | | acetonitrile | | | | |
| **9** | 30500 | | | | 326 | | | |
| **15** | 29600 | | | | 338 | | | |
| **13** | 27400 | 26200 | 24800 | | 367 | 381 | 405 | |
| $\Delta\tilde{\nu}_{em}$ | 1200 | 1400 | | | | | | |
| **22** | 25600 | 24400 | 23000 | | 390 | 408 | 436 | |
| $\Delta\tilde{\nu}_{em}$ | 1200 | 1400 | | | | | | |
| **25** | 25400 | 24200 | 22800 | 21300 | 393 | 413 | 439 | 467 |
| $\Delta\tilde{\nu}_{em}$ | 1200 | 1400 | 1500 | | | | | |
| **28** | 25400 | 24000 | 22600 | | 394 | 414 | 442 | |
| $\Delta\tilde{\nu}_{em}$ | 1400 | 1400 | | | | | | |
| | | | | cyclohexane | | | | |
| **9** | 32200 | 31500 | 30700 | | 312 | 317 | 325 | |
| $\Delta\tilde{\nu}_{em}$ | 700 | 800 | | | | | | |
| **15** | 31300 | 30600 | 29900 | 29200 | 320 | 327 | 335 | 343 |
| $\Delta\tilde{\nu}_{em}$ | 700 | 700 | 700 | | | | | |
| **13** | 27800 | 26300 | 24900 | 23500 | 360 | 380 | 402 | 428 |
| $\Delta\tilde{\nu}_{em}$ | 1500 | 1400 | 1400 | | | | | |
| **22** | 26000 | 24600 | 23200 | 21600 | 385 | 407 | 431 | 457 |
| $\Delta\tilde{\nu}_{em}$ | 1400 | 1400 | 1600 | | | | | |
| **25** | 25600 | 24300 | 22900 | 21400 | 390 | 413 | 438 | 467 |
| $\Delta\tilde{\nu}_{em}$ | 1300 | 1400 | 1500 | | | | | |
| **28** | 25500 | 24100 | 22700 | 21100 | 392 | 414 | 440 | 471 |

| $\Delta\tilde{\nu}_{em}$ | 1400 | 1400 | 1600 | |
|---|---|---|---|---|

[a]Emission spectra recorded vs. wavelength, $I_{em}(\lambda)$, were multiplied by $\lambda^2$ to obtain $I_{em}(\tilde{\nu} = 1/\lambda)$.

**Table S2**. Long wavelength absorption peak in the indicated solvents.

| Compound | $\lambda_{0-0}$ (nm) |
|---|---|
| acetonitrile | |
| **9** | 307 |
| **15** | 310 |
| **13** | 353 |
| **22** | 353 |
| **25** | 382 |
| **28** | 382 |
| cyclohexane | |
| **9** | 307 |
| **15** | 316 |
| **13** | 354 |
| **22** | 377 |
| **25** | 383 |
| **28** | 386 |

**Table S3**. Emission peak intensity ratio of the first two emission subbands of the oligomers.

| | Solvent | $\frac{I_{0-1}(\tilde{\nu})}{I_{0-0}(\tilde{\nu})}$ |
|---|---|---|
| **13** | $CH_3CN$ | 1.3 |
| | $C_6H_{12}$ | 0.95 |
| **22** | $CH_3CN$ | 1.2 |
| | $C_6H_{12}$ | 0.92 |
| **25** | $CH_3CN$ | 1.1 |
| | $C_6H_{12}$ | 0.75 |
| **28** | $CH_3CN$ | 1.1 |
| | $C_6H_{12}$ | 0.64 |

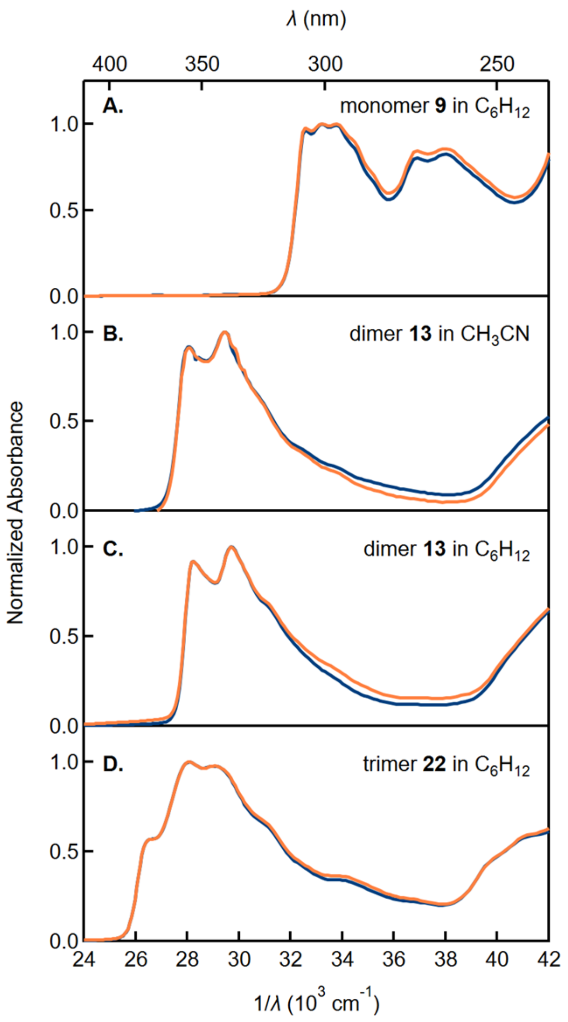


**Figure S1**. Absorption spectra of monomer **9** (A), dimer **13** (B, C), and trimer **22** (D) measured in cyclohexane ($C_6H_{12}$) or acetonitrile ($CH_3CN$), as indicated. Spectra were recorded from freshly prepared solutions (blue curves) and from the same solutions 20 months later (orange curves).

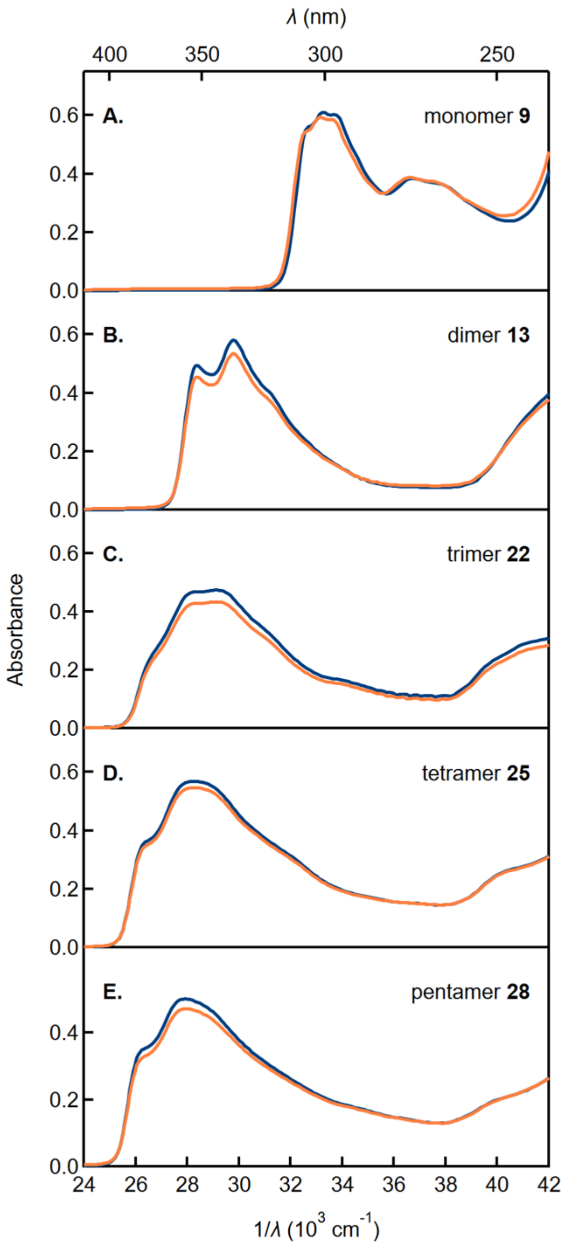


**Figure S2**. Absorption spectra of monomer **9** (A), dimer **13** (B), trimer **22** (C), tetramer **25** (D), and pentamer **28** (E) in acetonitrile before (blue curves) and after (orange curves) irradiation for 30 minutes by 100 fs laser pulses (1 kHz pulse repetition rate; incident energy density 170 μJ $cm^{-2}$; center wavelength of 300 nm for the monomer and dimer, 315 nm for the trimer, tetramer, and pentamer).

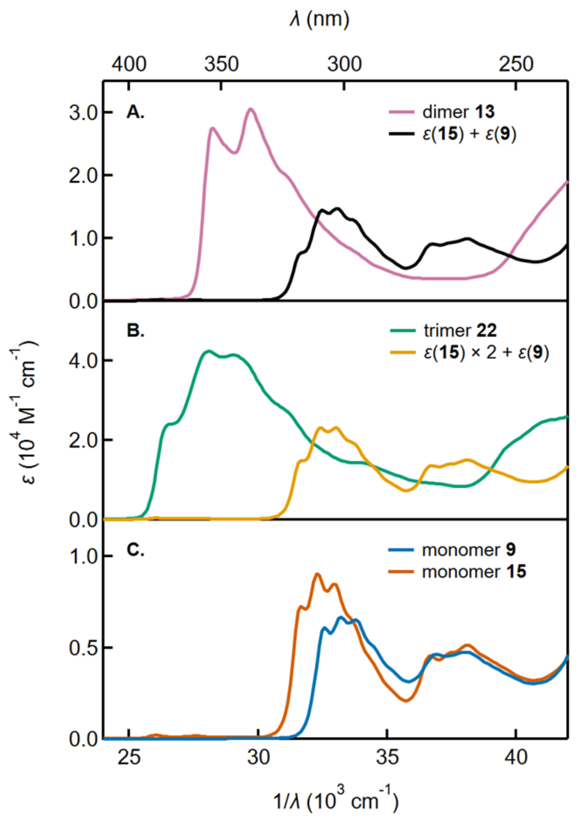


**Figure S3**. (A) Extinction spectrum of dimer **13** (pink curve) compared with the sum of the extinction spectrum of monomer **15** plus the extinction spectrum of monomer **9** (black curve). (B) Extinction spectrum of trimer **22** (green curve) compared with the sum of twice the extinction spectrum of monomer **15** plus the extinction spectrum of monomer **9** (yellow curve). (C) Extinction spectra of monomers **9** (blue curve) and **15** (red curve). (A-C) All spectra were recorded in cyclohexane solution and extinction values are believed to be accurate to within 10%.

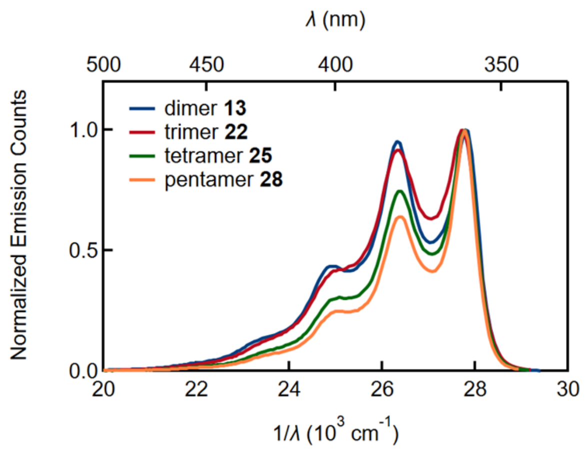

**Figure S4**. Normalized emission spectra of dimer **13** (blue curve), trimer **22** (red curve), tetramer **25** (green curve) and pentamer **28** (orange curve) in cyclohexane. The latter three curves were shifted along the frequency axis to align the long wavelength emission band with that of the dimer.

## 6. Computational Details

Calculations were carried out with Gaussian 16 [Gaussian 16, Revision A.03. Frisch, M. *et al*. Gaussian, Inc., Wallingford CT, 2019.], using density functional theory with the PBE0 hybrid functional and the 6-311g(d,p) basis set. CAM-B3LYP calculations were done for comparison. Bulk solvation is included with the universal solvation model SMD.[3] Excited states were obtained with time-dependent DFT (TD-DFT). In the computational models, the TIPSO group is replaced by a MeO group, and we use the conformations with an intramolecular hydrogen between the fragments. The vibrational frequencies provided for dimer **13-cal** in the main text were obtained in acetonitrile, using a scaling factor of 0.96.[4]

The choice of the functional has been based on the optimal tuning criterion applied to the tetramer at its ground-state geometry. This criterion is based on the ionization energy (IE) theorem of exact DFT, that states that IE = -$\varepsilon_{HOMO}$ for the exact Kohn-Sham functional.[5] Following this criterion, the ionization energy calculated with the $\Delta$SCF approach (difference between energy of the ionized and neutral system) is compared with -$\varepsilon_{HOMO}$ for the neutral system for the PBE0, CAM-B3LYP and LC-$\omega$HPBE functionals implemented in Gaussian 16. In the latter case, different values of the range-separation parameter are tested (see Table S4). For PBE0 and CAM-B3LYP, the difference IE - $\varepsilon_{HOMO}$ is -0.29 eV and -0.99 eV, respectively. For LC-$\omega$HPBE, this difference can be reduced (in absolute value) to 0.12 eV with a value of $\omega$ = 0.005 a.u. Considering that PBE0 provides a good approximation to the IE theorem and that the optimal value of $\omega$ for LC-$\omega$HPBE is close to zero, we chose PBE0 as the functional for this study. CAM-B3LYP data are provided below for comparison.

**Table S4.** Comparison of the ionization energy calculated with the ΔSCF approach and $\varepsilon_{HOMO}$ for different functionals. Basis set: 6-311G(d,p). Geometry: ground-state minimum of the tetramer **25-cal**.

| Functional | IE (ΔSCF) (hartree) | $-\varepsilon_{HOMO}$ (hartree) | IE-$\varepsilon_{HOMO}$ (eV) |
|---|---|---|---|
| PBE0 | 0.1804 | -0.1912 | -0.29 |
| CAM-B3LYP | 0.1918 | -0.2282 | -0.99 |
| LC-wHPBE ($\omega$ = 0.4) | 0.2054 | -0.2721 | -1.81 |
| LC-wHPBE ($\omega$ = 0.2) | 0.1868 | -0.2384 | -1.40 |
| LC-wHPBE ($\omega$ = 0.1) | 0.1734 | -0.2068 | -0.91 |
| LC-wHPBE ($\omega$ = 0.05) | 0.1671 | -0.1844 | -0.47 |
| LC-wHPBE ($\omega$ = 0.005) | 0.1644 | -0.1601 | 0.12 |
| LC-wHPBE ($\omega$ = 0.001) | 0.1644 | -0.1579 | 0.18 |

The $S_0$ and $S_1$ energy profiles along the interfragment rotational coordinate in the vicinity of the equilibrium geometry have been calculated for dimer **13-cal**. Results are displayed in Figure S5.

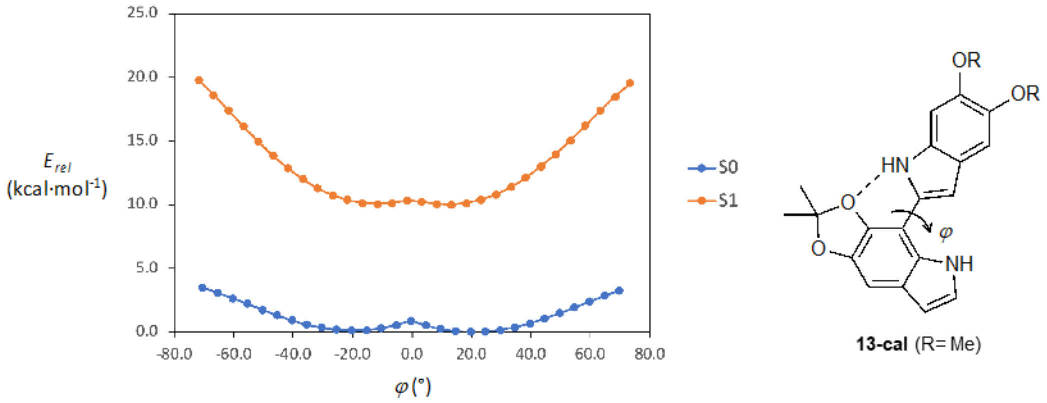


**Figure S5**. PBE0/6-311g(d,p) potential energy profiles (cyclohexane bulk solvation) for $S_0$ (blue) and $S_1$ (orange) along the rotational dihedral angle $\varphi$ of dimer **13-cal**, centered on the hydrogen bonded conformers. Energy relative to the minimum of each curve, with the $S_1$ energy profile shifted by 10 kcal·mol$^{-1}$ for convenience.

Computed absorption and emission data are provided in Table S5. Calculated absorption band maxima correspond to the lowest-energy band from the simulated spectra

presented in Figure S6, which are based on the vertical excitation spectra presented in Table S6. Calculated 0-0 band wavelengths correspond to the energy difference between the ground-state and excited-state energy at the $S_0$ and $S_1$ minima, respectively, and the vertical emission is the vertical $S_1$ energy at the $S_1$ minimum. For the oligomers that have more than one $S_1$ minimum, the 0-0 band and emission wavelength for the lowest energy exciton configuration are provided.

**Table S5**. Calculated wavelength corresponding to absorption and emission vertical energy and 0-0 energy for monomer **15** through pentamer **28-cal** in cyclohexane and acetonitrile, using the PBE0 and CAM-B3LYP functionals. For the oligomers, the emission of the lowest energy excitonic configuration is reported.

| | Cyclohexane | | | | | | Acetonitrile | | | | | |
|---|---|---|---|---|---|---|---|---|---|---|---|---|
| | PBE0 | | | CAM-B3LYP | | | PBE0 | | | CAM-B3LYP | | |
| Compound | Abs | 0-0 | Emi | Abs | 0-0 | Emi | Abs | 0-0 | Emi | Abs | 0-0 | Emi |
| **15** | 274 | 287 | 302 | 266 | 279 | 292 | 274 | 296 | 314 | 265 | 286 | 301 |
| **13-cal** | 339 | 361 | 382 | 309 | 337 | 366 | 333 | 384 | 414 | 308 | 357 | 398 |
| **22-cal** | 356 | 386 | 416 | 318 | 344 | 367 | 355 | 399 | 431 | 316 | 359 | 395 |
| **25-cal** | 361 | 393 | 425 | 319 | 346 | 373 | 358 | 402 | 435 | 317 | 362 | 398 |
| **28-cal** | 363 | 395 | 427 | 321 | 347 | 373 | 360 | 403 | 435 | 319 | 361 | 398 |

Absorption and emission energies calculated with PBE0 reproduce the experimental trends from dimer to pentamer with generally good accuracy (Table S5). The differences between calculated 0-0 energies of the oligomers and the highest-energy subband in the emission spectra (Table S1) in cyclohexane are a few nm, while the calculated emission wavelength is slightly overestimated by 8-16 nm in acetonitrile. For CAM-B3LYP, the differences with respect to the experimental data lie between 10 and 47 nm. This further supports the choice of the PBE0 functional.

**Table S6.** Calculated vertical absorption spectra for compounds **15**, **13-cal**, **22-cal**, **25-cal** and **28-cal** in cyclohexane and acetonitrile, at the PBE0 and CAM-B3LYP levels of theory.

| | | PBE0 | | | | CAM-B3LYP | | | |
|---|---|---|---|---|---|---|---|---|---|
| | | Cyclohexane | | Acetonitrile | | Cyclohexane | | Acetonitrile | |
| | | $E_{ex}$ (eV) | *f* | $E_{ex}$ (eV) | *f* | $E_{ex}$ (eV) | *f* | $E_{ex}$ (eV) | *f* |
| **15** | $S_1$ | 4.51 | 0.300 | 4.51 | 0.283 | 4.66 | 0.301 | 4.67 | 0.284 |
| | $S_2$ | 4.89 | 0.092 | 4.87 | 0.078 | 5.13 | 0.123 | 5.12 | 0.106 |
| | $S_3$ | 5.80 | 0.150 | 5.80 | 0.145 | 6.08 | 0.194 | 6.08 | 0.185 |
| **13-cal** | $S_1$ | 3.66 | 0.945 | 3.66 | 0.925 | 4.00 | 1.014 | 4.01 | 0.987 |
| | $S_2$ | 3.99 | 0.147 | 3.96 | 0.149 | 4.32 | 0.192 | 4.30 | 0.191 |
| | $S_3$ | 4.24 | 0.044 | 4.23 | 0.039 | 4.71 | 0.057 | 4.71 | 0.050 |
| | $S_4$ | 4.52 | 0.001 | 4.50 | 0.002 | 4.99 | 0.016 | 4.98 | 0.016 |
| | $S_5$ | 4.77 | 0.031 | 4.78 | 0.025 | 5.39 | 0.012 | 5.40 | 0.017 |
| | $S_6$ | 5.27 | 0.003 | 5.26 | 0.000 | 5.82 | 0.113 | 5.83 | 0.096 |
| **22-cal** | $S_1$ | 3.43 | 0.601 | 3.45 | 0.577 | 3.85 | 0.832 | 3.87 | 0.792 |
| | $S_2$ | 3.52 | 0.640 | 3.51 | 0.610 | 3.94 | 0.852 | 3.96 | 0.826 |
| | $S_3$ | 3.83 | 0.180 | 3.82 | 0.179 | 4.27 | 0.249 | 4.26 | 0.247 |
| | $S_4$ | 3.99 | 0.257 | 3.98 | 0.265 | 4.49 | 0.113 | 4.48 | 0.117 |
| | $S_5$ | 4.08 | 0.017 | 4.07 | 0.012 | 4.67 | 0.020 | 4.68 | 0.022 |
| | $S_6$ | 4.24 | 0.109 | 4.24 | 0.050 | 4.87 | 0.091 | 4.87 | 0.082 |
| | $S_7$ | 4.27 | 0.194 | 4.27 | 0.251 | 5.07 | 0.037 | 5.07 | 0.044 |
| | $S_8$ | 4.48 | 0.010 | 4.47 | 0.012 | 5.22 | 0.062 | 5.22 | 0.056 |
| | $S_9$ | 4.53 | 0.059 | 4.51 | 0.054 | 5.40 | 0.068 | 5.42 | 0.078 |
| **25-cal** | $S_1$ | 3.38 | 0.202 | 3.40 | 0.052 | 3.83 | 1.086 | 3.85 | 0.960 |
| | $S_2$ | 3.41 | 0.871 | 3.43 | 0.907 | 3.86 | 0.276 | 3.87 | 0.374 |
| | $S_3$ | 3.45 | 0.584 | 3.45 | 0.619 | 3.94 | 1.163 | 3.97 | 1.112 |
| | $S_4$ | 3.77 | 0.048 | 3.76 | 0.044 | 4.26 | 0.202 | 4.27 | 0.198 |
| | $S_5$ | 3.79 | 0.217 | 3.79 | 0.195 | 4.39 | 0.092 | 4.39 | 0.091 |
| | $S_6$ | 3.87 | 0.601 | 3.88 | 0.633 | 4.52 | 0.158 | 4.52 | 0.161 |
| | $S_7$ | 4.00 | 0.023 | 4.02 | 0.022 | 4.66 | 0.029 | 4.68 | 0.027 |
| | $S_8$ | 4.03 | 0.034 | 4.03 | 0.032 | 4.81 | 0.029 | 4.82 | 0.030 |
| | $S_9$ | 4.17 | 0.002 | 4.16 | 0.002 | 4.97 | 0.002 | 4.98 | 0.002 |
| | $S_{10}$ | 4.25 | 0.088 | 4.24 | 0.100 | 5.07 | 0.052 | 5.08 | 0.048 |
| | $S_{11}$ | 4.28 | 0.017 | 4.28 | 0.013 | 5.15 | 0.167 | 5.14 | 0.172 |
| | $S_{12}$ | 4.32 | 0.130 | 4.31 | 0.130 | 5.29 | 0.053 | 5.30 | 0.049 |
| **28-cal** | $S_1$ | 3.36 | 0.045 | 3.37 | 0.049 | 3.80 | 1.379 | 3.83 | 1.274 |
| | $S_2$ | 3.38 | 0.915 | 3.40 | 0.149 | 3.83 | 0.081 | 3.84 | 0.058 |
| | $S_3$ | 3.39 | 0.518 | 3.41 | 1.119 | 3.86 | 0.776 | 3.87 | 0.870 |
| | $S_4$ | 3.42 | 0.671 | 3.42 | 0.740 | 3.94 | 1.128 | 3.96 | 1.061 |
| | $S_5$ | 3.65 | 0.035 | 3.64 | 0.040 | 4.26 | 0.193 | 4.26 | 0.197 |
| | $S_6$ | 3.73 | 0.415 | 3.73 | 0.409 | 4.34 | 0.136 | 4.34 | 0.131 |
| | $S_7$ | 3.78 | 0.344 | 3.77 | 0.263 | 4.42 | 0.035 | 4.42 | 0.036 |
| | $S_8$ | 3.79 | 0.104 | 3.79 | 0.181 | 4.54 | 0.187 | 4.53 | 0.193 |
| | $S_9$ | 3.90 | 0.013 | 3.91 | 0.016 | 4.66 | 0.017 | 4.67 | 0.018 |
| | $S_{10}$ | 4.01 | 0.059 | 4.01 | 0.071 | 4.78 | 0.051 | 4.79 | 0.053 |
| | $S_{11}$ | 4.06 | 0.012 | 4.04 | 0.037 | 4.92 | 0.004 | 4.92 | 0.004 |
| | $S_{12}$ | 4.07 | 0.244 | 4.07 | 0.211 | 4.99 | 0.036 | 5.00 | 0.032 |
| | $S_{13}$ | 4.13 | 0.025 | 4.13 | 0.027 | 5.05 | 0.108 | 5.05 | 0.135 |
| | $S_{14}$ | 4.15 | 0.037 | 4.14 | 0.038 | 5.12 | 0.124 | 5.12 | 0.109 |
| | $S_{15}$ | 4.21 | 0.006 | 4.20 | 0.006 | 5.15 | 0.022 | 5.15 | 0.017 |

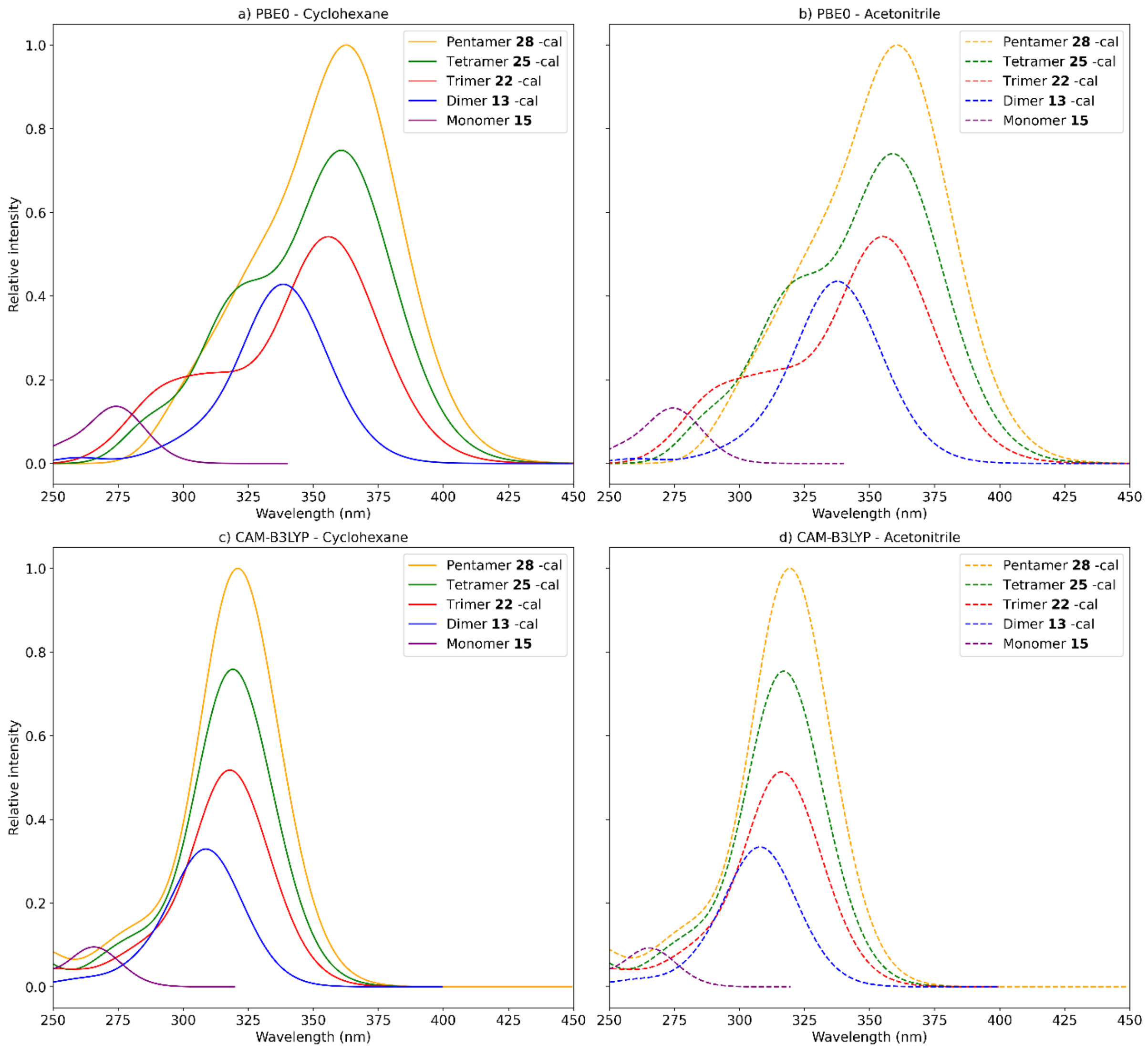


**Figure S6.** Simulated absorption spectra of the lowest energy band for compounds **15**, **13-cal**, **22-cal**, **25-cal** and **28-cal** in cyclohexane and acetonitrile, at the PBE0 and CAM-B3LYP levels of theory. Spectra were calculated on an energy scale with a half width at half maximum of 0.2 eV and converted to a wavelength scale for the plots.

The geometric parameters describing co-planarity of ground- and excited-state minima (interfragment $C_2$-$C_{7'}$ distance, rotational dihedral angle around the $C_2$-$C_{7'}$ bond) are provided in Table S7 and S8.

**Table S7**. Relative energy,[a] calculated interfragment $C_2$-$C_{7'}$ distance,[b] rotational dihedral angle around the $C_2$-$C_{7'}$ bond,[b,c] and dipole moment $\mu$ for the ground and excited state minima in cyclohexane.

| | | $E_{rel}$ (eV) | $r_{AB}$ (Å) | $\varphi_{AB}$ (°) | $r_{BC}$ (Å) | $\varphi_{BC}$ (°) | $r_{CD}$ (Å) | $\varphi_{CD}$ (°) | $r_{DE}$ (Å) | $\varphi_{DE}$ (°) | $\mu$ (D) |
|---|---|---|---|---|---|---|---|---|---|---|---|
| **13-cal** | $S_0$-Min | 0.00 | 1.452 | 20.4 | | | | | | | 1.9 |
| | $S_1$-Min | 3.44 | 1.406 | 11.7 | | | | | | | 5.2 |
| **22-cal** | $S_0$-Min | 0.00 | 1.452 | 18.9 | 1.453 | 19.4 | | | | | 2.8 |
| | $S_1$-Min | 3.21 | 1.419 | 11.5 | 1.423 | 12.9 | | | | | 14.2 |
| **25-cal** | $S_0$-Min | 0.00 | 1.452 | 19.2 | 1.452 | 19.8 | 1.452 | 19.8 | | | 3.5 |
| | $S_1$-Min, Ex1 | 3.15 | 1.422 | 12.3 | 1.424 | 12.3 | 1.445 | 18.4 | | | 21.9 |
| | $S_1$-Min, Ex2 | 3.23 | 1.447 | 21.3 | 1.413 | 13.1 | 1.429 | 15.5 | | | 7.8 |
| | $S_1$-Min, Ex3 | 3.22 | 1.445 | 17.1 | 1.426 | 15.5 | 1.419 | 13.4 | | | 8.8 |
| **28-cal** | $S_0$-Min | 0.00 | 1.452 | 18.9 | 1.453 | 19.0 | 1.452 | 17.8 | 1.453 | 20.0 | 4.4 |
| | $S_1$-Min, Ex1 | 3.15 | 1.423 | 12.2 | 1.425 | 12.2 | 1.443 | 16.6 | 1.451 | 20.2 | 26.1 |
| | $S_1$-Min, Ex2 | 3.21 | 1.447 | 22.0 | 1.417 | 12.8 | 1.427 | 13.6 | 1.448 | 19.3 | 17.0 |
| | $S_1$-Min, Ex3 | 3.21 | 1.448 | 18.2 | 1.430 | 16.3 | 1.416 | 13.3 | 1.448 | 17.8 | 2.5 |

[a]Energy of $S_1$ minima corresponds to adiabatic excitation energy. [b]See Figure 6 for labeling of the fragments. [c]Dihedral angle defined by $N_1$, $C_2$, $C_{7'}$ and $C_{6'}$ atoms of connected rings.

**Table S8**. Relative energy,[a] calculated interfragment $C_2$-$C_{7'}$ distance,[b] and rotational dihedral angle around the $C_2$-$C_{7'}$ bond,[b,c] for the ground and excited state minima in acetonitrile.

| | | $E_{rel}$ (eV) | $r_{AB}$ (Å) | $\varphi_{AB}$ (°) | $r_{BC}$ (Å) | $\varphi_{BC}$ (°) | $r_{CD}$ (Å) | $\varphi_{CD}$ (°) | $r_{DE}$ (Å) | $\varphi_{DE}$ (°) |
|---|---|---|---|---|---|---|---|---|---|---|
| **13-cal** | $S_0$-Min | 0.00 | 1.454 | 18.4 | | | | | | |
| | $S_1$-Min | 3.23 | 1.400 | 9.1 | | | | | | |
| **22-cal** | $S_0$-Min | 0.00 | 1.454 | 19.1 | 1.454 | 18.2 | | | | |
| | $S_1$-Min, Ex1 | 3.11 | 1.409 | 10.5 | 1.435 | 14.7 | | | | |
| | $S_1$-Min, Ex2 | 3.17 | 1.441 | 18.4 | 1.405 | 11.8 | | | | |
| **25-cal** | $S_0$-Min | 0.00 | 1.454 | 21.8 | 1.454 | 19.9 | 1.454 | 20.8 | | |
| | $S_1$-Min, Ex1 | 3.08 | 1.412 | 10.9 | 1.433 | 14.3 | 1.4509 | 20.3 | | |
| | $S_1$-Min, Ex2 | 3.12 | 1.447 | 22.2 | 1.407 | 12.0 | 1.443 | 16.9 | | |
| | $S_1$-Min, Ex3 | 3.14 | 1.452 | 20.4 | 1.435 | 17.6 | 1.411 | 11.6 | | |
| **28-cal** | $S_0$-Min | 0.00 | 1.454 | 20.7 | 1.454 | 18.4 | 1.454 | 20.8 | 1.454 | 19.9 |
| | $S_1$-Min, Ex1 | 3.08 | 1.413 | 12.6 | 1.433 | 14.9 | 1.449 | 18.1 | 1.454 | 19.2 |
| | $S_1$-Min, Ex2 | 3.10 | 1.448 | 23.2 | 1.410 | 10.6 | 1.439 | 15.9 | 1.453 | 19.4 |
| | $S_1$-Min, Ex3 | 3.10 | 1.453 | 20.1 | 1.443 | 19.5 | 1.408 | 12.3 | 1.446 | 16.9 |

[a]Energy of $S_1$ minima corresponds to adiabatic excitation energy. [b]See Figure 6 for labeling of the fragments. [c]Dihedral angle defined by $N_1$, $C_2$, $C_{7'}$ and $C_{6'}$ atoms of connected rings.

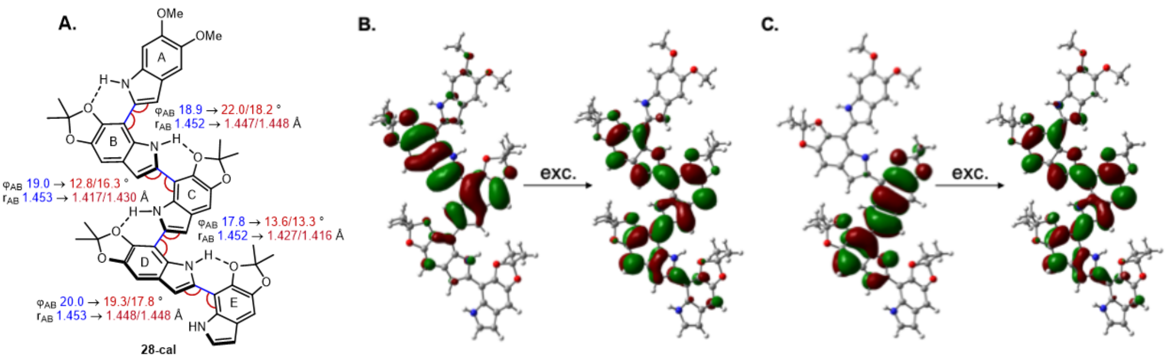


**Figure S7**. Analysis of excited states of pentamer **28-cal**. (A) Geometric changes induced by excitation. The distance and rotational angle between fragments are displayed in blue for the ground state and in red for the Ex2 and Ex3 excited states. (B) Natural transition orbitals for Ex2. (C) Natural transition orbitals for Ex3.

## 7. Reference:


1. X. Wang, L. Kinziabulatova, M. Bortoli, A. Manickoth, M. A. Barilla, H. Huang, L. Blancafort, B. Kohler, J.-P. Lumb, *Nat. Chem.* 2023, **15**, 787-793.
2. H. Meier, U. Stalmach, H. Kolshorn, *Acta Polym.* 1997, **48**, 379-384.
3. A. V. Marenich, C. J. Cramer, D. G. Truhlar, *J. of Phys. Chem. B* 2009, **113**, 6378-6396.
4. (a) M. K. Kesharwani, B. Brauer, J. M. L. Martin, *J. of Phys. Chem. A* 2015, **119**, 1701-1714; (b) M. L. Laury, M. J. Carlson, A. K. Wilson, *J. Comput. Chem.* 2012, **33**, 2380-2387.
5. J. M. Herbert, in *Theoretical and Computational Photochemistry* (Eds.: C. García-Iriepa, M. Marazzi), Elsevier, 2023, pp. 69-118.


## 8. $^{1}$H and $^{13}$C NMR Spectra

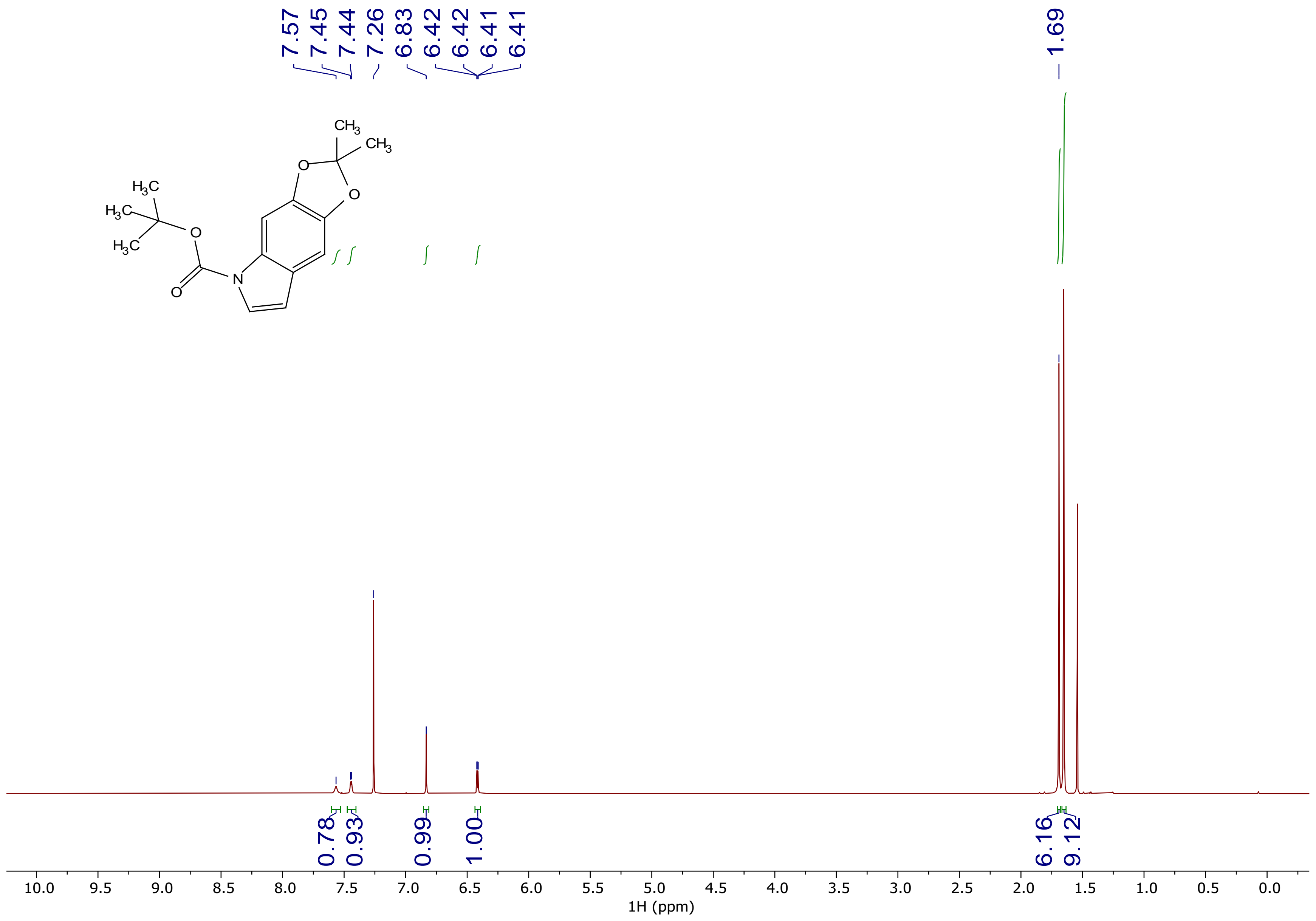
7.57
7.45
7.44
7.26
6.83
6.42
6.42
6.41
6.41
1.69
CH3
CH3
O
O
H3C
H3C
H3C
O
N
O
0.78
0.93
0.99
1.00
6.16
9.12
10.0 9.5 9.0 8.5 8.0 7.5 7.0 6.5 6.0 5.5 5.0 4.5 4.0 3.5 3.0 2.5 2.0 1.5 1.0 0.5 0.0
1H (ppm)

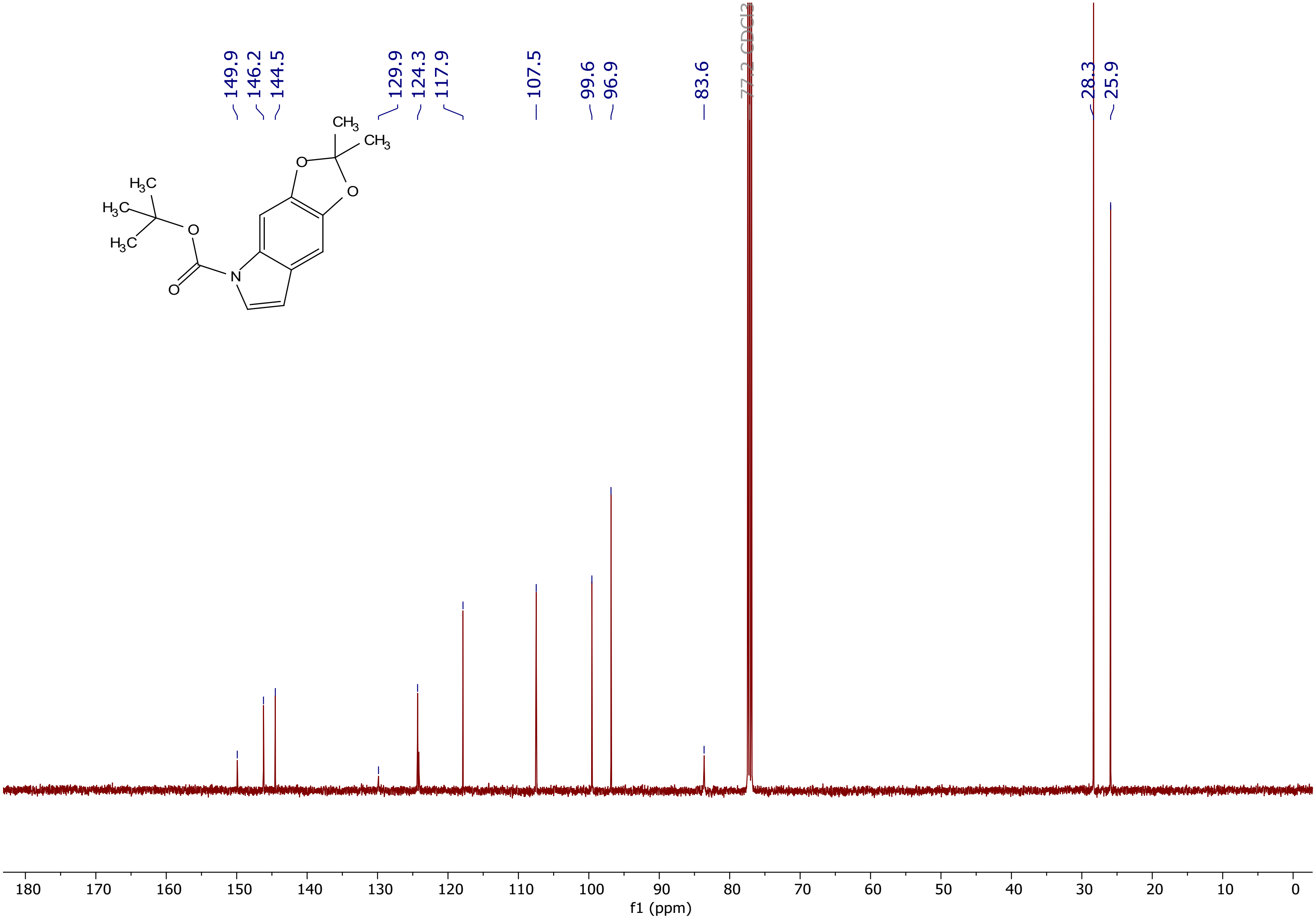
149.9
146.2
144.5
129.9
124.3
117.9
107.5
99.6
96.9
83.6
77.2 CDCl3
28.3
25.9
CH3
CH3
O
O
H3C
H3C
H3C
O
N
O
180
170
160
150
140
130
120
110
100
90
80
70
60
50
40
30
20
10
0
f1 (ppm)

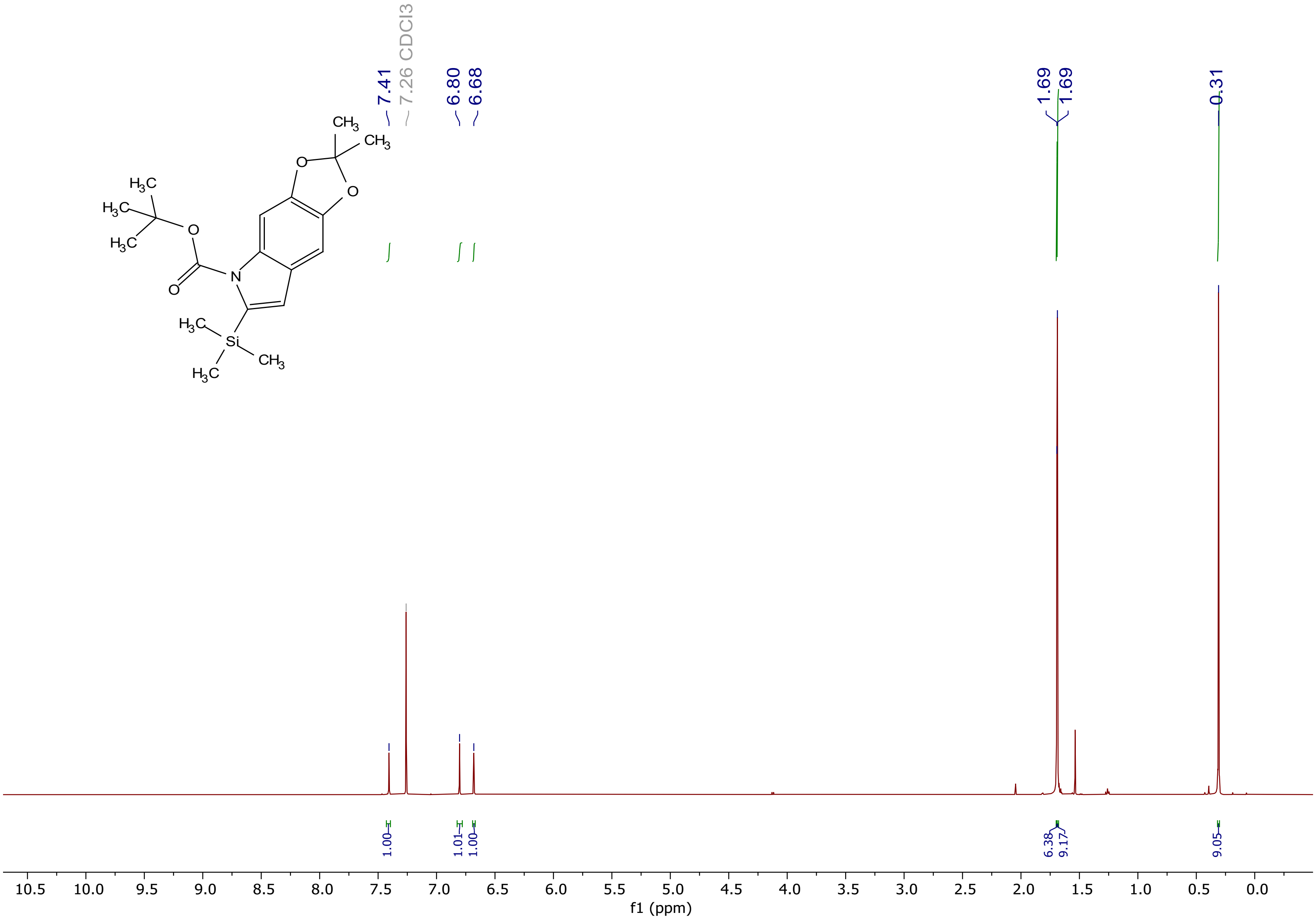

7.41
7.26 CDCl3
6.80
6.68
1.69
1.69
0.31
1.00
1.01
1.00
6.38
9.17
9.05
10.5 10.0 9.5 9.0 8.5 8.0 7.5 7.0 6.5 6.0 5.5 5.0 4.5 4.0 3.5 3.0 2.5 2.0 1.5 1.0 0.5 0.0
f1 (ppm)

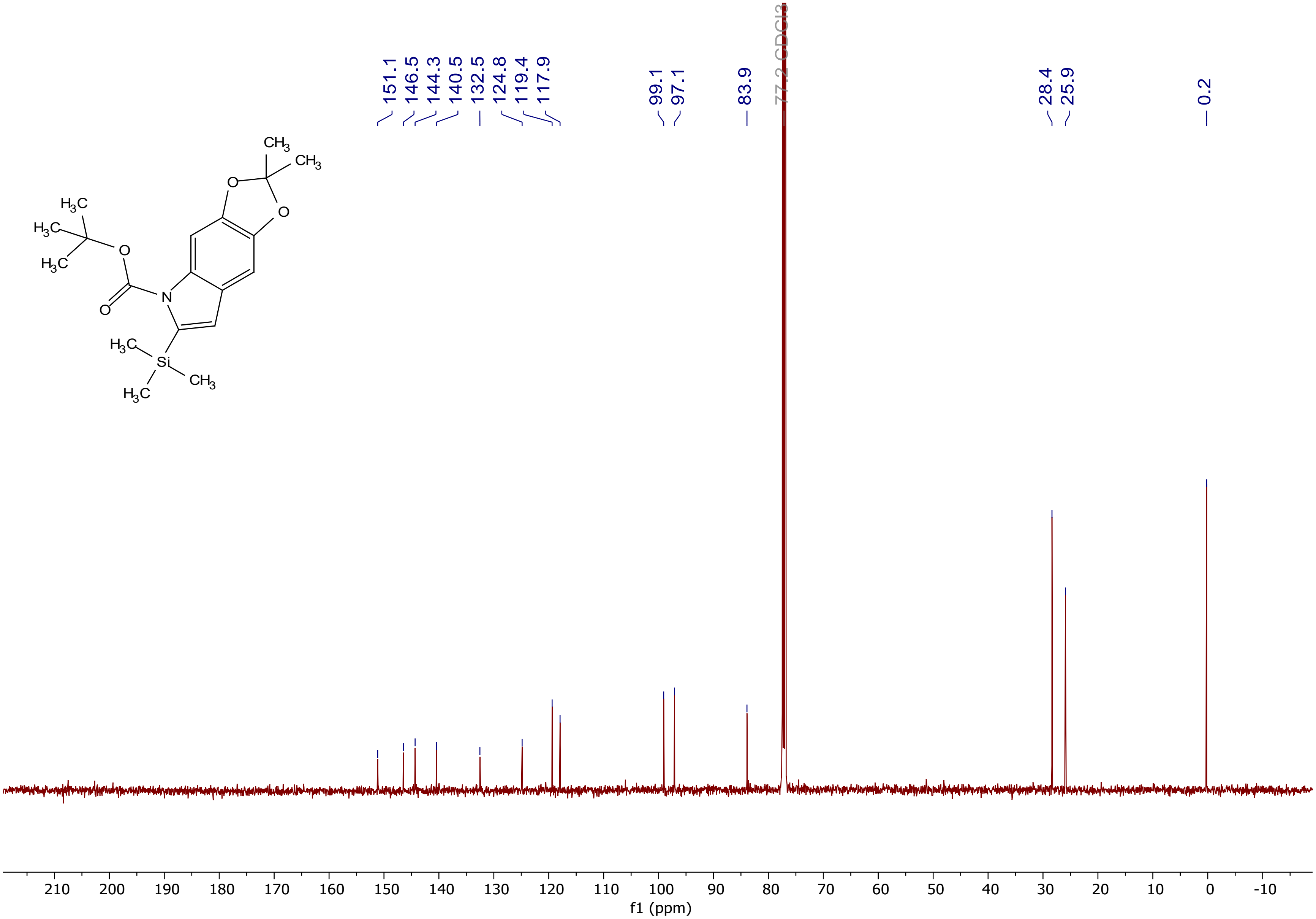
151.1
146.5
144.3
140.5
132.5
124.8
119.4
117.9
99.1
97.1
83.9
77.2 CDCl3
28.4
25.9
0.2
f1 (ppm)

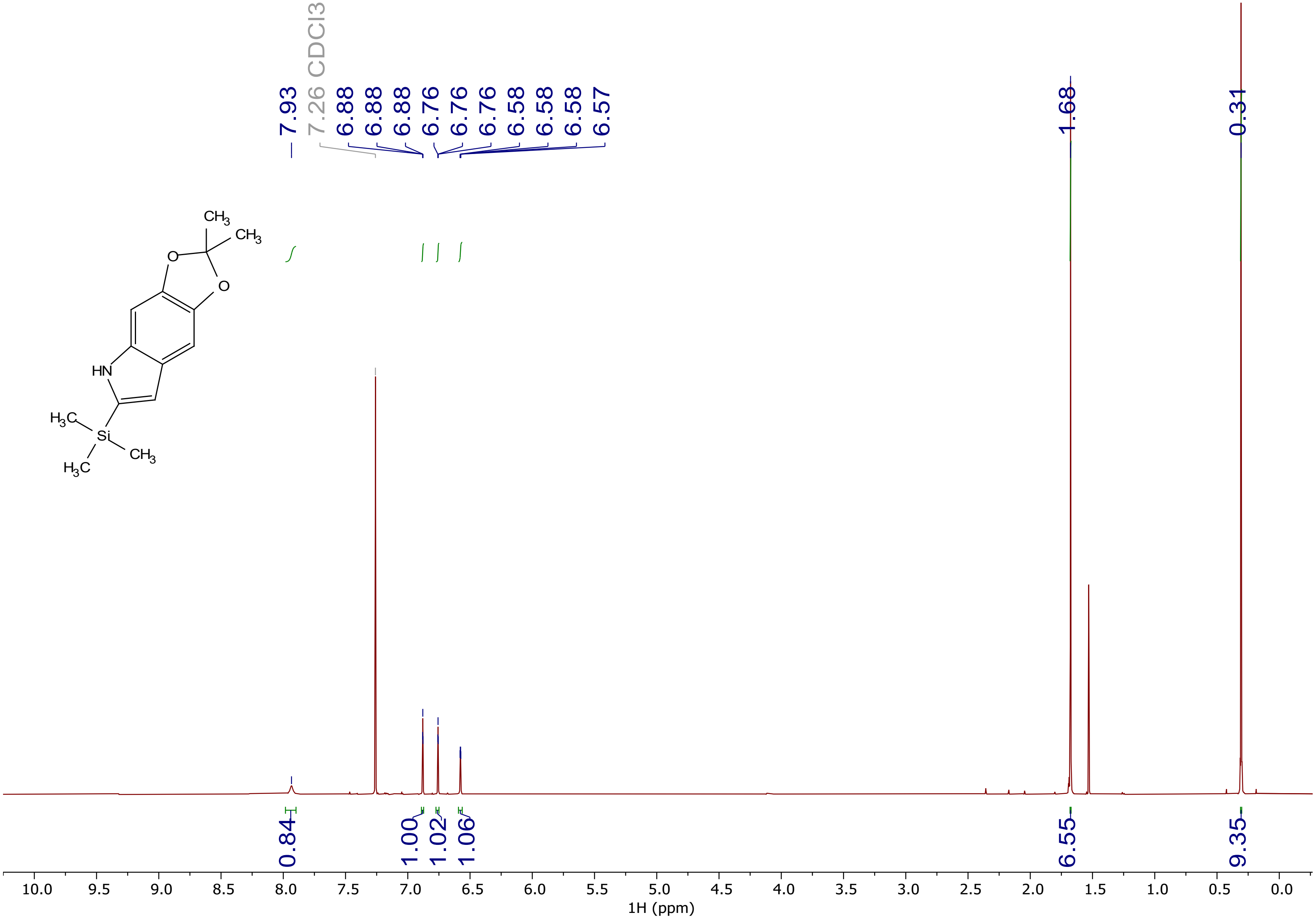

7.93
7.26 CDCl3
6.88
6.88
6.88
6.76
6.76
6.76
6.58
6.58
6.58
6.57
1.68
0.31
0.84
1.00
1.02
1.06
6.55
9.35
10.0
9.5
9.0
8.5
8.0
7.5
7.0
6.5
6.0
5.5
5.0
4.5
4.0
3.5
3.0
2.5
2.0
1.5
1.0
0.5
0.0
1H (ppm)
CH3
CH3
O
O
HN
H3C
Si
CH3
H3C

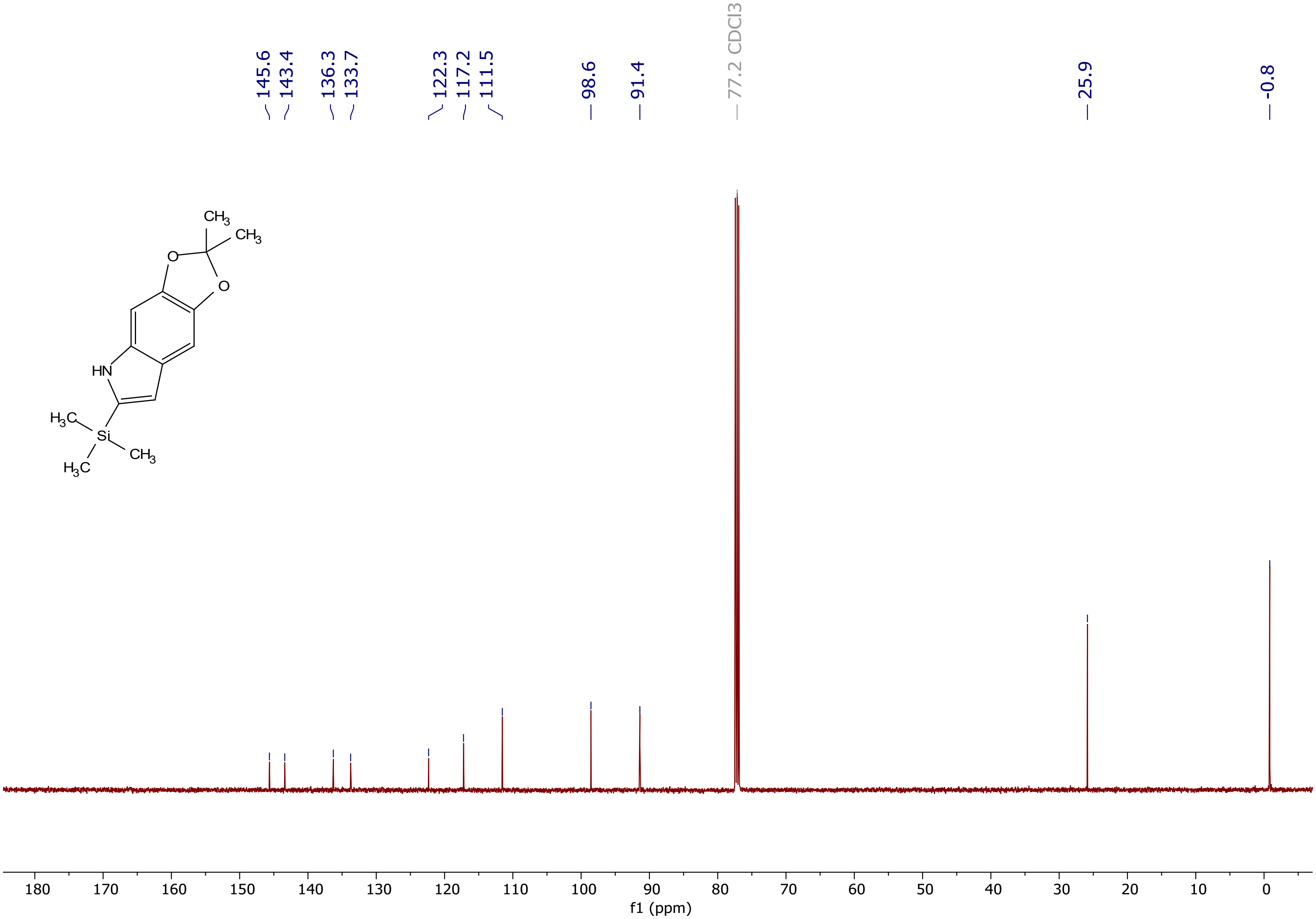

145.6
143.4
136.3
133.7
122.3
117.2
111.5
98.6
91.4
77.2 CDCl3
25.9
-0.8
180
170
160
150
140
130
120
110
100
90
80
70
60
50
40
30
20
10
0
f1 (ppm)

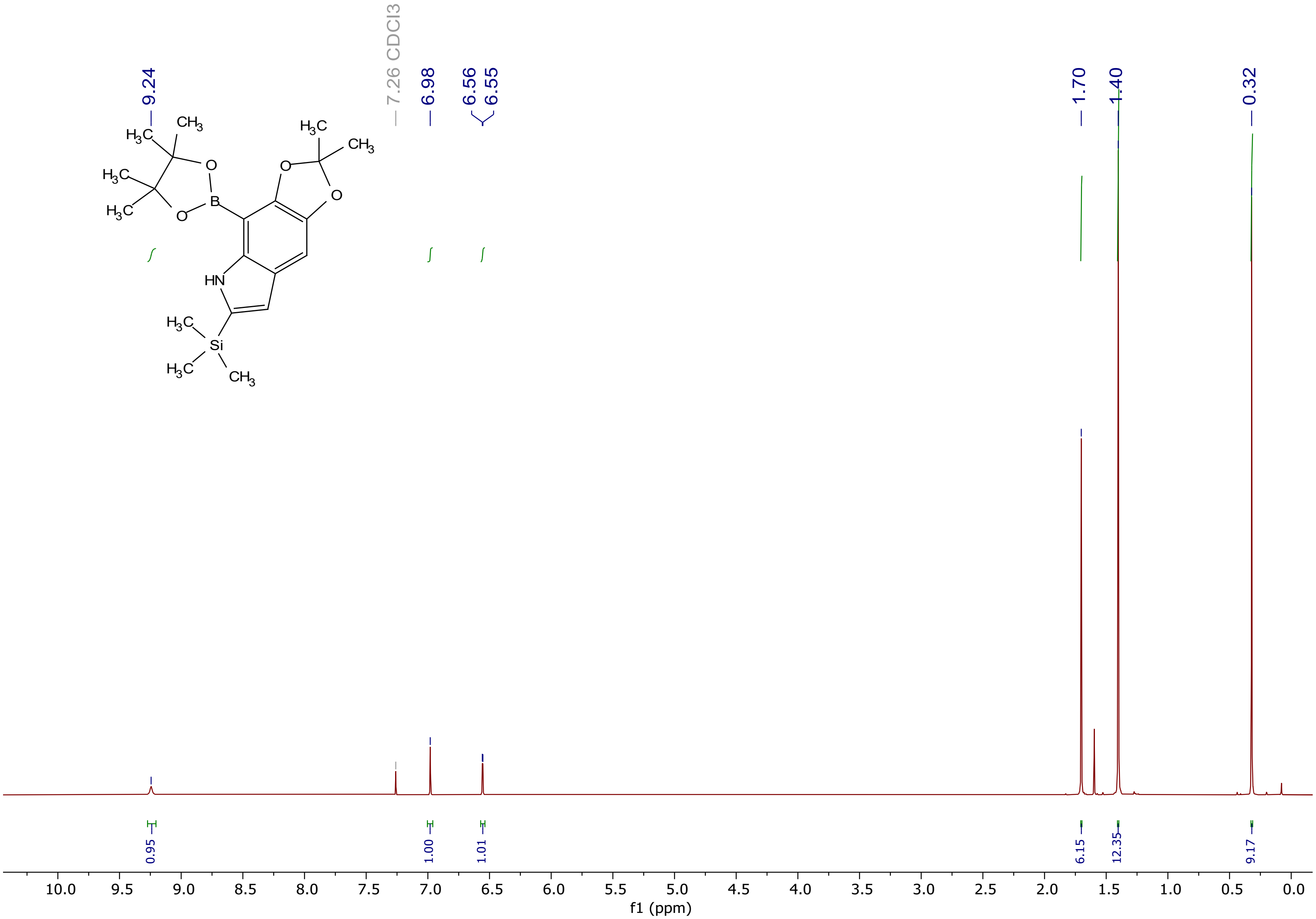

9.24
7.26 CDCl3
6.98
6.56
6.55
1.70
1.40
0.32
0.95
1.00
1.01
6.15
12.35
9.17
10.0
9.5
9.0
8.5
8.0
7.5
7.0
6.5
6.0
5.5
5.0
4.5
4.0
3.5
3.0
2.5
2.0
1.5
1.0
0.5
0.0
f1 (ppm)

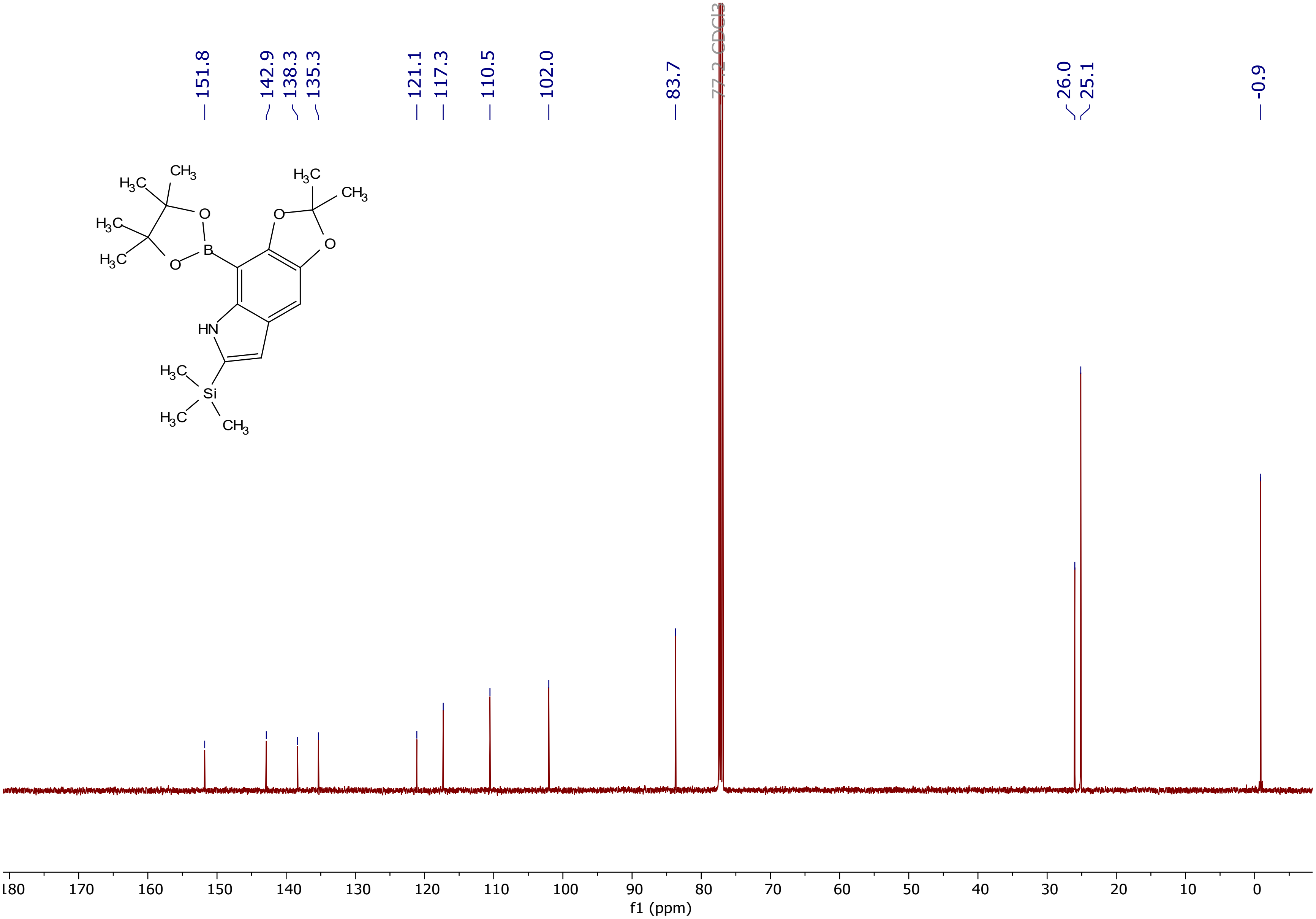
151.8
142.9
138.3
135.3
121.1
117.3
110.5
102.0
83.7
77.2 CDCl3
26.0
25.1
-0.9
CH3
H3C
O
B
HN
Si
180
170
160
150
140
130
120
110
100
90
80
70
60
50
40
30
20
10
0
f1 (ppm)

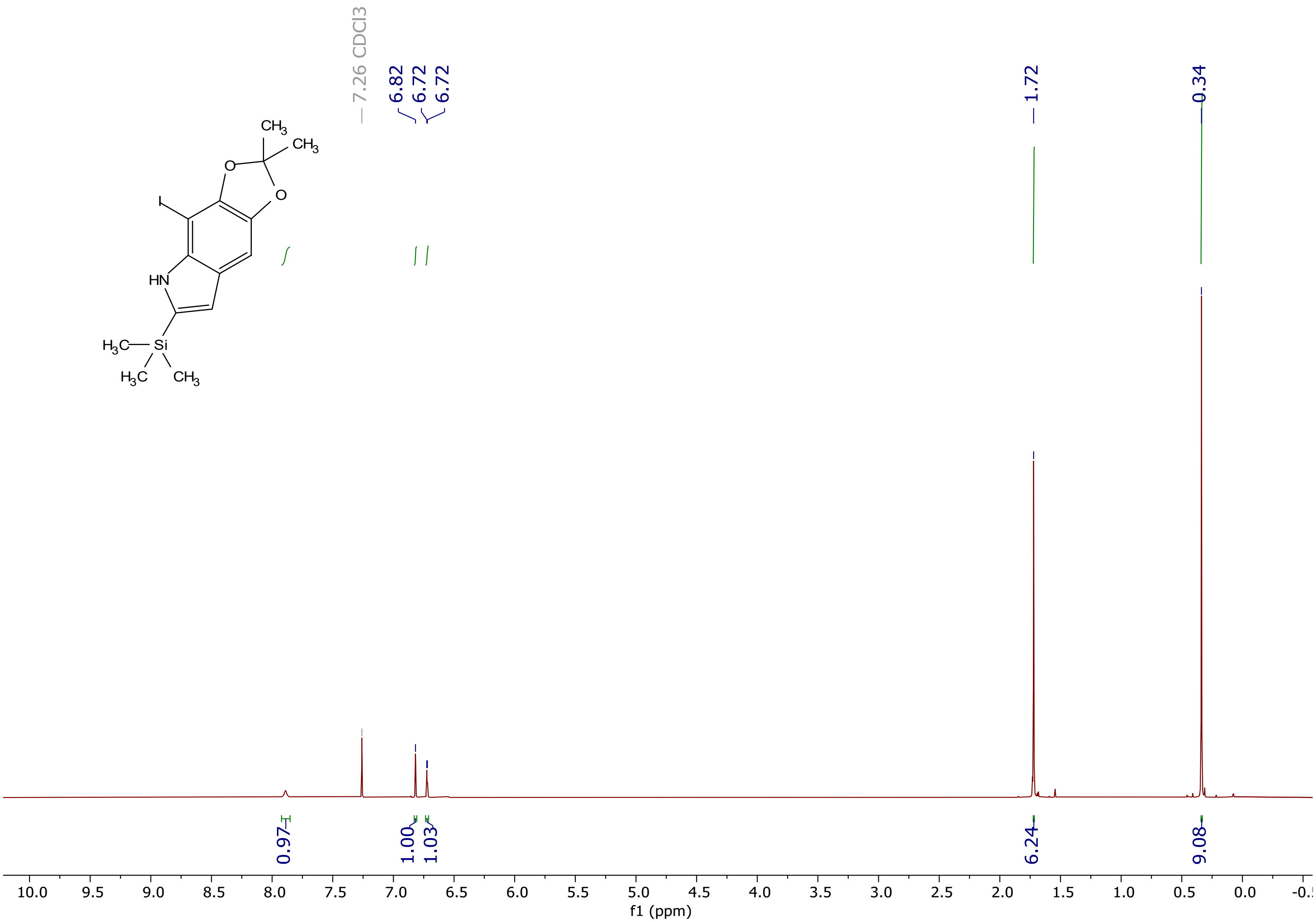

7.26 CDCl3
6.82
6.72
6.72
1.72
0.34
CH3
CH3
O
O
I
HN
H3C
Si
H3C
CH3
0.97
1.00
1.03
6.24
9.08
10.0 9.5 9.0 8.5 8.0 7.5 7.0 6.5 6.0 5.5 5.0 4.5 4.0 3.5 3.0 2.5 2.0 1.5 1.0 0.5 0.0
f1 (ppm)

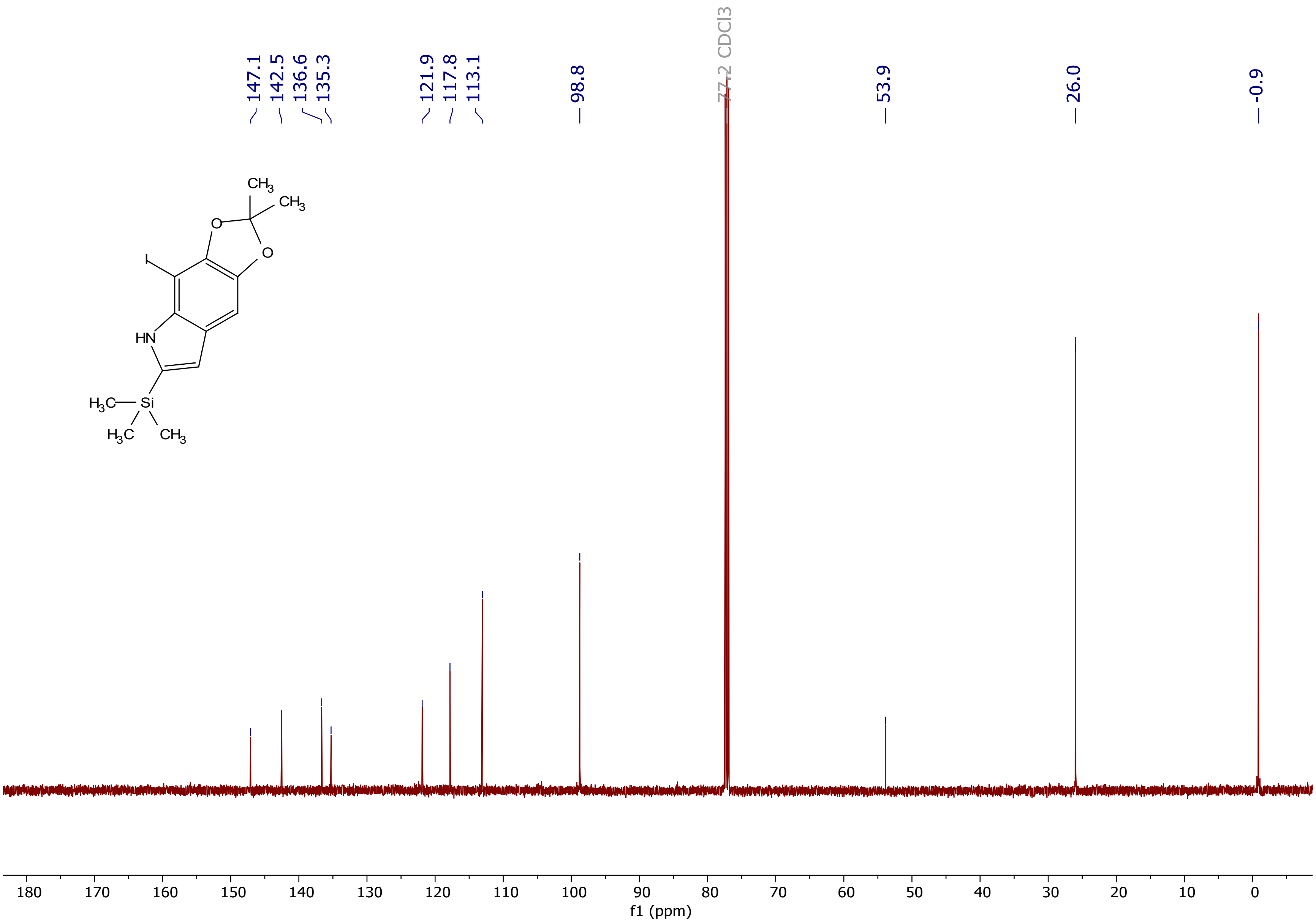
147.1
142.5
136.6
135.3
121.9
117.8
113.1
98.8
77.2 CDCl3
53.9
26.0
-0.9
CH3
CH3
O
O
I
HN
H3C
Si
H3C
CH3
180
170
160
150
140
130
120
110
100
90
80
70
60
50
40
30
20
10
0
f1 (ppm)

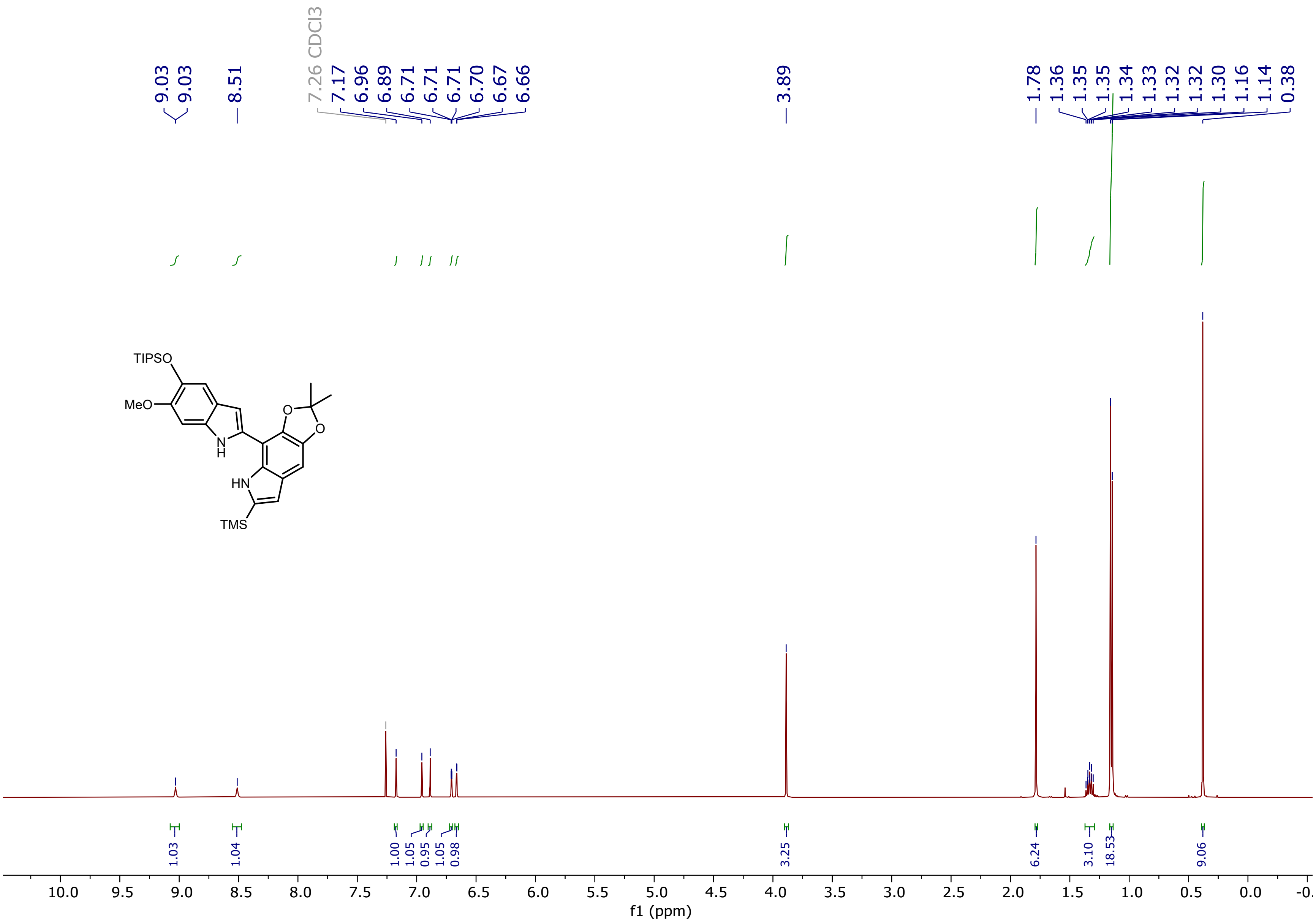

9.03
9.03
8.51
7.26 CDCl3
7.17
6.96
6.89
6.71
6.71
6.71
6.70
6.67
6.66
3.89
1.78
1.36
1.35
1.35
1.34
1.33
1.32
1.32
1.30
1.16
1.14
0.38
TIPSO
MeO
N
H
O
O
HN
TMS
1.03
1.04
1.00
1.05
0.95
1.05
0.98
3.25
6.24
3.10
18.53
9.06
f1 (ppm)

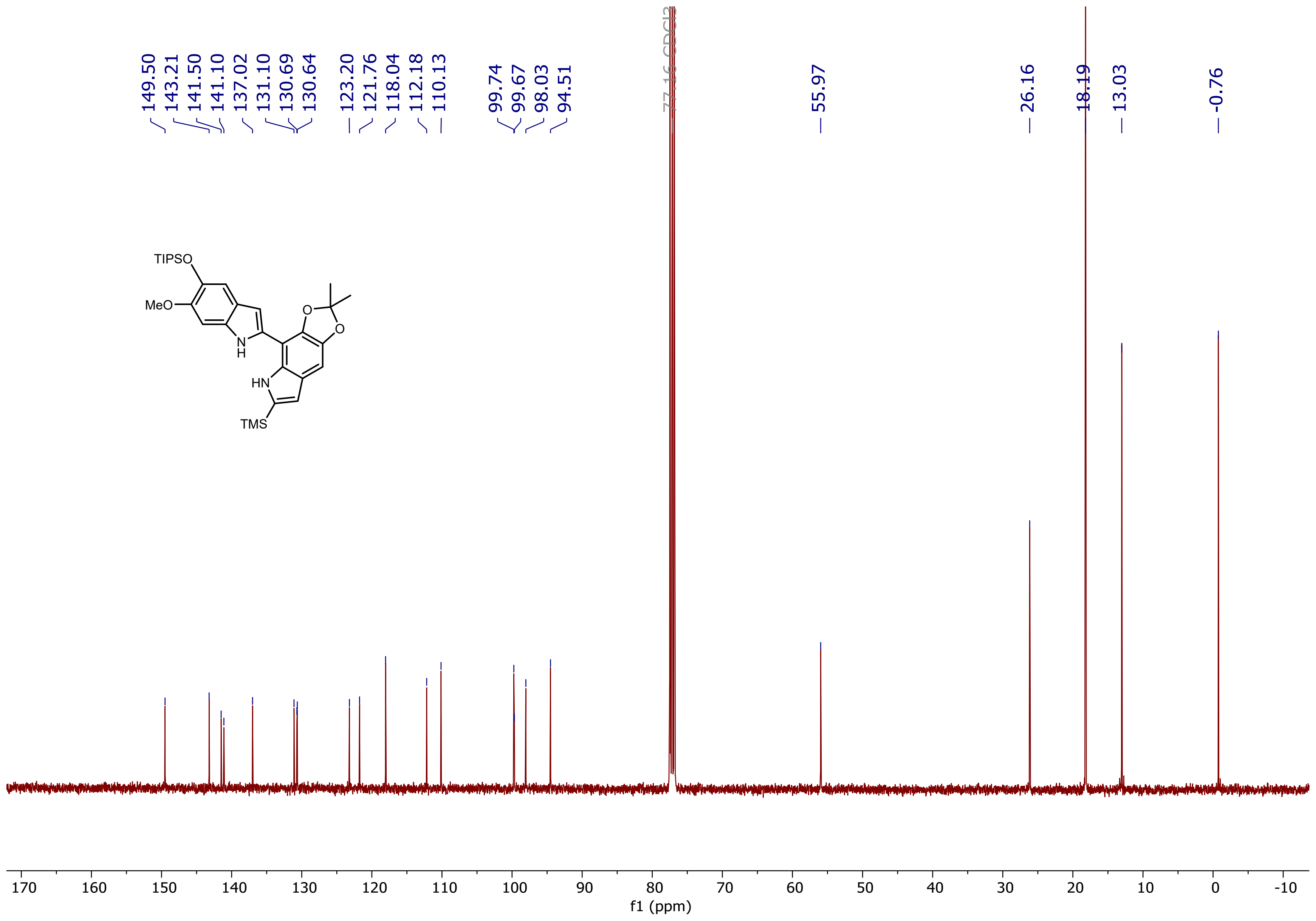

TIPSO
MeO
N
H
O
O
HN
TMS
149.50
143.21
141.50
141.10
137.02
131.10
130.69
130.64
123.20
121.76
118.04
112.18
110.13
99.74
99.67
98.03
94.51
77.16 CDCl3
55.97
26.16
18.19
13.03
-0.76
170
160
150
140
130
120
110
100
90
80
70
60
50
40
30
20
10
0
-10
f1 (ppm)

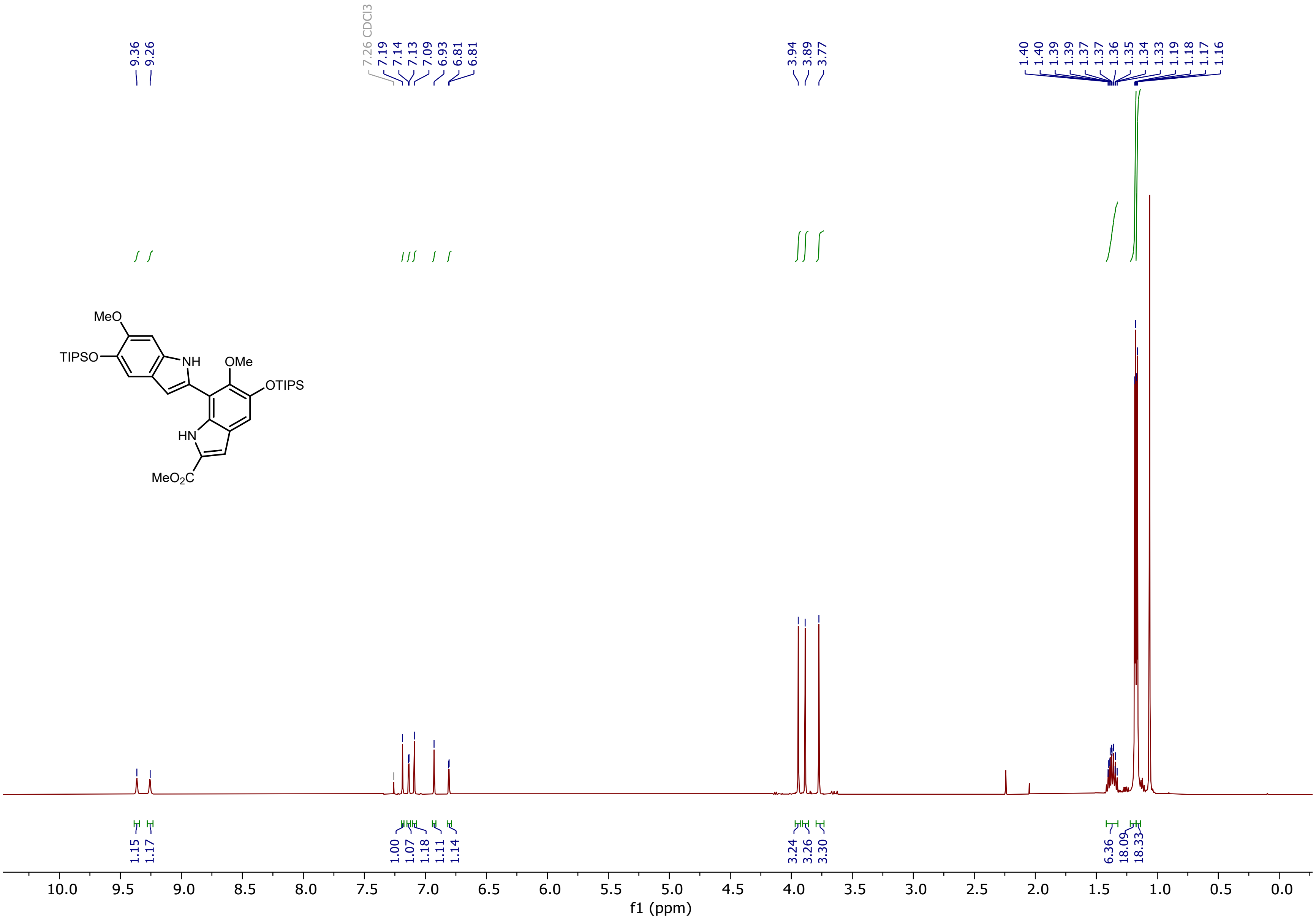
9.36
9.26
7.26 CDCl3
7.19
7.14
7.13
7.09
6.93
6.81
6.81
3.94
3.89
3.77
1.40
1.40
1.39
1.39
1.37
1.37
1.36
1.35
1.34
1.33
1.19
1.18
1.17
1.16
MeO
TIPSO
NH
OMe
OTIPS
HN
MeO2C
1.15
1.17
1.00
1.07
1.18
1.11
1.14
3.24
3.26
3.30
6.36
18.09
18.33
10.0
9.5
9.0
8.5
8.0
7.5
7.0
6.5
6.0
5.5
5.0
4.5
4.0
3.5
3.0
2.5
2.0
1.5
1.0
0.5
0.0
f1 (ppm)

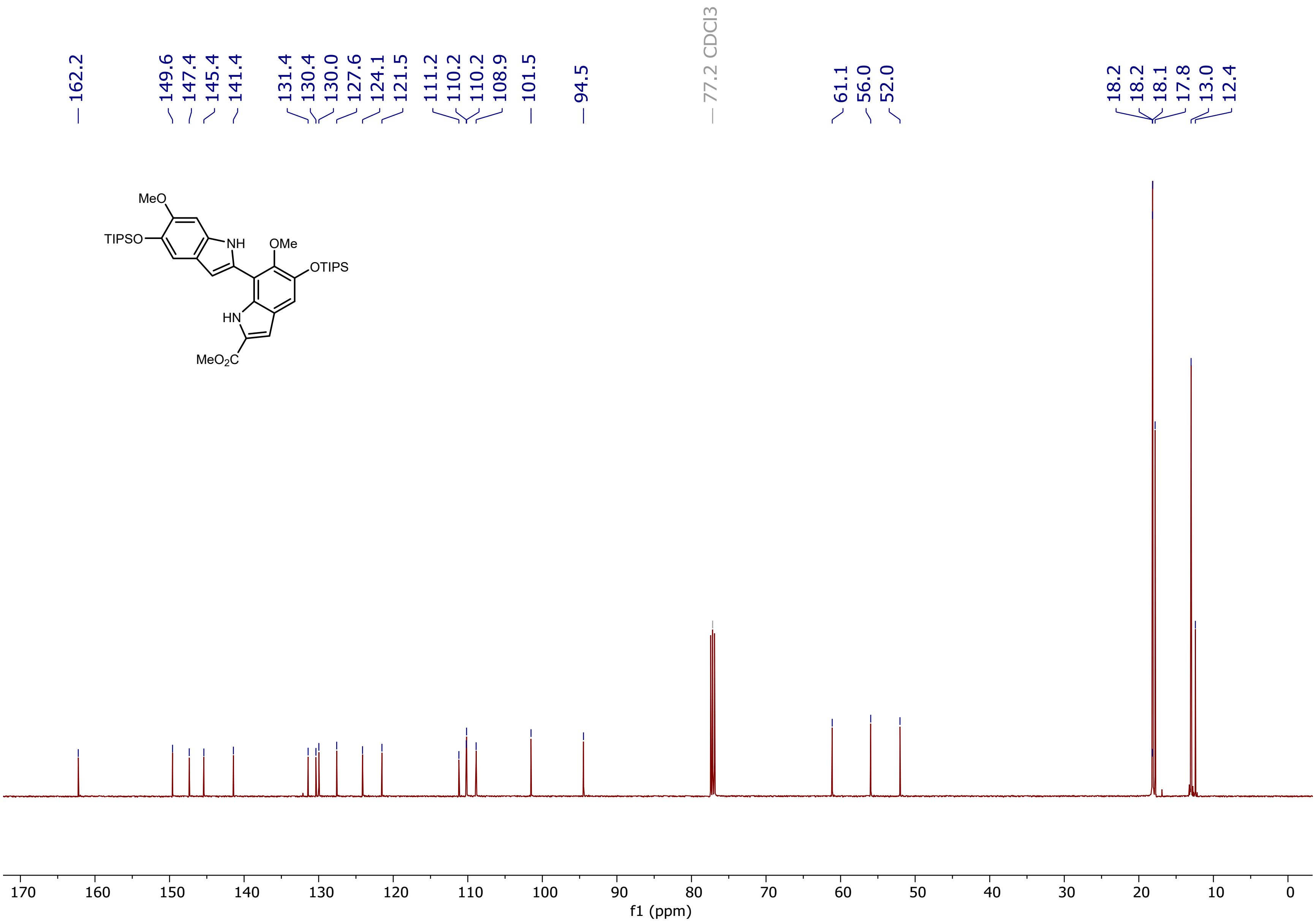
162.2
149.6
147.4
145.4
141.4
131.4
130.4
130.0
127.6
124.1
121.5
111.2
110.2
110.2
108.9
101.5
94.5
77.2 CDCl3
61.1
56.0
52.0
18.2
18.2
18.1
17.8
13.0
12.4
MeO
TIPSO
NH
OMe
OTIPS
HN
MeO2C
170
160
150
140
130
120
110
100
90
80
70
60
50
40
30
20
10
0
f1 (ppm)

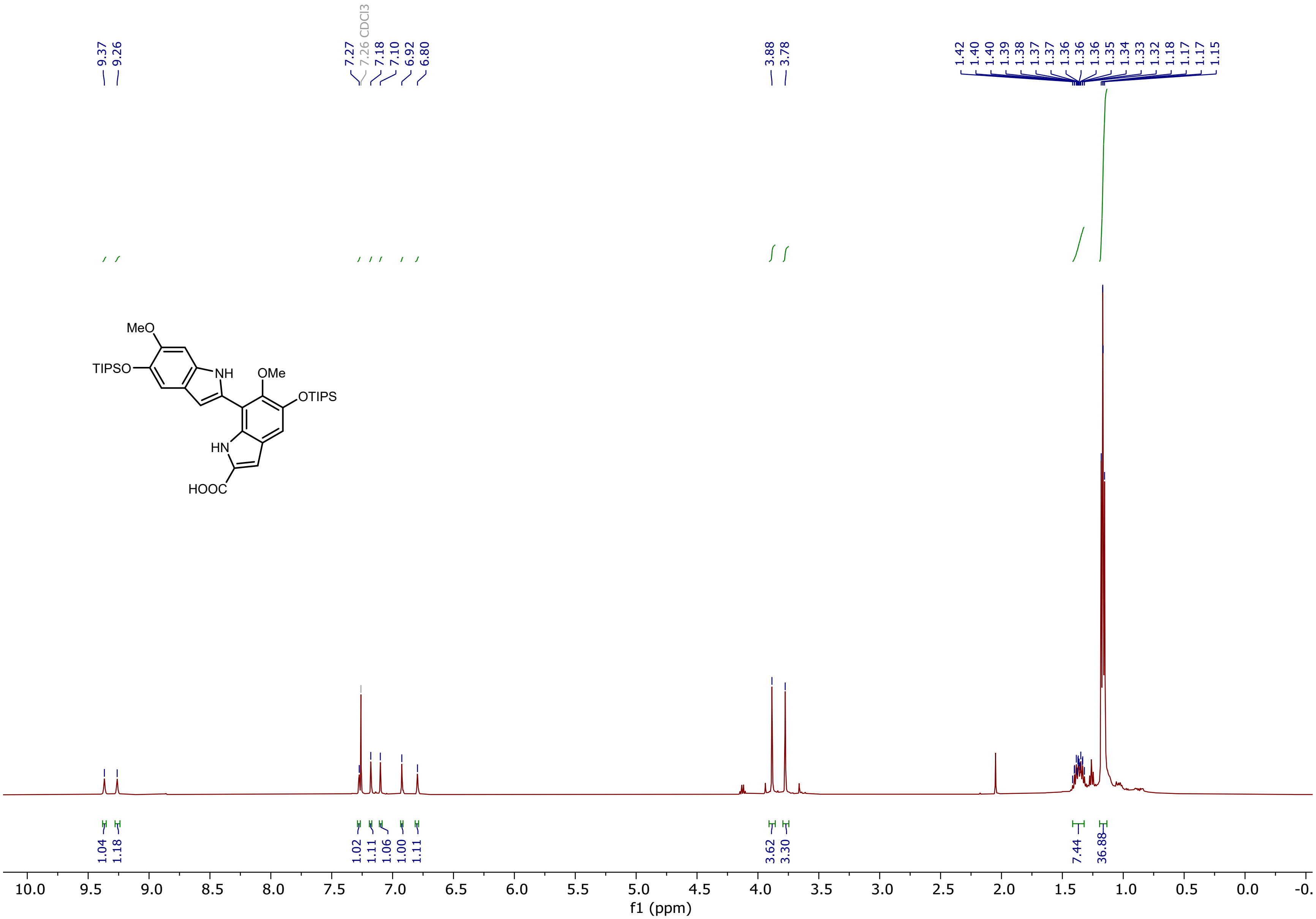
9.37
9.26
7.27
7.26 CDCl3
7.18
7.10
6.92
6.80
3.88
3.78
1.42
1.40
1.40
1.39
1.38
1.37
1.37
1.36
1.36
1.36
1.35
1.34
1.33
1.32
1.18
1.17
1.17
1.15
MeO
TIPSO
NH
OMe
OTIPS
HN
HOOC
1.04
1.18
1.02
1.11
1.06
1.00
1.11
3.62
3.30
7.44
36.88
10.0
9.5
9.0
8.5
8.0
7.5
7.0
6.5
6.0
5.5
5.0
4.5
4.0
3.5
3.0
2.5
2.0
1.5
1.0
0.5
0.0
f1 (ppm)

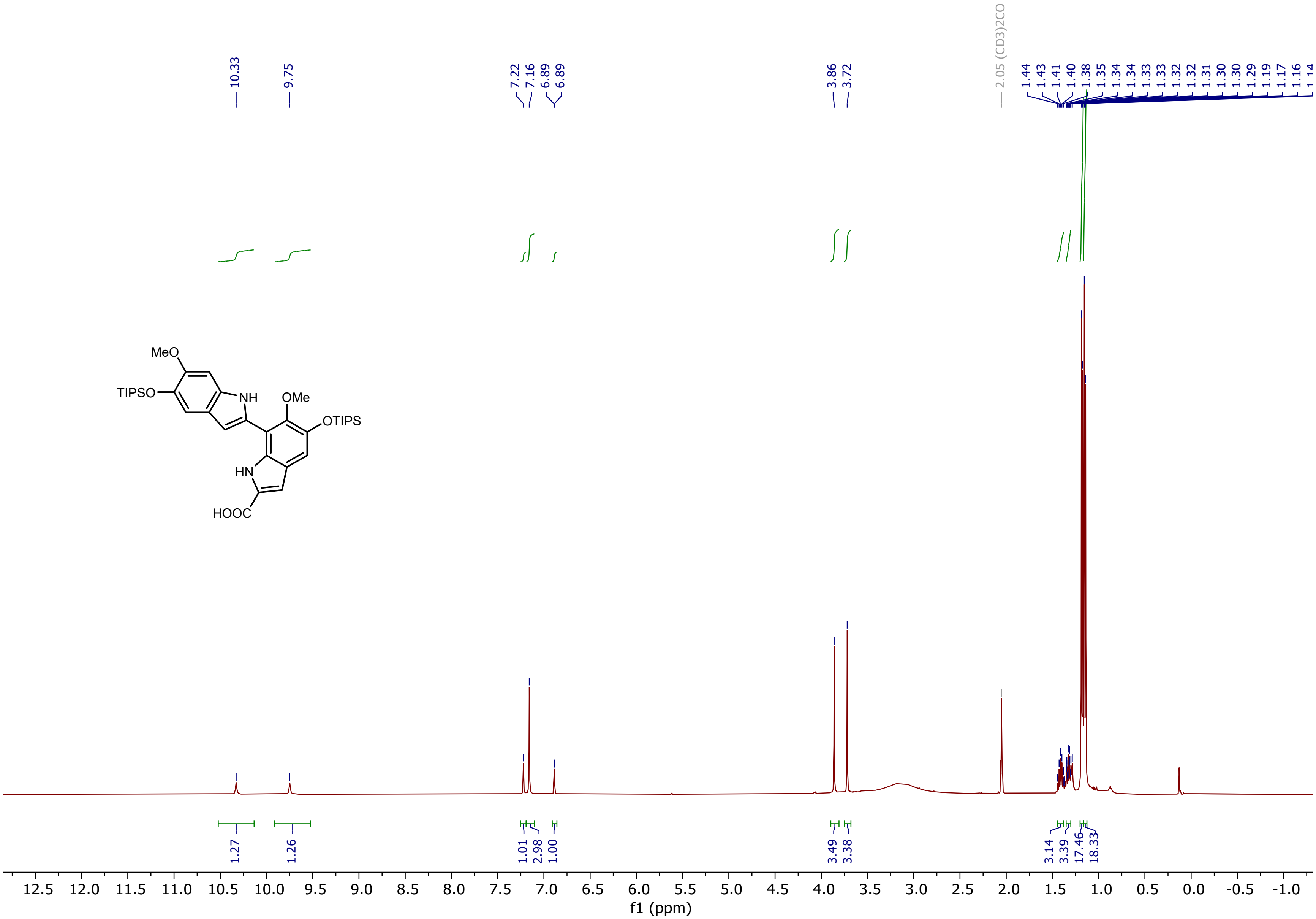
10.33
9.75
7.22
7.16
6.89
6.89
3.86
3.72
2.05 (CD3)2CO
1.44
1.43
1.41
1.40
1.38
1.35
1.34
1.34
1.33
1.33
1.32
1.32
1.31
1.30
1.30
1.29
1.19
1.17
1.16
MeO
TIPSO
NH
OMe
OTIPS
HN
HOOC
1.27
1.26
1.01
2.98
1.00
3.49
3.38
3.14
3.39
17.46
18.33
12.5 12.0 11.5 11.0 10.5 10.0 9.5 9.0 8.5 8.0 7.5 7.0 6.5 6.0 5.5 5.0 4.5 4.0 3.5 3.0 2.5 2.0 1.5 1.0 0.5 0.0 -0.5 -1.0
f1 (ppm)

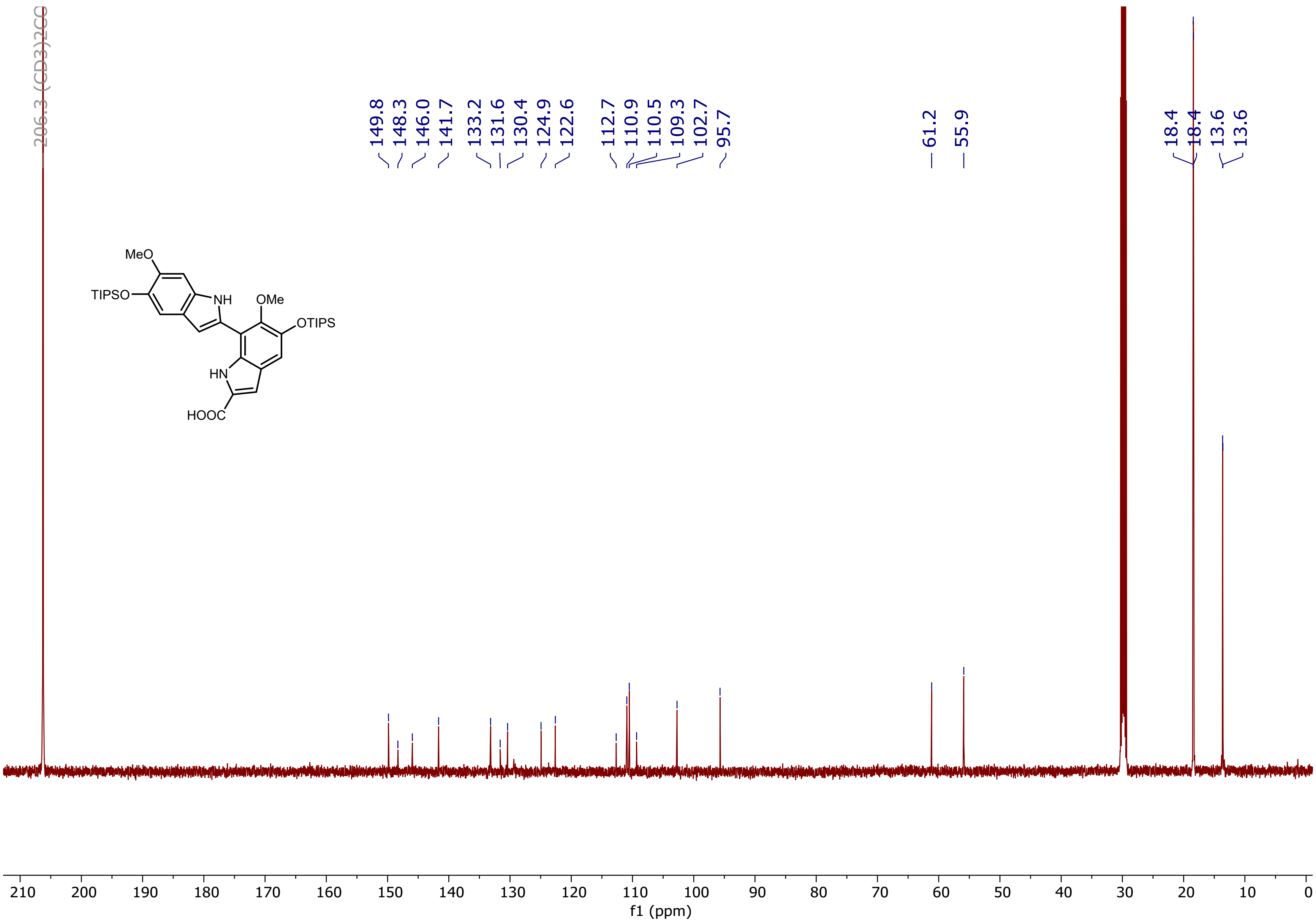
206.3 (CD3)2CO
149.8
148.3
146.0
141.7
133.2
131.6
130.4
124.9
122.6
112.7
110.9
110.5
109.3
102.7
95.7
61.2
55.9
18.4
18.4
13.6
13.6
MeO
TIPSO
NH
OMe
OTIPS
HN
HOOC
210
200
190
180
170
160
150
140
130
120
110
100
90
80
70
60
50
40
30
20
10
0
f1 (ppm)

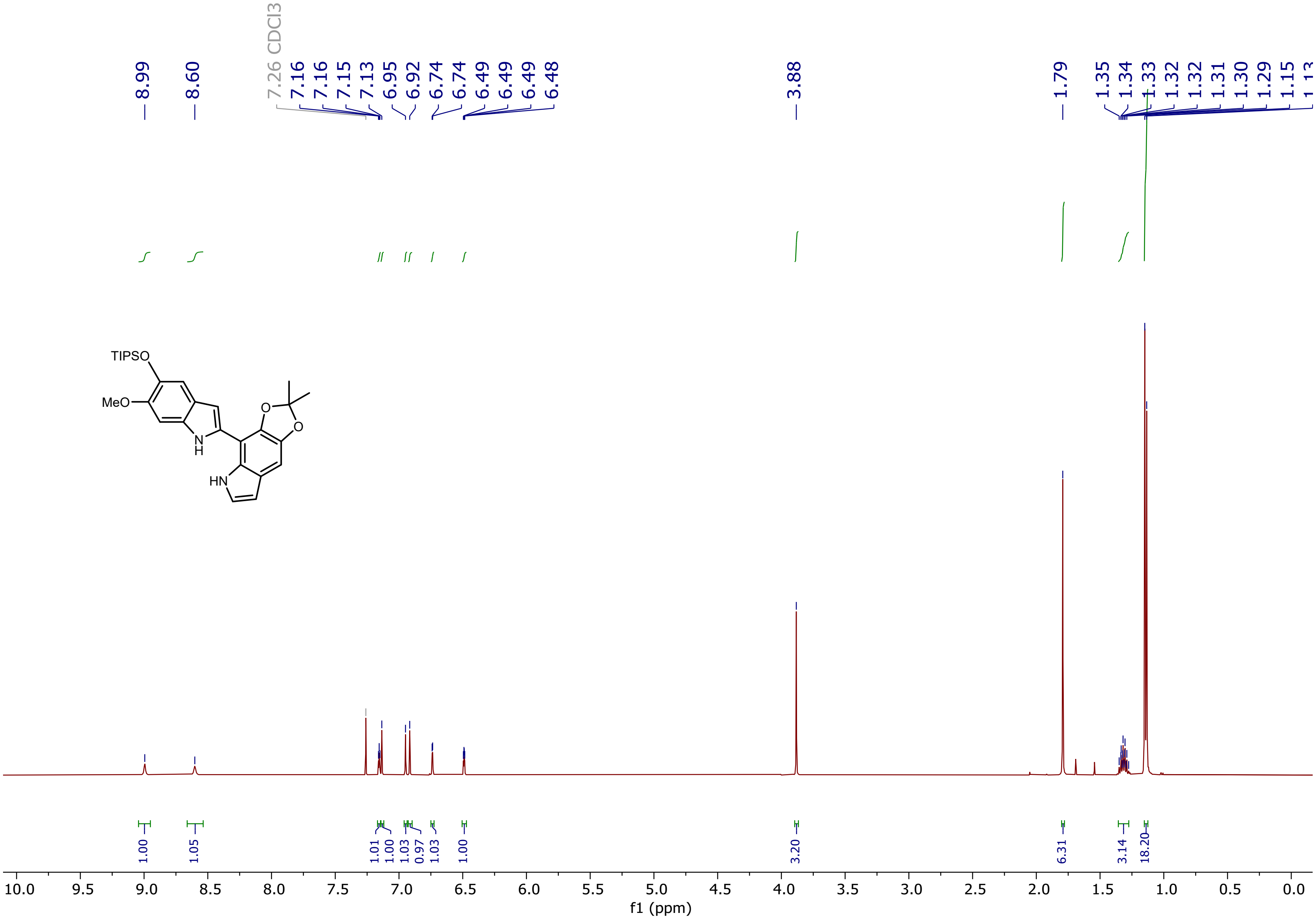
8.99
8.60
7.26 CDCl3
7.16
7.16
7.15
7.13
6.95
6.92
6.74
6.74
6.49
6.49
6.49
6.48
3.88
1.79
1.35
1.34
1.33
1.32
1.32
1.31
1.30
1.29
1.15
TIPSO
MeO
N
H
HN
O
O
1.00
1.05
1.01
1.00
1.03
0.97
1.03
1.00
3.20
6.31
3.14
18.20
10.0
9.5
9.0
8.5
8.0
7.5
7.0
6.5
6.0
5.5
5.0
4.5
4.0
3.5
3.0
2.5
2.0
1.5
1.0
0.5
0.0
f1 (ppm)

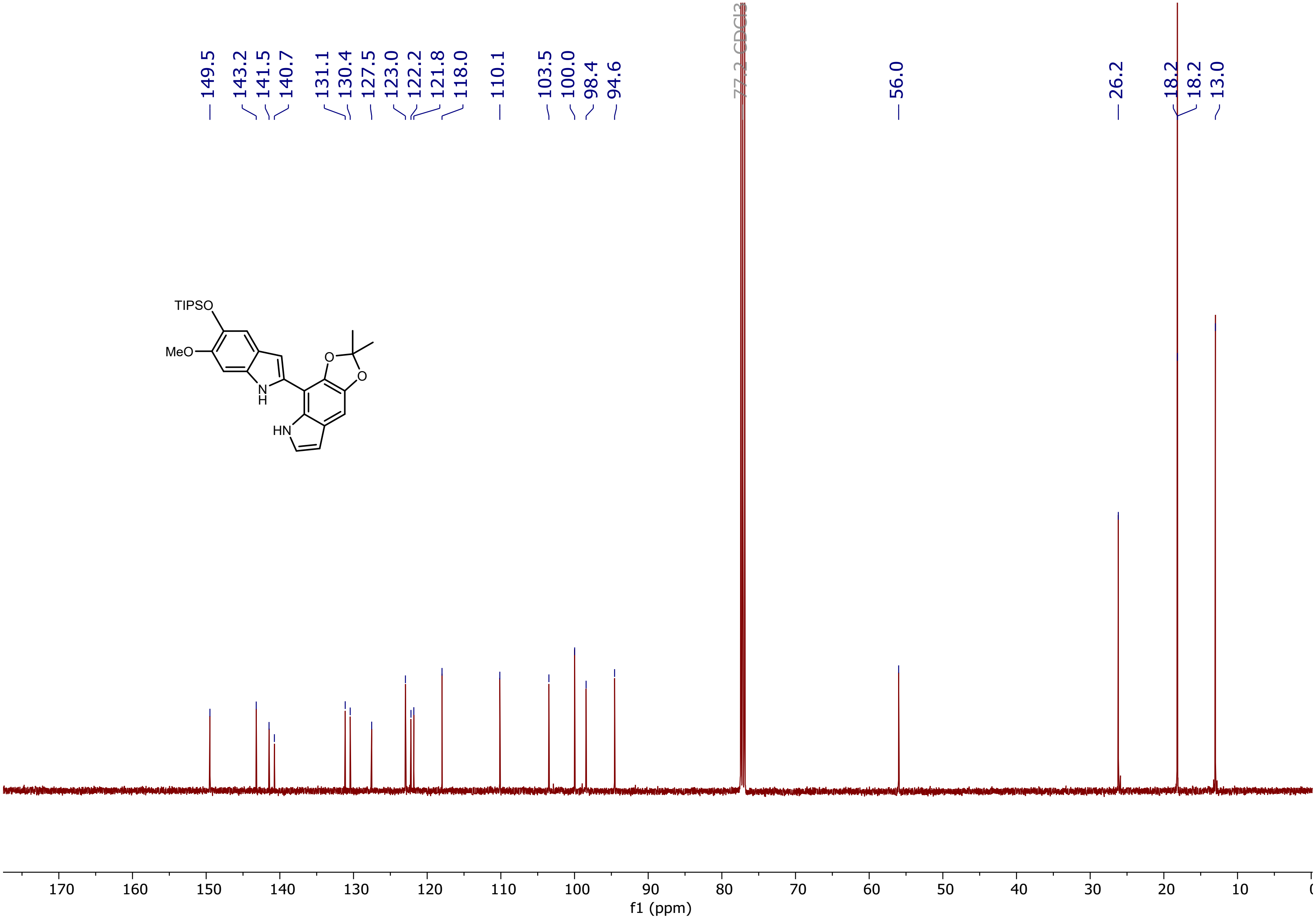

149.5
143.2
141.5
140.7
131.1
130.4
127.5
123.0
122.2
121.8
118.0
110.1
103.5
100.0
98.4
94.6
77.2 CDCl3
56.0
26.2
18.2
18.2
13.0
TIPSO
MeO
N
H
HN
O
O
f1 (ppm)

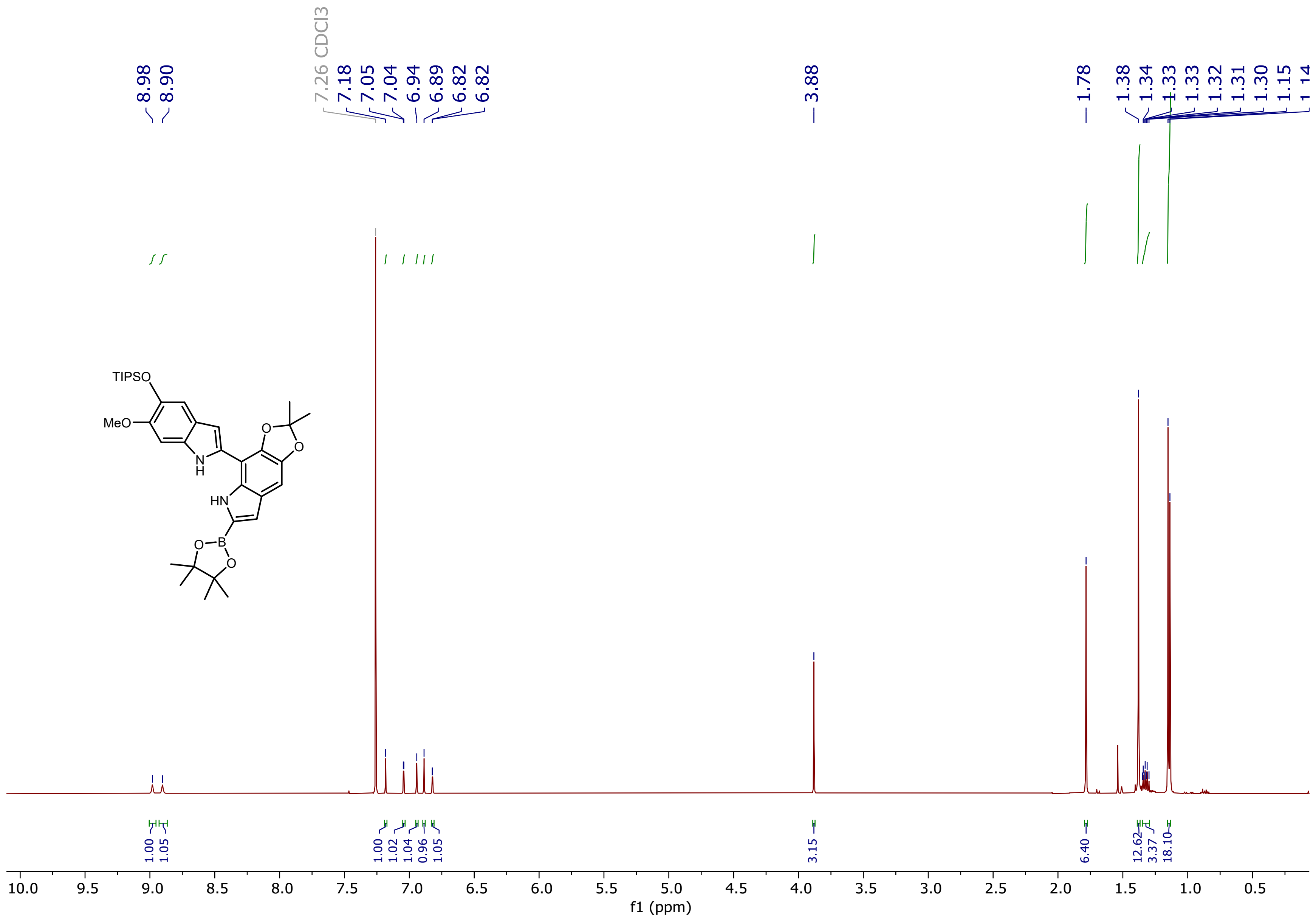
7.26 CDCl3
8.98
8.90
7.18
7.05
7.04
6.94
6.89
6.82
6.82
3.88
1.78
1.38
1.34
1.33
1.33
1.32
1.31
1.30
1.15
1.14
TIPSO
MeO
HN
O
B
1.00
1.05
1.00
1.02
1.04
0.96
1.05
3.15
6.40
12.62
3.37
18.10
f1 (ppm)

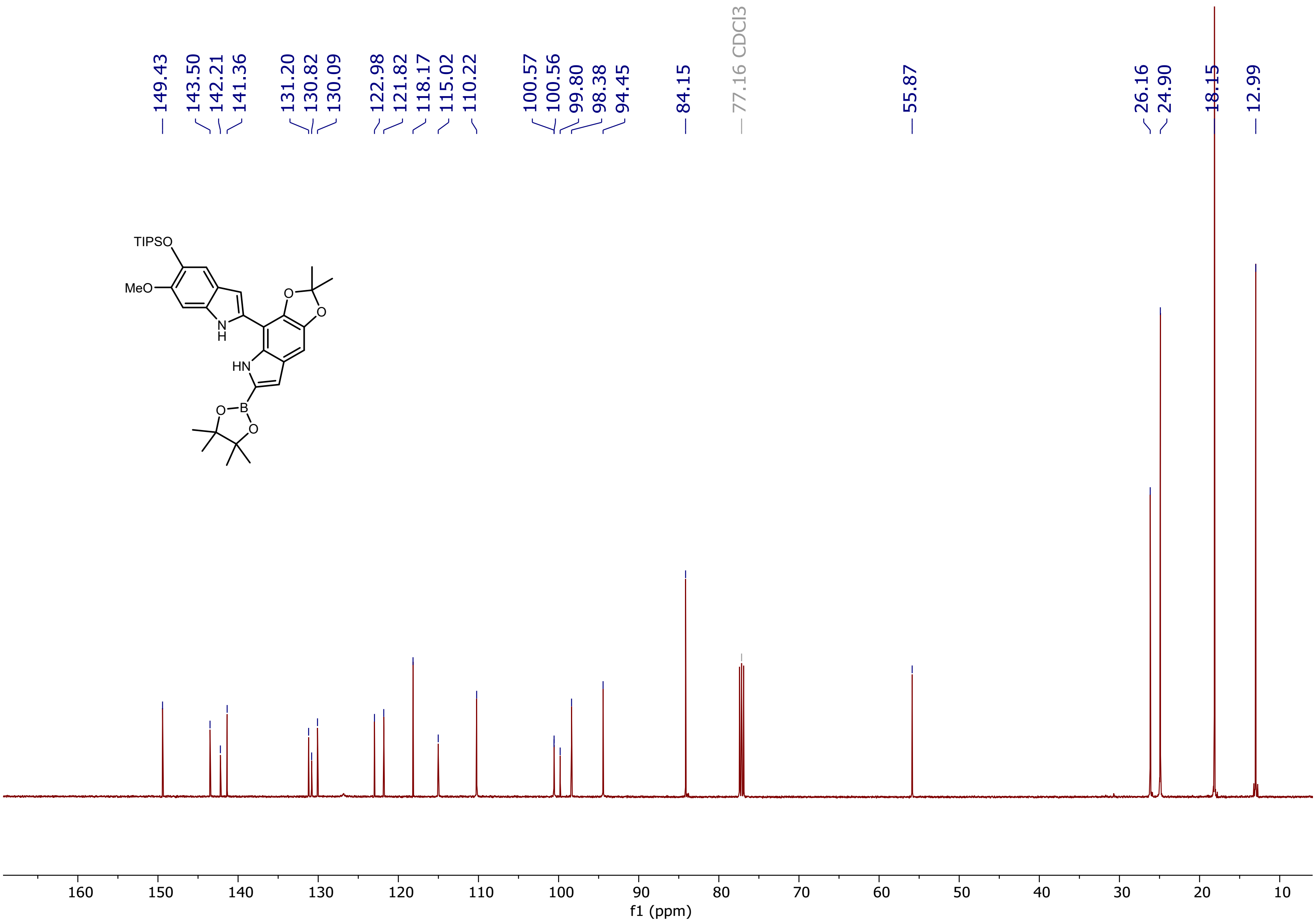

149.43
143.50
142.21
141.36
131.20
130.82
130.09
122.98
121.82
118.17
115.02
110.22
100.57
100.56
99.80
98.38
94.45
84.15
77.16 CDCl3
55.87
26.16
24.90
18.15
12.99
TIPSO
MeO
HN
O
B
f1 (ppm)

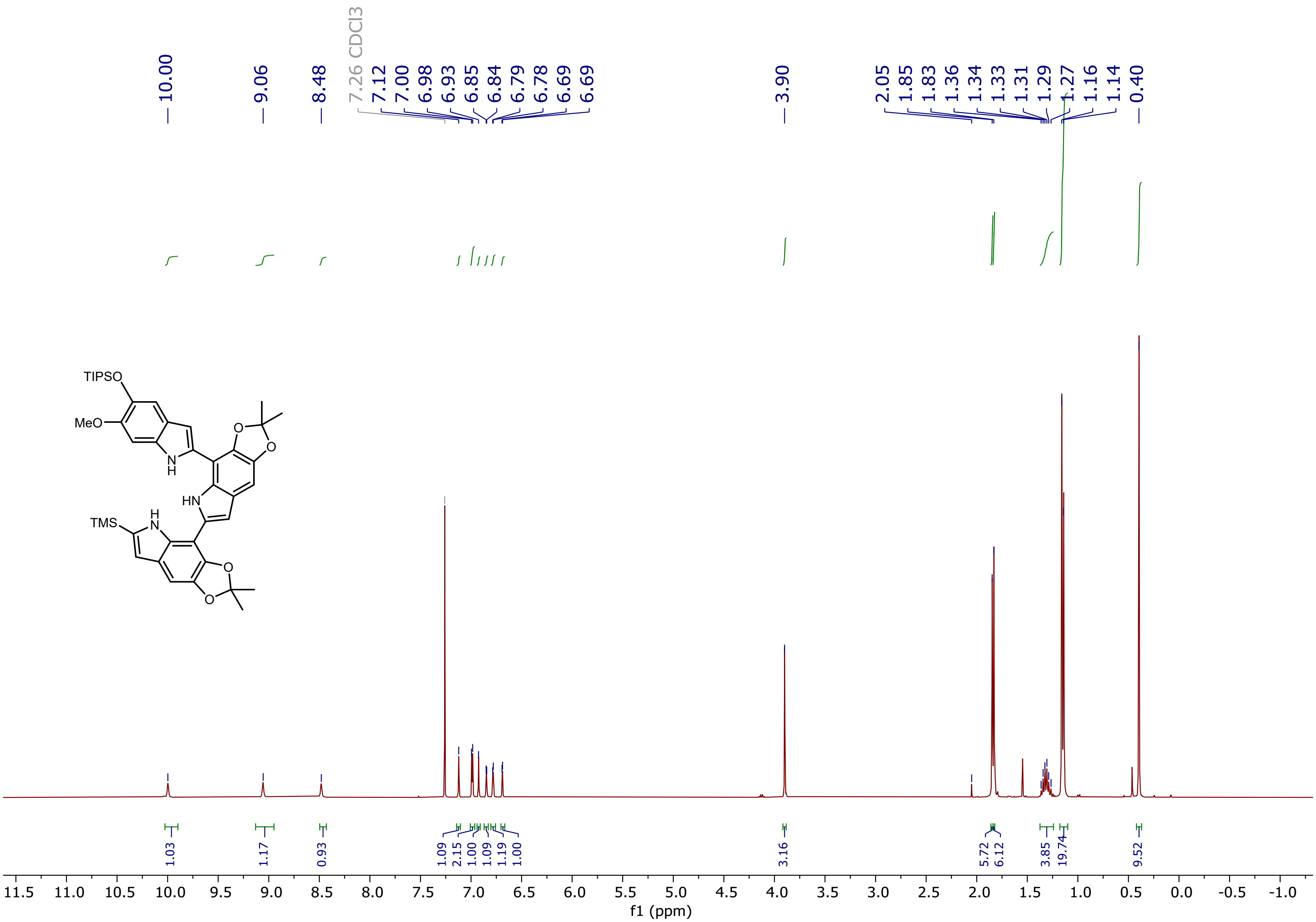
TIPSO
MeO
N
H
O
O
HN
TMS
H
N
O
O
10.00
9.06
8.48
7.26 CDCl3
7.12
7.00
6.98
6.93
6.85
6.84
6.79
6.78
6.69
6.69
3.90
2.05
1.85
1.83
1.36
1.34
1.33
1.31
1.29
1.27
1.16
1.14
0.40
1.03
1.17
0.93
1.09
2.15
1.00
1.09
1.19
1.00
3.16
5.72
6.12
3.85
19.74
9.52
f1 (ppm)

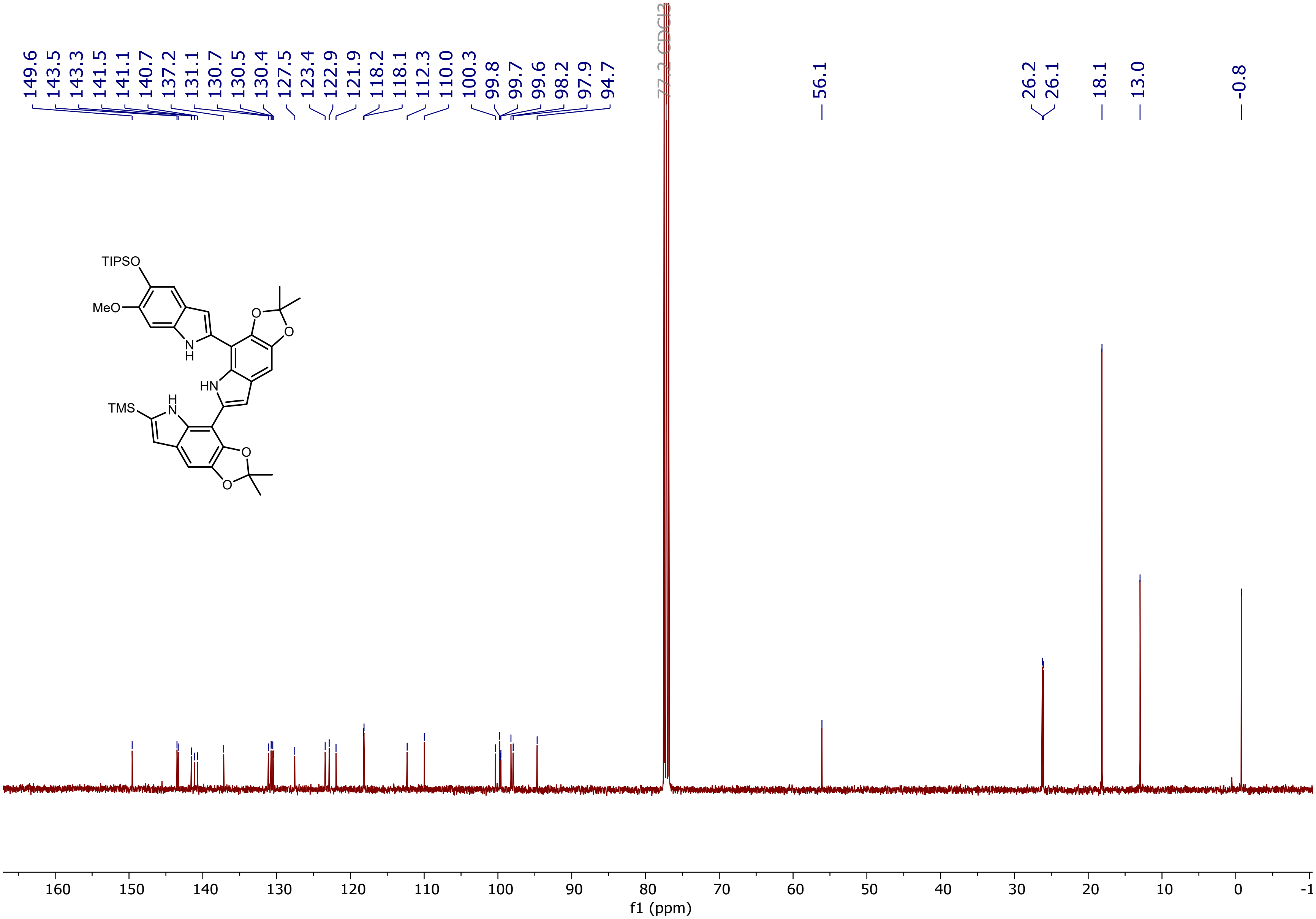
149.6
143.5
143.3
141.5
141.1
140.7
137.2
131.1
130.7
130.5
130.4
127.5
123.4
122.9
121.9
118.2
118.1
112.3
110.0
100.3
99.8
99.7
99.6
98.2
97.9
94.7
77.3 CDCl3
56.1
26.2
26.1
18.1
13.0
-0.8
TIPSO
MeO
N
H
O
O
HN
TMS
H
N
O
O
160
150
140
130
120
110
100
90
80
70
60
50
40
30
20
10
0
-1
f1 (ppm)

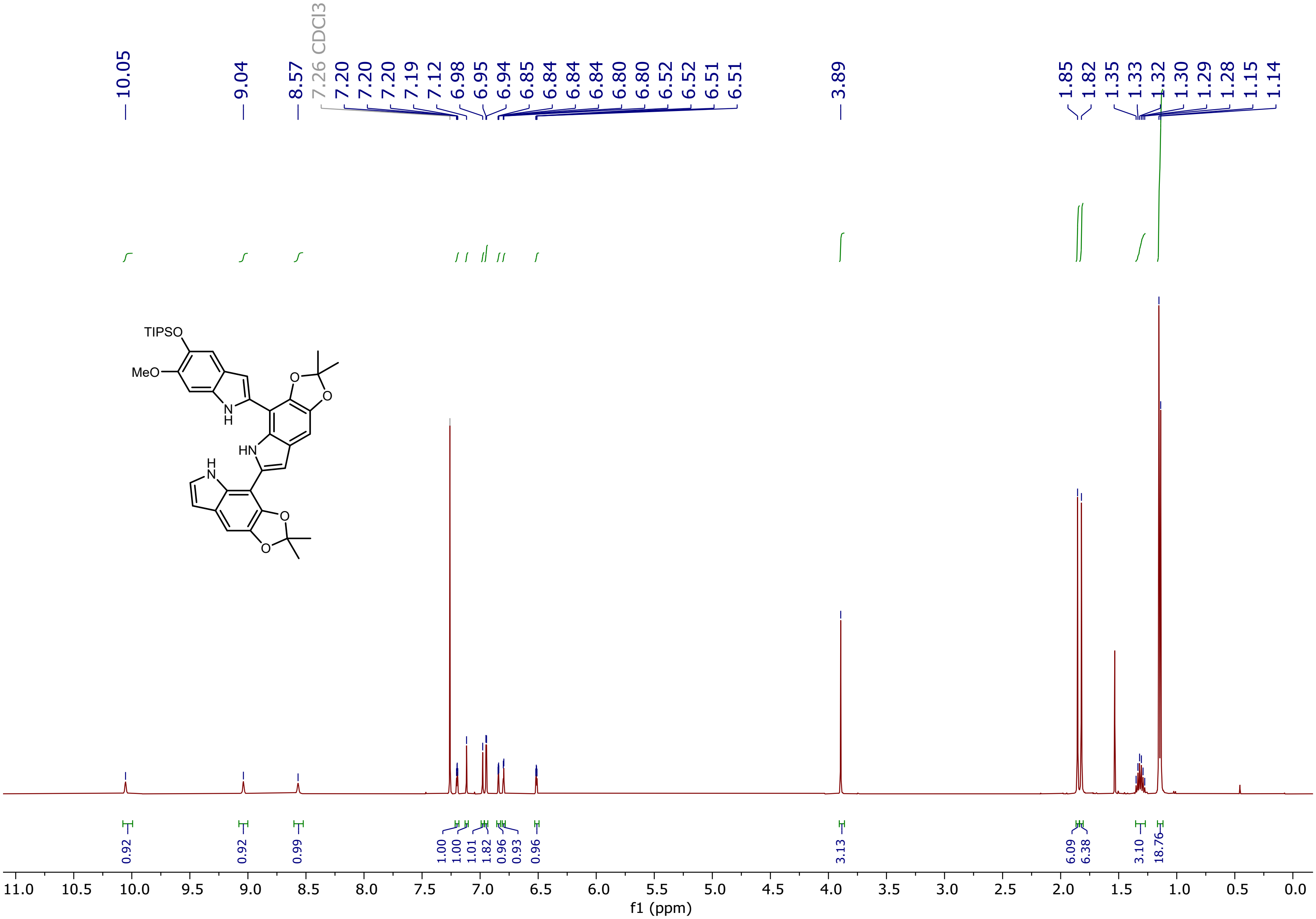
CDCl3
10.05
9.04
8.57
7.26 CDCl3
7.20
7.20
7.20
7.19
7.12
6.98
6.95
6.94
6.85
6.84
6.84
6.84
6.80
6.80
6.52
6.52
6.51
6.51
3.89
1.85
1.82
1.35
1.33
1.32
1.30
1.29
1.28
1.15
1.14
TIPSO
MeO
O
N
H
HN
0.92
0.92
0.99
1.00
1.00
1.01
1.82
0.96
0.93
0.96
3.13
6.09
6.38
3.10
18.76
11.0
10.5
10.0
9.5
9.0
8.5
8.0
7.5
7.0
6.5
6.0
5.5
5.0
4.5
4.0
3.5
3.0
2.5
2.0
1.5
1.0
0.5
0.0
f1 (ppm)

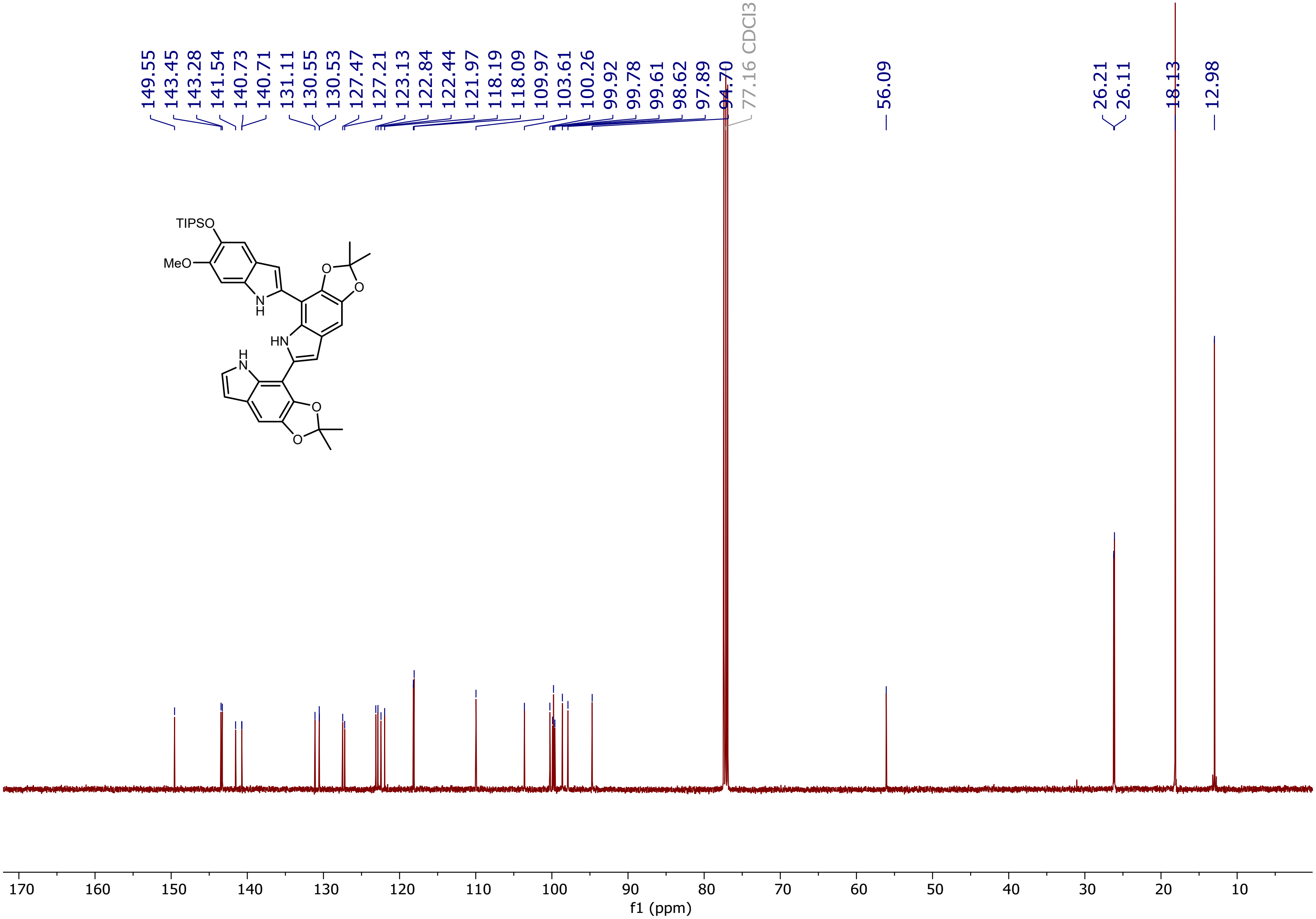
149.55
143.45
143.28
141.54
140.73
140.71
131.11
130.55
130.53
127.47
127.21
123.13
122.84
122.44
121.97
118.19
118.09
109.97
103.61
100.26
99.92
99.78
99.61
98.62
97.89
94.70
77.16 CDCl3
56.09
26.21
26.11
18.13
12.98
TIPSO
MeO
N
H
O
O
HN
H
N
O
O
170 160 150 140 130 120 110 100 90 80 70 60 50 40 30 20 10
f1 (ppm)

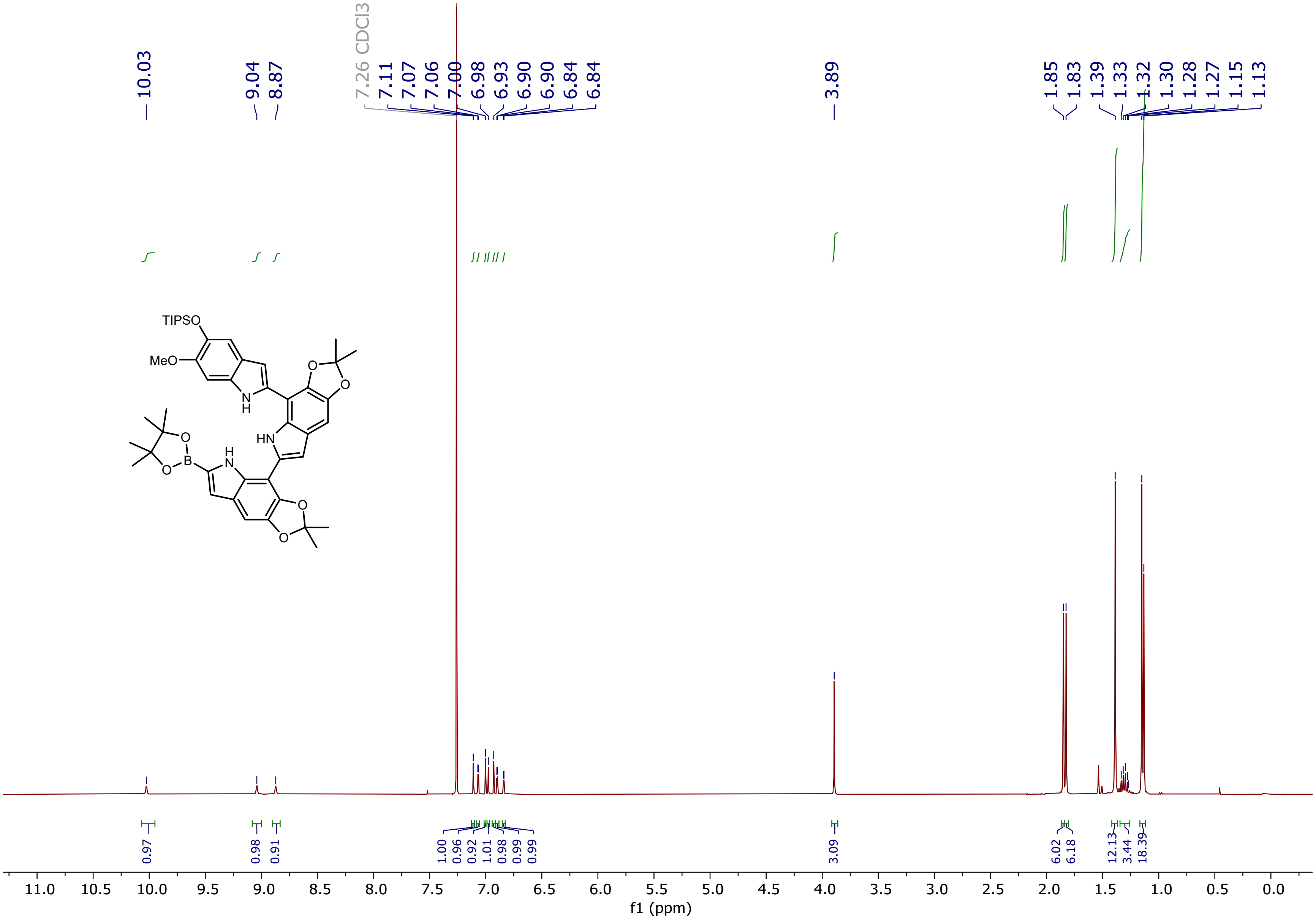
10.03
9.04
8.87
7.26 CDCl3
7.11
7.07
7.06
7.00
6.98
6.93
6.90
6.90
6.84
6.84
3.89
1.85
1.83
1.39
1.33
1.32
1.30
1.28
1.27
1.15
1.13
TIPSO
MeO
0.97
0.98
0.91
1.00
0.96
0.92
1.01
0.98
0.99
0.99
3.09
6.02
6.18
12.13
3.44
18.39
f1 (ppm)

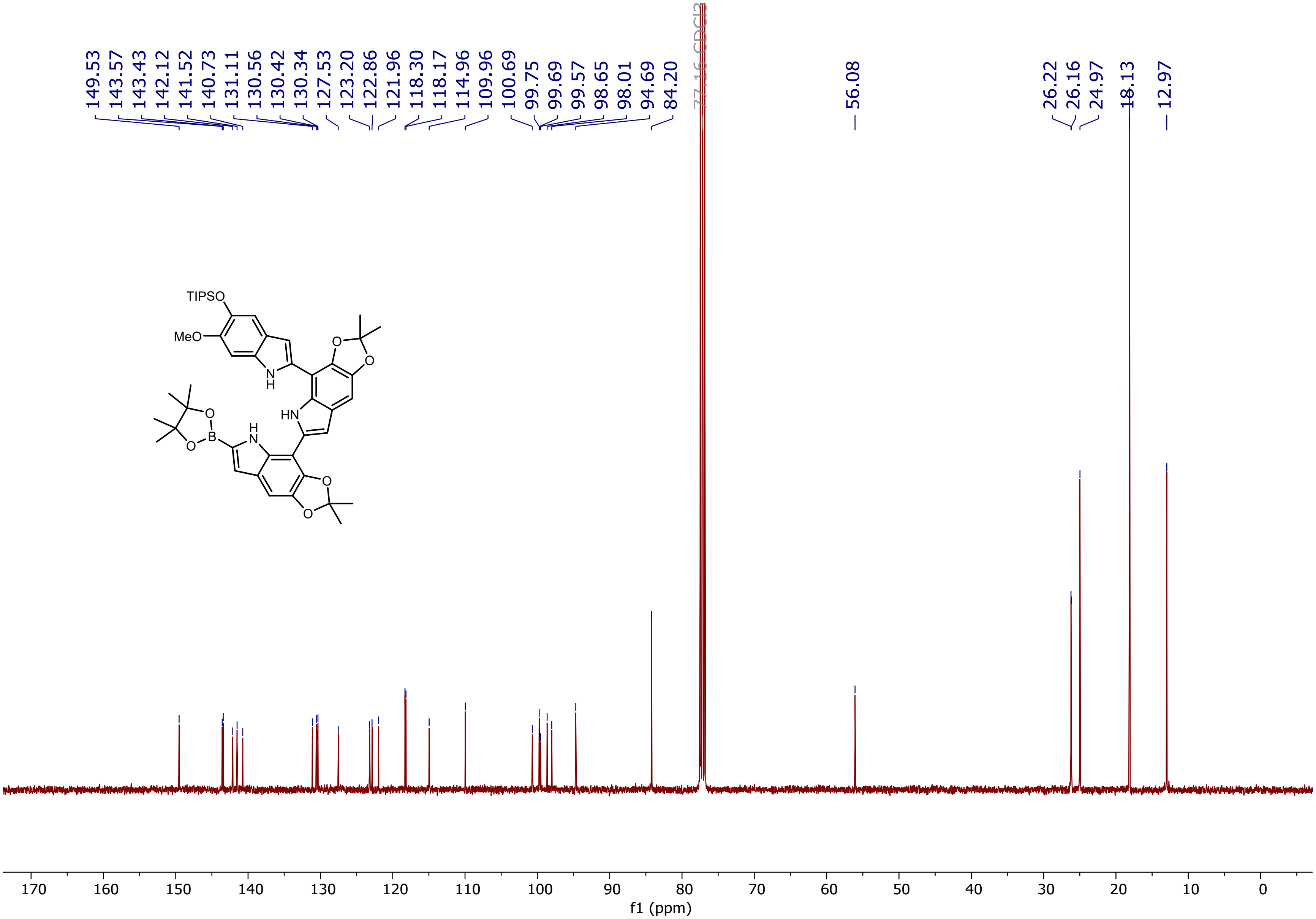
149.53
143.57
143.43
142.12
141.52
140.73
131.11
130.56
130.42
130.34
127.53
123.20
122.86
121.96
118.30
118.17
114.96
109.96
100.69
99.75
99.69
99.57
98.65
98.01
94.69
84.20
77.16 CDCl3
56.08
26.22
26.16
24.97
18.13
12.97
TIPSO
MeO
HN
O
B
f1 (ppm)
170
160
150
140
130
120
110
100
90
80
70
60
50
40
30
20
10
0

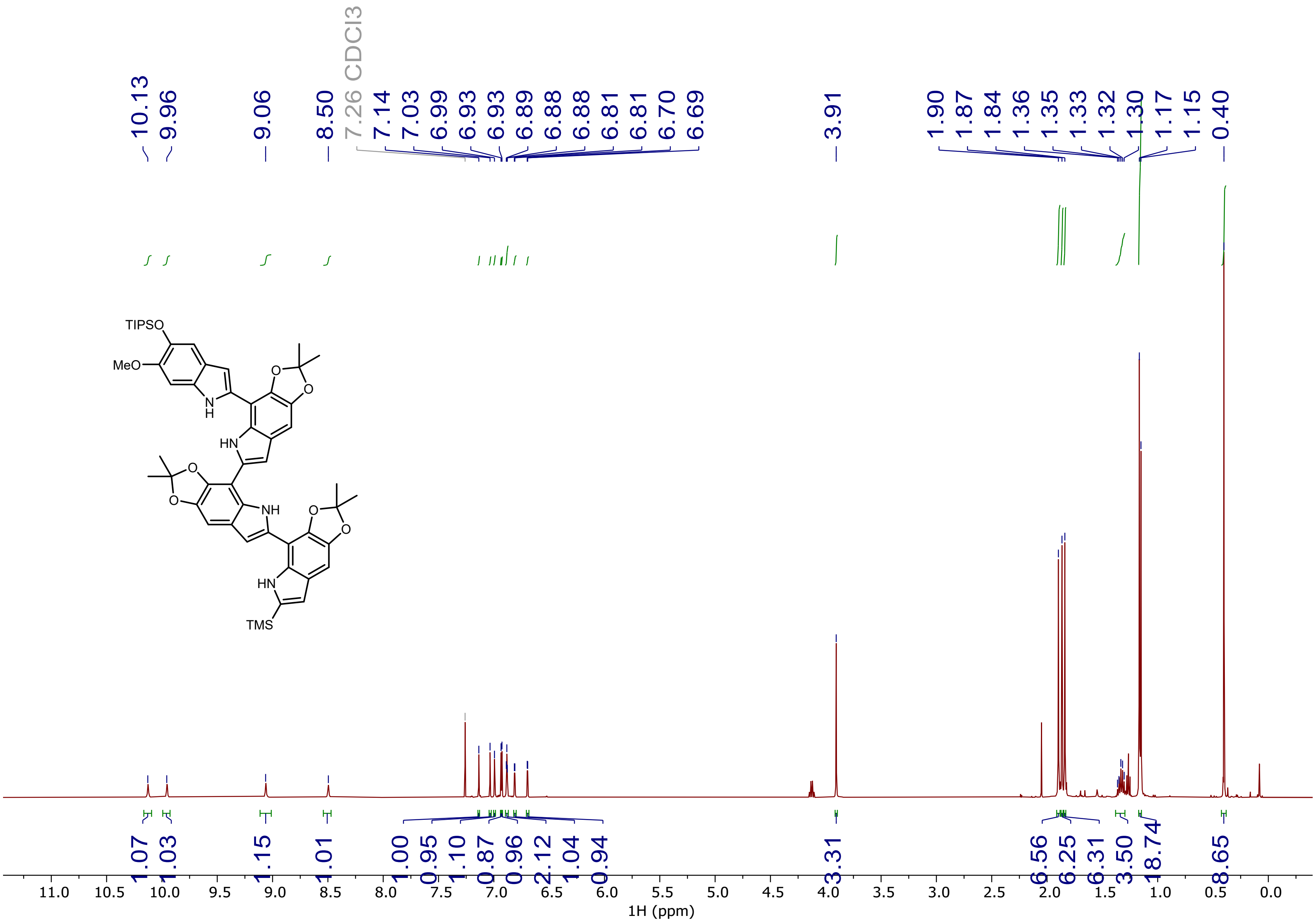
10.13
9.96
9.06
8.50
7.26 CDCl3
7.14
7.03
6.99
6.93
6.93
6.89
6.88
6.88
6.81
6.81
6.70
6.69
3.91
1.90
1.87
1.84
1.36
1.35
1.33
1.32
1.30
1.17
1.15
0.40
TIPSO
MeO
N
H
O
O
HN
O
O
NH
O
O
HN
TMS
1.07
1.03
1.15
1.01
1.00
0.95
1.10
0.87
0.96
2.12
1.04
0.94
3.31
6.56
6.25
6.31
3.50
18.74
8.65
1H (ppm)

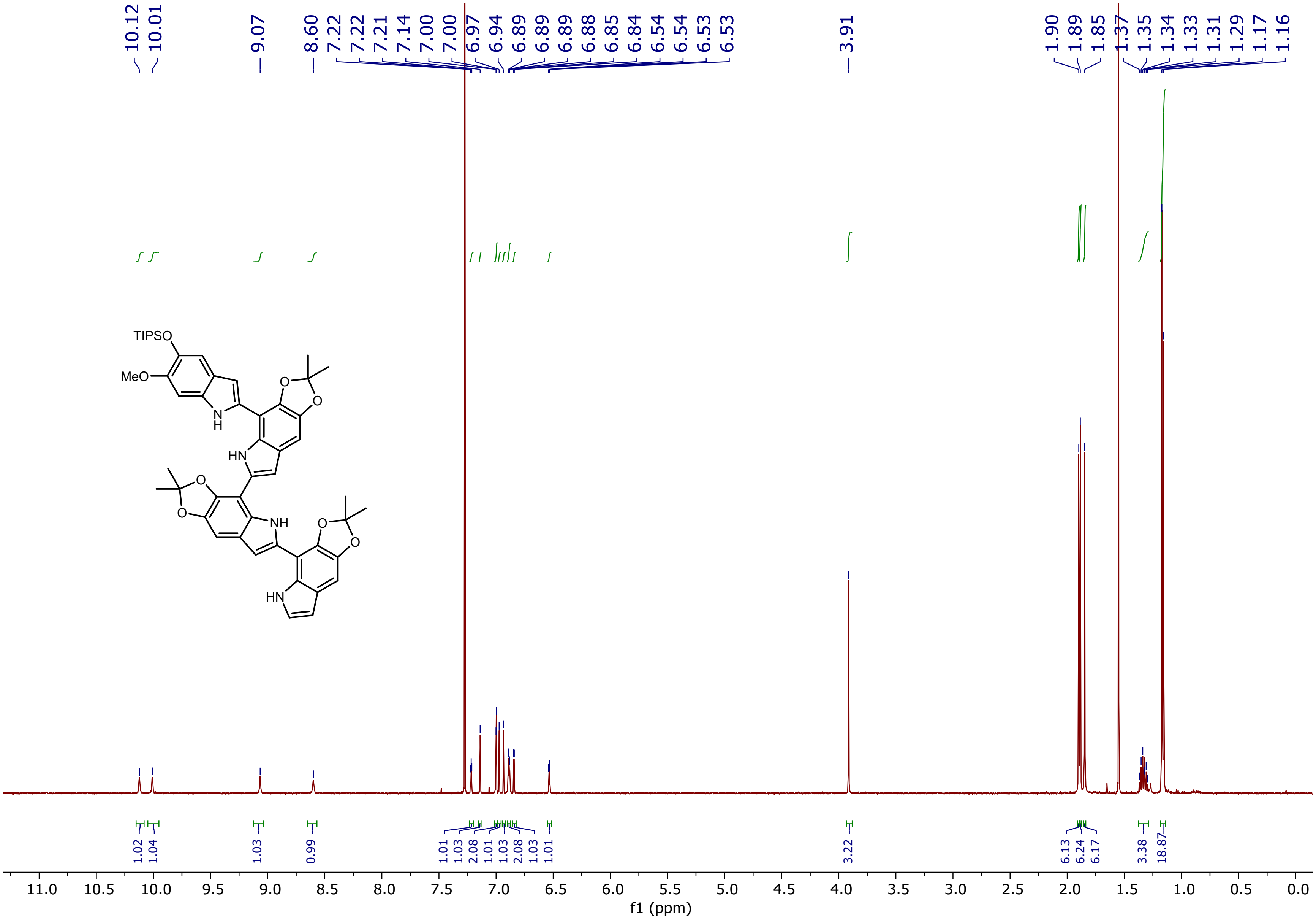

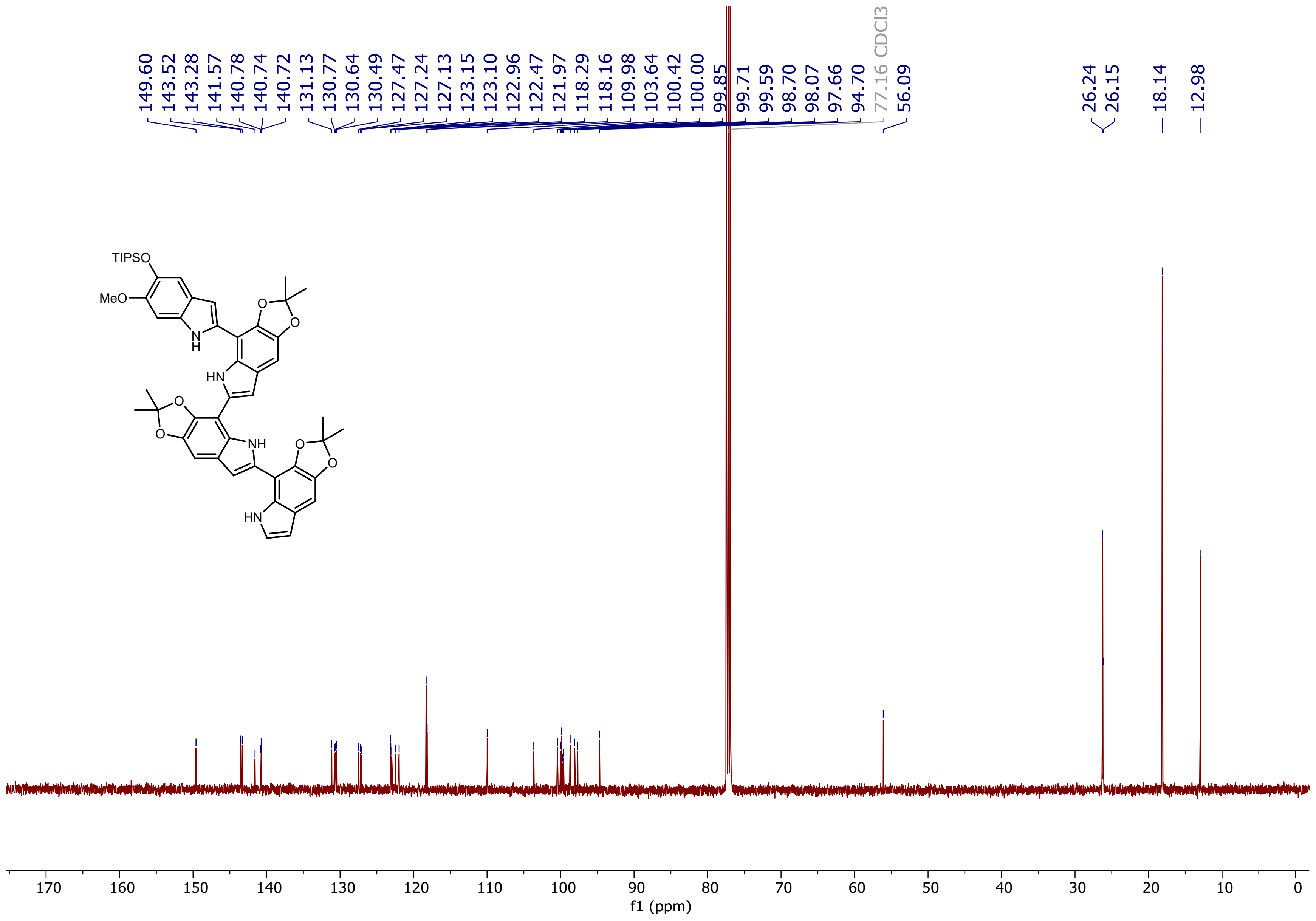
149.60
143.52
143.28
141.57
140.78
140.74
140.72
131.13
130.77
130.64
130.49
127.47
127.24
127.13
123.15
123.10
122.96
122.47
121.97
118.29
118.16
109.98
103.64
100.42
100.00
99.85
99.71
99.59
98.70
98.07
97.66
94.70
77.16 CDCl3
56.09
26.24
26.15
18.14
12.98
TIPSO
MeO
HN
NH
O
f1 (ppm)

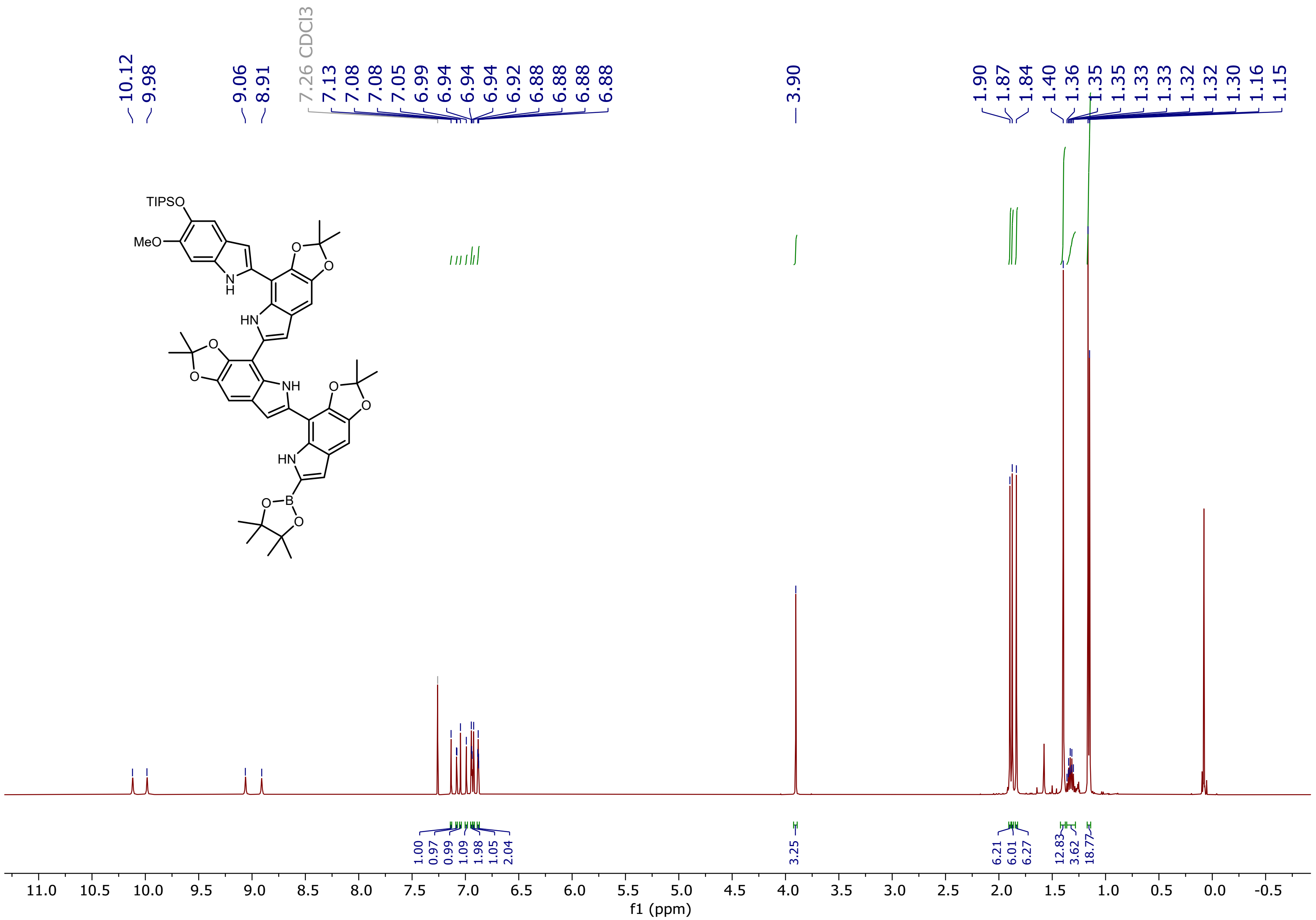
TIPSO
MeO
N
H
HN
O
O
NH
HN
O
B
O
10.12
9.98
9.06
8.91
7.26 CDCl3
7.13
7.08
7.08
7.05
6.99
6.94
6.94
6.94
6.92
6.88
6.88
6.88
6.88
3.90
1.90
1.87
1.84
1.40
1.36
1.35
1.35
1.33
1.33
1.32
1.32
1.30
1.16
1.15
1.00
0.97
0.99
1.09
1.98
1.05
2.04
3.25
6.21
6.01
6.27
12.83
3.62
18.77
11.0 10.5 10.0 9.5 9.0 8.5 8.0 7.5 7.0 6.5 6.0 5.5 5.0 4.5 4.0 3.5 3.0 2.5 2.0 1.5 1.0 0.5 0.0 -0.5
f1 (ppm)

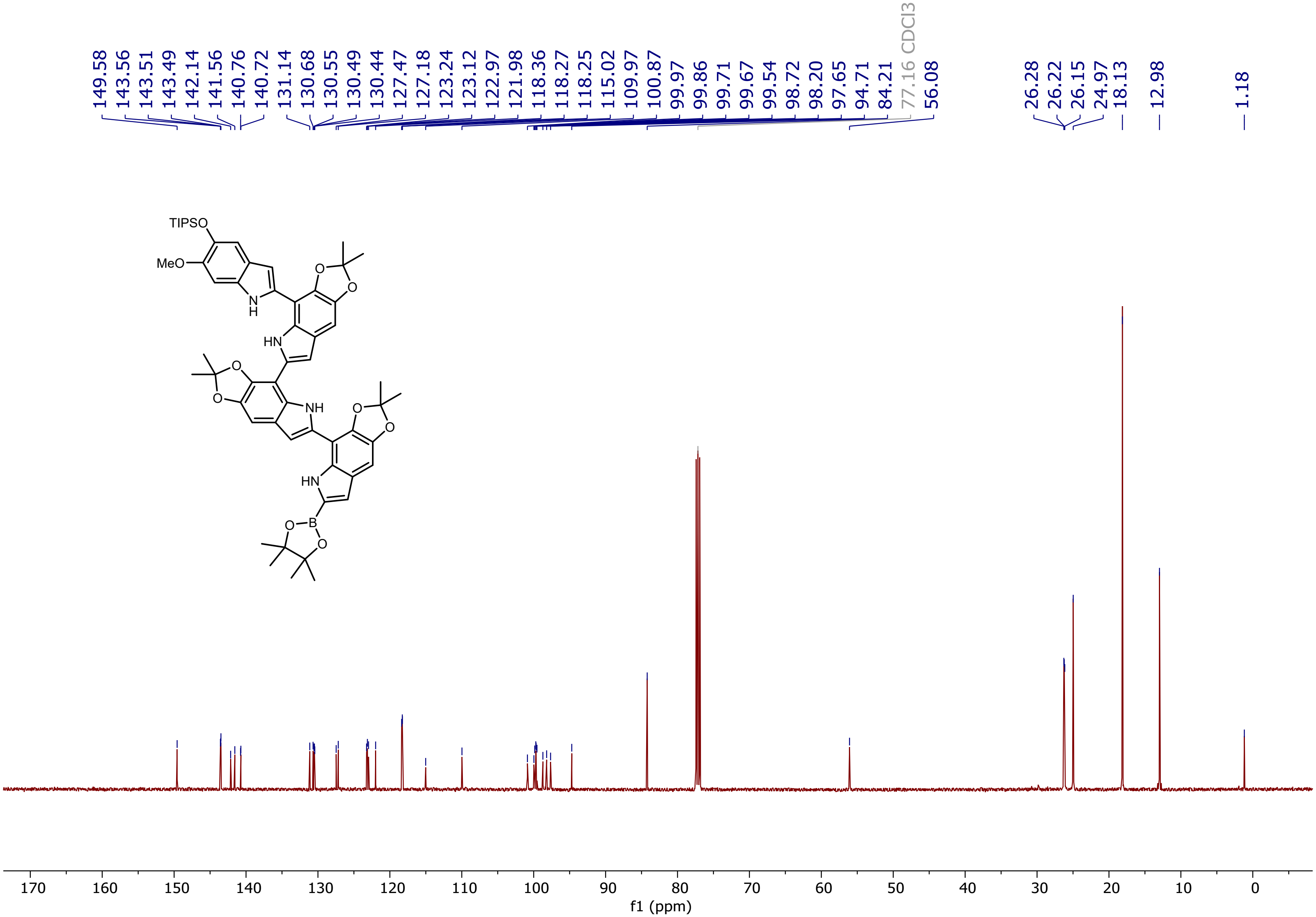
149.58
143.56
143.51
143.49
142.14
141.56
140.76
140.72
131.14
130.68
130.55
130.49
130.44
127.47
127.18
123.24
123.12
122.97
121.98
118.36
118.27
118.25
115.02
109.97
100.87
99.97
99.86
99.71
99.67
99.54
98.72
98.20
97.65
94.71
84.21
77.16 CDCl3
56.08
26.28
26.22
26.15
24.97
18.13
12.98
1.18
TIPSO
MeO
N
H
O
O
HN
O
O
NH
O
O
HN
O
B
O
170 160 150 140 130 120 110 100 90 80 70 60 50 40 30 20 10 0
f1 (ppm)

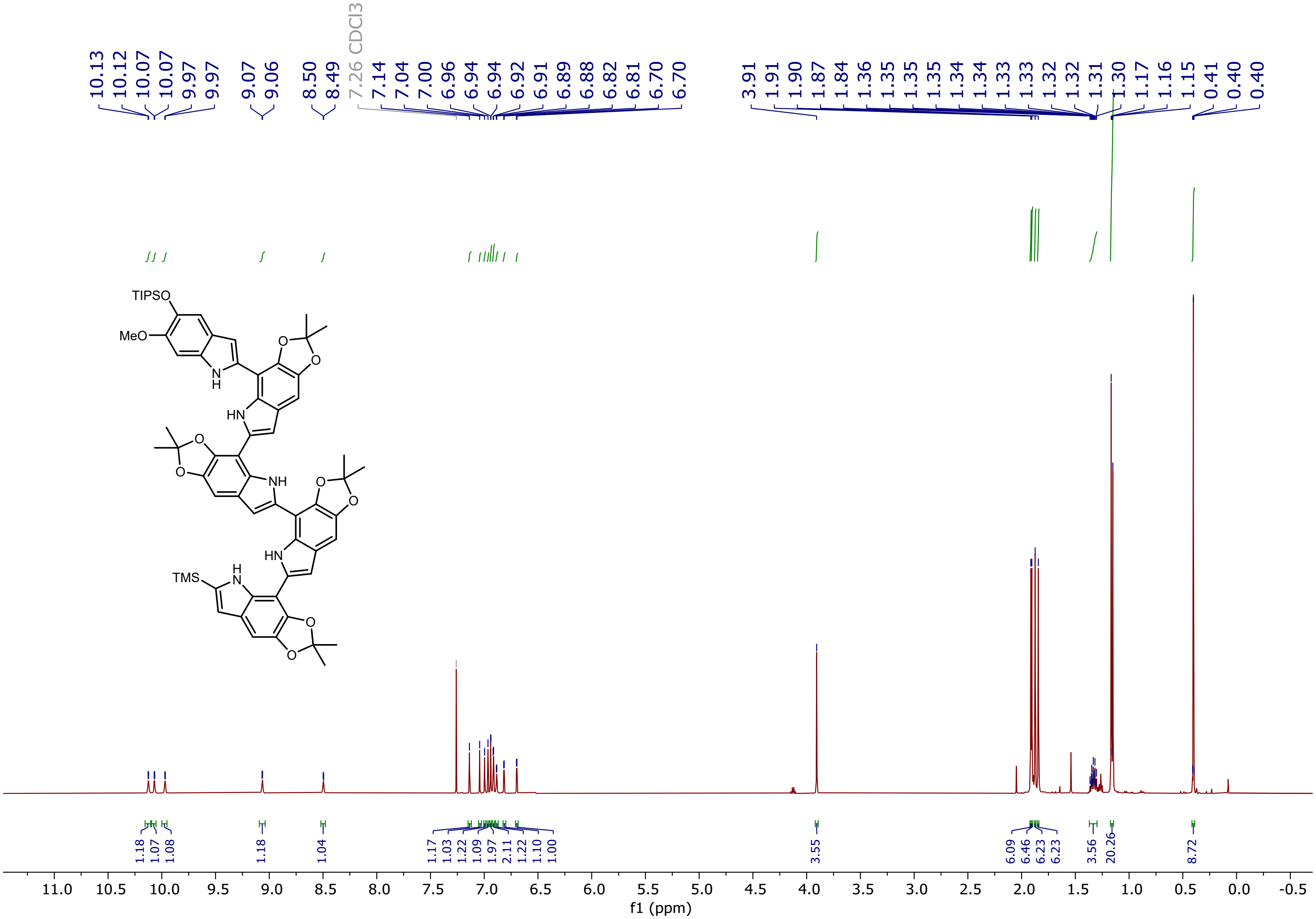
10.13 10.12 10.07 10.07 9.97 9.97 9.07 9.06 8.50 8.49 7.26 CDCl3 7.14 7.04 7.00 6.96 6.94 6.94 6.92 6.91 6.89 6.88 6.82 6.81 6.70 6.70 3.91 1.91 1.90 1.87 1.84 1.36 1.35 1.35 1.35 1.34 1.34 1.33 1.33 1.32 1.32 1.31 1.30 1.17 1.16 1.15 0.41 0.40 0.40
TIPSO
MeO
TMS
1.18 1.07 1.08 1.18 1.04 1.17 1.03 1.22 1.09 1.97 2.11 1.22 1.10 1.00 3.55 6.09 6.46 6.23 6.23 3.56 20.26 8.72
11.0 10.5 10.0 9.5 9.0 8.5 8.0 7.5 7.0 6.5 6.0 5.5 5.0 4.5 4.0 3.5 3.0 2.5 2.0 1.5 1.0 0.5 0.0 -0.5
f1 (ppm)

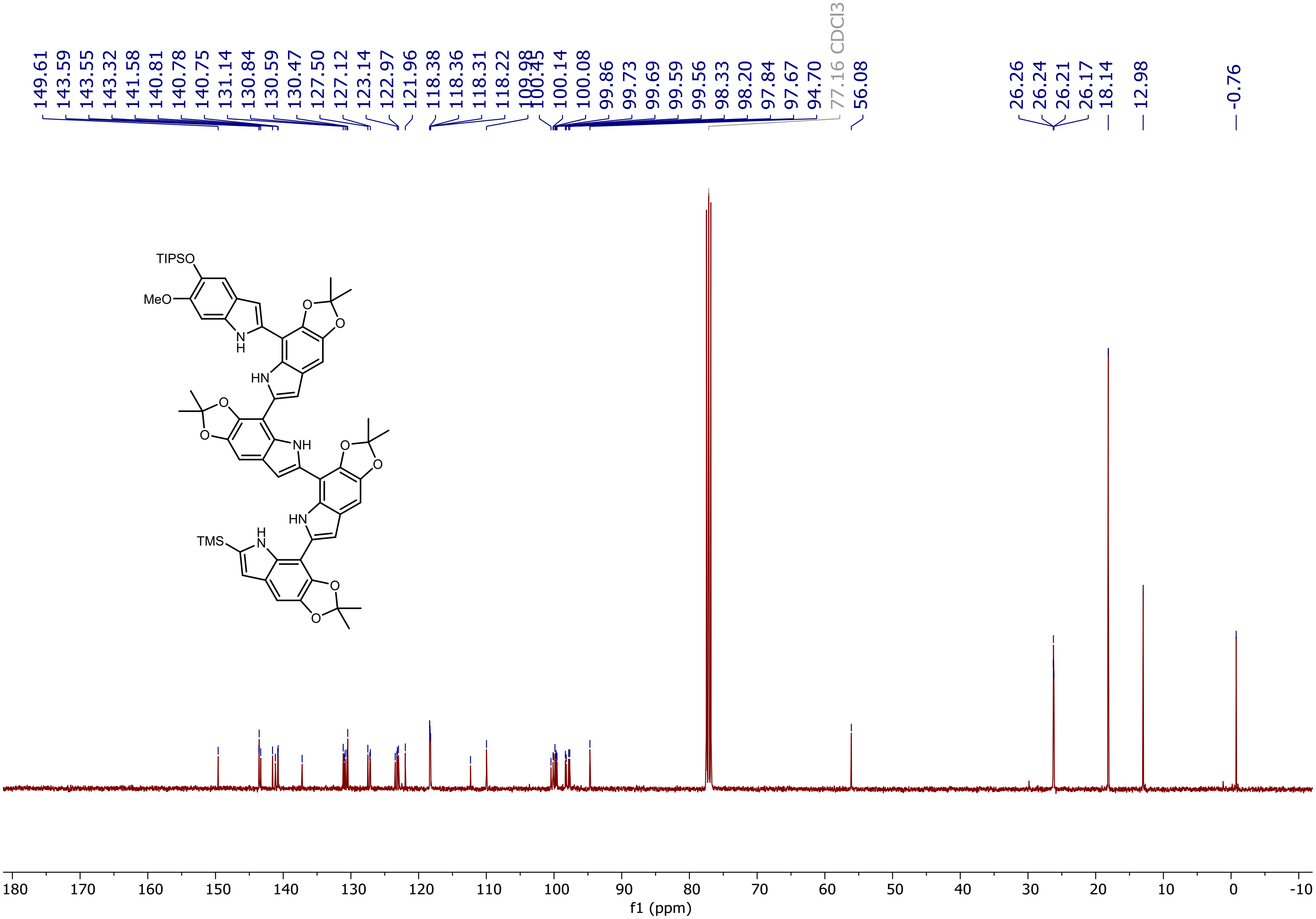
149.61
143.59
143.55
143.32
141.58
140.81
140.78
140.75
131.14
130.84
130.59
130.47
127.50
127.12
123.14
122.97
121.96
118.38
118.36
118.31
118.22
109.98
100.45
100.14
100.08
99.86
99.73
99.69
99.59
99.56
98.33
98.20
97.84
97.67
94.70
77.16 CDCl3
56.08
26.26
26.24
26.21
26.17
18.14
12.98
-0.76
TIPSO
MeO
N
H
HN
O
O
NH
HN
TMS
180 170 160 150 140 130 120 110 100 90 80 70 60 50 40 30 20 10 0 -10
f1 (ppm)

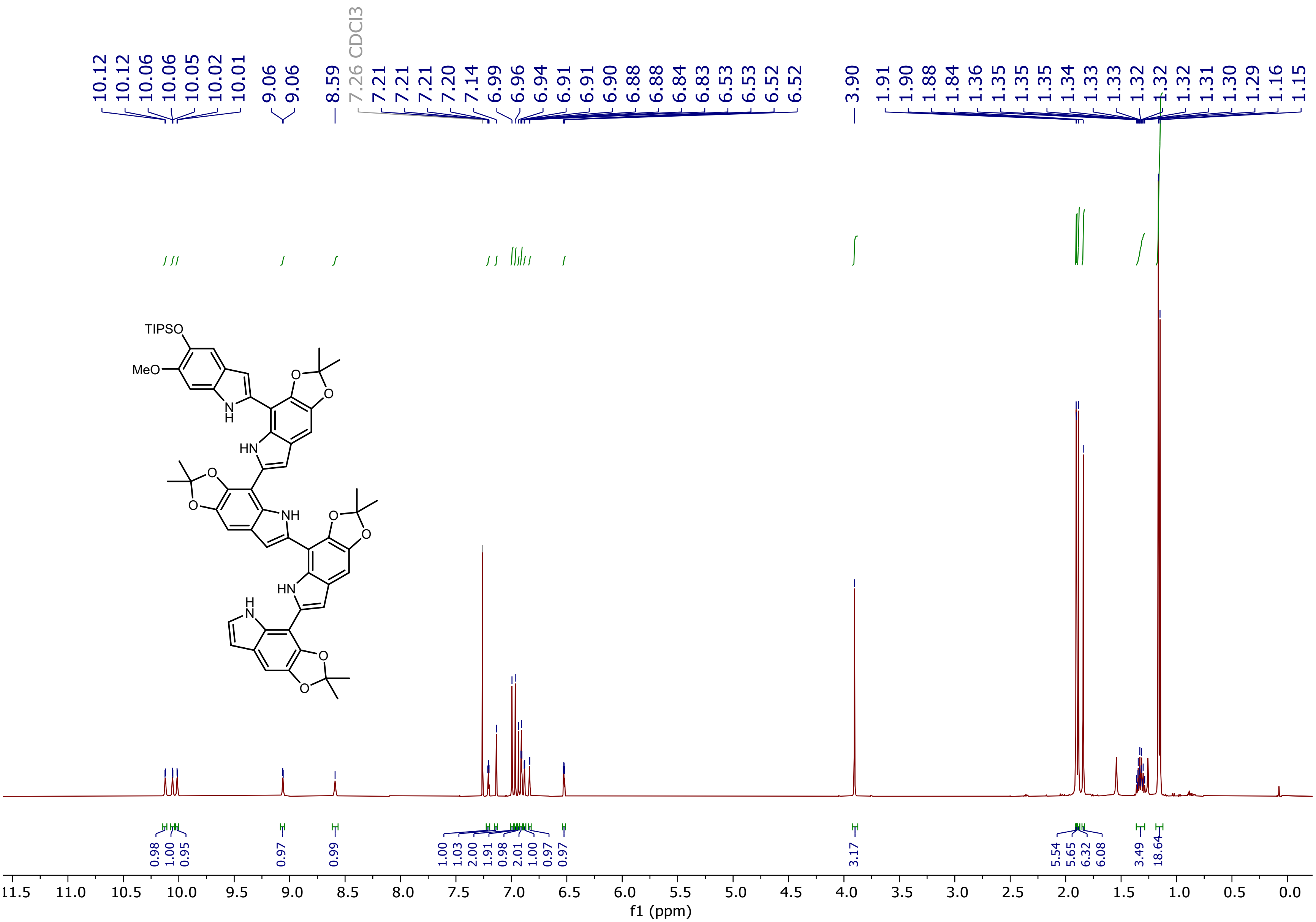
10.12
10.12
10.06
10.06
10.05
10.02
10.01
9.06
9.06
8.59
7.26 CDCl3
7.21
7.21
7.21
7.20
7.14
6.99
6.96
6.94
6.91
6.91
6.90
6.88
6.88
6.84
6.83
6.53
6.53
6.52
6.52
3.90
1.91
1.90
1.88
1.84
1.36
1.35
1.35
1.35
1.34
1.33
1.33
1.32
1.32
1.32
1.31
1.30
1.29
1.16
1.15
TIPSO
MeO
0.98
1.00
0.95
0.97
0.99
1.00
1.03
2.00
1.91
0.98
2.01
1.00
0.97
0.97
3.17
5.54
5.65
6.32
6.08
3.49
18.64
f1 (ppm)

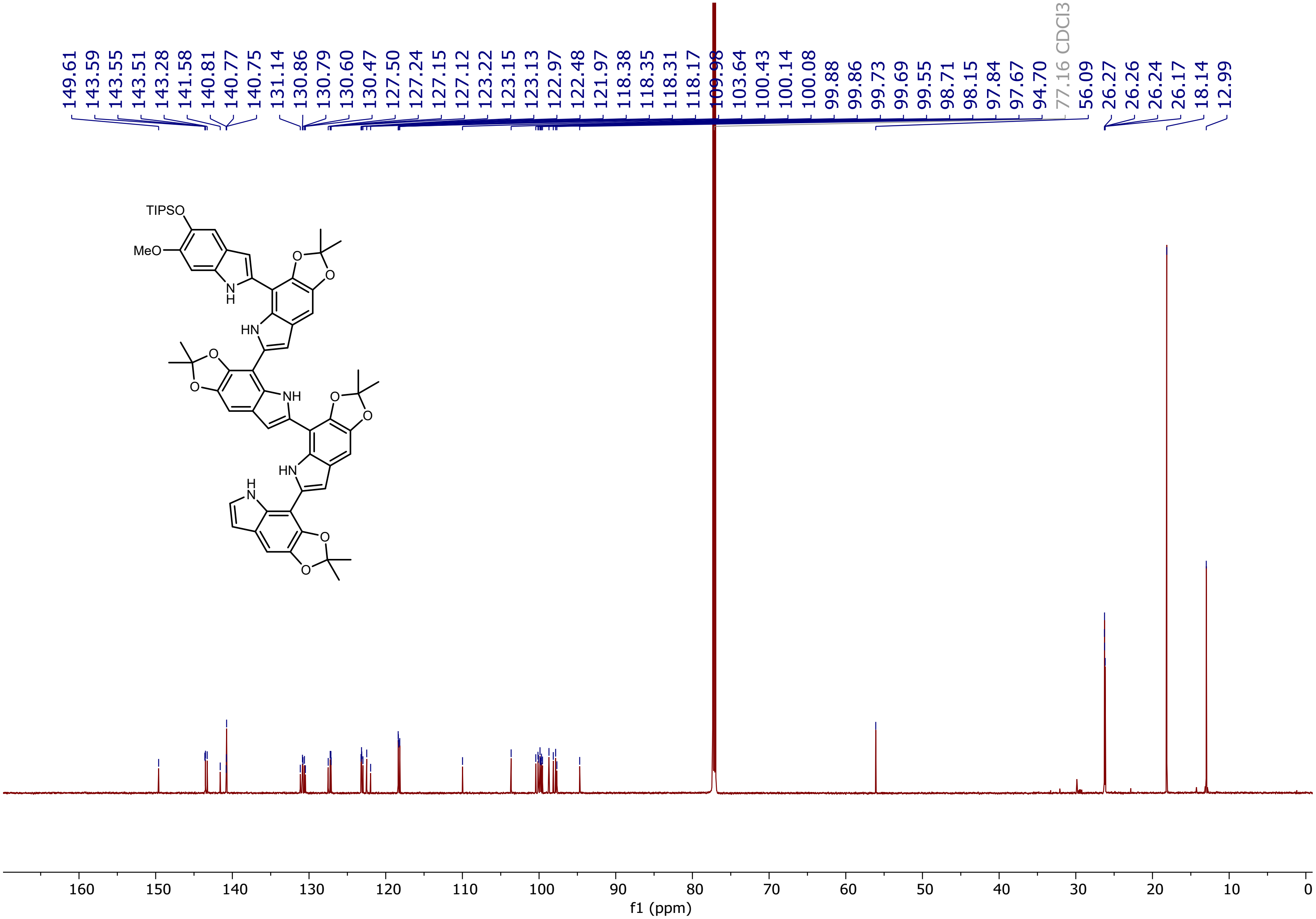
149.61
143.59
143.55
143.51
143.28
141.58
140.81
140.77
140.75
131.14
130.86
130.79
130.60
130.47
127.50
127.24
127.15
127.12
123.22
123.15
123.13
122.97
122.48
121.97
118.38
118.35
118.31
118.17
109.98
103.64
100.43
100.14
100.08
99.88
99.86
99.73
99.69
99.55
98.71
98.15
97.84
97.67
94.70
77.16 CDCl3
56.09
26.27
26.26
26.24
26.17
18.14
12.99
TIPSO
MeO
f1 (ppm)
